# Psoriasis drug development and GWAS interpretation through *in silico* analysis of transcription factor binding sites

**DOI:** 10.1186/s40169-015-0054-5

**Published:** 2015-03-19

**Authors:** William R Swindell, Mrinal K Sarkar, Philip E Stuart, John J Voorhees, James T Elder, Andrew Johnston, Johann E Gudjonsson

**Affiliations:** Department of Dermatology, University of Michigan School of Medicine, Ann Arbor, MI 48109-2200 USA

**Keywords:** AP-1, Decoy oligonucleotide, IRF, Motif, NF-kappaB, ODN, Position weight matrix, STAT

## Abstract

**Background:**

Psoriasis is a cytokine-mediated skin disease that can be treated effectively with immunosuppressive biologic agents. These medications, however, are not equally effective in all patients and are poorly suited for treating mild psoriasis. To develop more targeted therapies, interfering with transcription factor (TF) activity is a promising strategy.

**Methods:**

Meta-analysis was used to identify differentially expressed genes (DEGs) in the lesional skin from psoriasis patients (*n* = 237). We compiled a dictionary of 2935 binding sites representing empirically-determined binding affinities of TFs and unconventional DNA-binding proteins (uDBPs). This dictionary was screened to identify “psoriasis response elements” (PREs) overrepresented in sequences upstream of psoriasis DEGs.

**Results:**

PREs are recognized by IRF1, ISGF3, NF-kappaB and multiple TFs with helix-turn-helix (homeo) or other all-alpha-helical (high-mobility group) DNA-binding domains. We identified a limited set of DEGs that encode proteins interacting with PRE motifs, including TFs (*GATA3*, *EHF*, *FOXM1*, *SOX5*) and uDBPs (*AVEN*, *RBM8A*, *GPAM*, *WISP2*). PREs were prominent within enhancer regions near cytokine-encoding DEGs (*IL17A*, *IL19* and *IL1B*), suggesting that PREs might be incorporated into complex decoy oligonucleotides (cdODNs). To illustrate this idea, we designed a cdODN to concomitantly target psoriasis-activated TFs (i.e., FOXM1, ISGF3, IRF1 and NF-kappaB). Finally, we screened psoriasis-associated SNPs to identify risk alleles that disrupt or engender PRE motifs. This identified possible sites of allele-specific TF/uDBP binding and showed that PREs are disproportionately disrupted by psoriasis risk alleles.

**Conclusions:**

We identified new TF/uDBP candidates and developed an approach that (i) connects transcriptome informatics to cdODN drug development and (ii) enhances our ability to interpret GWAS findings. Disruption of PRE motifs by psoriasis risk alleles may contribute to disease susceptibility.

**Electronic supplementary material:**

The online version of this article (doi:10.1186/s40169-015-0054-5) contains supplementary material, which is available to authorized users.

## Background

Psoriasis is a chronic condition characterized by sharply demarcated skin lesions and increased risk of arthritis and cardiovascular disease. Lesion development is associated with excessive keratinocyte (KC) proliferation, altered KC differentiation, and an inflammatory infiltrate that includes innate and adaptive immune cells (e.g., neutrophils and T-cells) [1,2]. For moderate-to-severe psoriasis, effective biologic therapies have been developed to block specific cytokines (e.g., etanercept) or interfere with T-cell activation (e.g., efalizumab). The majority of patients, however, present with mild-to-moderate psoriasis, and for such patients biologic therapies do not provide a suitable first-line treatment. Even for those with moderate-to-severe psoriasis, biologic therapies are expensive [3], are not equally effective in all patients [4], and the long-term safety profile (>5 years) of immunosuppressive biologics is not fully established [5]. Development of new psoriasis treatments targeting more specific disease mechanisms has therefore remained a research priority [6]. Along these lines, transcription factors (TFs) are appealing as drug targets because they function as upstream regulators that can be inhibited locally, without necessarily targeting upstream cytokines or inflammatory processes [7]. Ideally, for instance, mild-to-moderate psoriasis could be controlled by effective topical agents, which rapidly resolve lesions by directly interfering with TFs and cellular pathways that promote excessive KC proliferation [8,9].

TFs contribute to immune cell activation in psoriasis as well as aberrant KC activity within lesions [10,11]. Genome-wide association studies (GWAS), for example, have identified variants near TF-encoding genes with significantly elevated frequency in psoriasis patients (e.g., *ETS1*, *IRF4*, *KLF4*, *RUNX3*, *STAT3*, *STAT5A* and *STAT5B*) [12]. Other TFs likely participate in lesion development through their role in KC proliferation (e.g., E2F and FOXM1), KC differentiation (e.g., TP63, KLF4 and AP-1), or immune cell activation (e.g., NF-κB). Topical agents may interfere with activation of these TFs in psoriasis, but may also have off-target effects limiting their efficacy (e.g., corticosteroids) [8,9]. To more specifically target one or multiple TFs, a promising approach may be to employ *cis*-element double-stranded decoy oligonucleotides (dODN), which mimic the DNA recognition site for a TF and thus attenuate its cellular activity [13,14]. In 1996, the first ODN-based therapy directed against E2F was approved by the Food and Drug Administration for treatment of neointimal hyperplasia in vein bypass grafts [13]. Since then, dODNs were shown to be effective for topical treatment of skin diseases, such as allergic contact dermatitis and wound fibrosis [15,16]. Indeed, a STAT3 dODN has already been demonstrated to resolve lesions in a psoriasis mouse model [17]. To design new dODN molecules for psoriasis, it is essential to have knowledge of the *cis*-element(s) recognized by TFs central to the disease process. For this purpose, studies that have compared gene expression in lesions and uninvolved (normal) skin from psoriasis patients are a valuable resource [18-24]. In such studies, genes with significantly altered expression in lesions can be identified (i.e., DEGs) and statistical approaches can be used to identify *cis*-regulatory elements overrepresented in upstream sequences of such genes [25]. These elements may then provide starting points for rational dODN design, essentially providing a direct pathway connecting bioinformatics to drug development.

Identifying TFs and *cis*-regulatory elements that drive psoriasis plaque formation should also illuminate our interpretation of GWAS findings [26]. GWASs have helped establish the immunological basis of psoriasis and have been valuable for identifying candidate disease mechanisms [27]. Despite this progress, most psoriasis susceptibility variants have been located in intergenic or intronic regions, suggesting that such variants confer increased disease risk through their effects on gene regulation [26,28]. To better characterize such mechanisms, it will ultimately be necessary to understand how TF-DNA interactions mediate psoriasis plaque development. By identifying DNA elements involved in these sequence-specific interactions, it will be possible to scan hits from psoriasis GWASs to identify variants that disrupt or engender such elements, potentially identifying sites at which allele-specific TF binding takes place to influence psoriasis risk [29,30]. This information could be further integrated with chromatin feature data for key cell types from the Human Encyclopedia of DNA Elements (ENCODE) project [31], with the ultimate goal of prioritizing non-coding risk variants for functional testing (e.g., by genome editing using CRISPR/Cas systems) [28,32].

The goals of this study are to use expression profiling data from psoriasis lesions to identify TFs and *cis*-regulatory elements mediating psoriasis plaque development. We identify differentially expressed genes (DEGs) by comparing lesional and uninvolved skin from a large meta-cohort of psoriasis patients (*n* = 237) [18-24]. To analyze DEGs, we assembled a dictionary of binding sites for human TFs and unconventional DNA-binding proteins (uDBPs) [33]. By screening this dictionary, we identified “psoriasis response elements” (PREs) overrepresented in sequences upstream of psoriasis DEGs. We show that only a fraction of DEGs encode proteins that recognize PREs, suggesting strong candidates for further study. We also demonstrate how PREs can be incorporated into complex dODN molecules as candidate psoriasis therapies, and we screen non-coding hits from psoriasis GWASs to identify PREs altered by risk alleles within enhancer regions. These findings provide novel and important steps forward in psoriasis drug development and the interpretation of GWAS hits at non-coding loci. The informatics pipeline developed in this study, moreover, could be applied to other diseases to similarly facilitate dODN design and GWAS interpretation.

## Methods

### Ethics Statement

All experiments were performed in accordance with Declaration of Helsinki principles. Samples were obtained from volunteer patients with informed written consent and protocols were approved by an institutional review board (University of Michigan, Ann Arbor, MI, IRB No. HUM00037994).

### Meta-analysis of psoriasis gene expression data

We pooled microarray data from nine studies that had evaluated lesional (PP) and uninvolved (PN) psoriasis skin (Gene Expression Omnibus accession IDs: GSE13355, GSE14905, GSE30999, GSE34248, GSE41662, GSE41663, GSE47751, GSE50790 and GSE51440) [18-24]. Eight studies utilized the Affymetrix Human Genome U133 Plus 2.0 array and one (GSE51440) utilized the “high throughput” version of this platform (Affymetrix HT HG-U133+ PM array plates). HT HG-U133+ PM array plates feature the same probe set content as U133 Plus 2.0 arrays, except mismatch probes are absent and most probe sets are filtered to include 9 probes (rather than 11) [34]. With respect to matching probe sets, fold-change estimates (PP/PN) were well-correlated between HT HG-U133+ PM and U133 Plus 2.0 arrays (0.47 ≤ *r*_s_ ≤ 0.65) (Additional file 1: part A). Data from both array platforms was therefore integrated in our analyses.

The initial pooled dataset included paired PP and PN samples from 248 patients. Affymetrix quality control metrics were calculated for each sample (i.e., average background, scale intensity factor, RNA degradation score, percentage of probe sets called present, NUSE median, NUSE IQR, RLE median and RLE IQR) (Additional file 1: parts B – I) [35-37]. For GSE51440 samples, it was not possible to calculate some QC metrics because the array design lacked mismatch probes (e.g., percentage of probe sets called present) (Additional file 1, parts B – I). For each dataset, QC metrics were converted into *Z*-scores and we removed samples with *Z*-scores greater than 3.5 in absolute value. This removed 9 samples from 9 patients, yielding a total of 239 patients. We then inspected median fold-change estimates (PP/PN) of genes most commonly elevated or repressed in psoriasis lesions (*n* = 239 patients; Additional file 1, part J). This identified two patients for which PP-increased genes were repressed and PP-decreased genes were increased. Since PP and PN labels might have been reversed during sample processing for these patients, these samples were removed to yield the final dataset upon which subsequent analyses were based (*n* = 237 patients). A two-dimensional principal component plot did not reveal extreme outliers (Additional file 1: parts K and L). Likewise, cluster analysis did not identify outliers and suggested good agreement between HT HG-U133+ PM array plates (GSE51440) and the U133 Plus 2.0 platform (Additional file 1: part M).

Normalized expression values for the 474 samples (237 PP and PN pairs) were calculated using robust multichip average (RMA) [38,39]. The array platform included probe sets corresponding to 19851 human genes, with most genes represented by multiple probe sets [40]. To limit redundancy, a single representative probe set was identified for each human gene. We preferentially chose as representatives those probe sets for which cross-hybridization was expected to be minimal [29]. If multiple probe sets were available for the same gene without any difference in cross-hybridization potential, we selected as a representative whichever probe set had the highest median expression across all 474 samples. This yielded 19851 probe sets (one per gene). Of these, we excluded 3734 from further analyses because they were not significantly expressed above background with respect to at least 10% of all samples (excluding GSE51440 samples generated from HT HG-U133+ PM arrays without mismatch probes). A probe set was considered expressed above background if there was a significant signal intensity difference between perfect match (PM) and mismatch probes (MM) (P < 0.05; Wilcoxon signed-rank test) [41]. For the remaining 16117 skin-expressed genes, the PP – PN difference in RMA expression intensity was calculated for each patient and we used the Wilcoxon rank sum test to assess whether the median difference was greater or less than zero (*n* = 237). Raw p-values were adjusted using the Benjamini-Hochberg method to control the false discovery rate (FDR) [42]. To meet criteria for differential expression, we required FDR < 0.05 with PP/PN fold-change greater than 1.50 or less than 0.67. We additionally required the median FC to be greater than 1 with respect to each of the 9 datasets included in our analysis (PP-increased DEGs) or less than 1 with respect to each dataset (PP-decreased DEGs).

### Motif dictionary

We assembled a dictionary of 2935 motifs representing empirically-determined binding affinities of human and/or mouse TFs and uDBPs. The 2935 motifs were selected from an initial set of 4378 motifs pooled from multiple sources, including the human protein-DNA interaction database (hPDI) [43], Jaspar [44], UniPROBE [45] and TRANSFAC Professional (release 2013.4) [46] (Additional file 2). We also included motifs derived from a recent analysis of ENCODE ChIP-Seq datasets [47,48], as well as one study that systematically investigated human TF binding preferences using high-throughput SELEX technology [49] (Additional file 2). UniPROBE and hPDI motifs were generated using cell-free systems with TF/uDBP fusion constructs cloned in yeast and printed onto protein microarrays [43] or hybridized to double-stranded DNA microarrays [45]. Other experiments were performed using various cell types, with many ENCODE ChIP-Seq datasets generated using five transformed cell lines (i.e., K562, GM12878, HepG2, H1-hESC and HeLa) [47,48].

Position frequency matrices (PFM) from each source were converted to position probability matrices (PPM). A pseudocount of 0.80 was used following the suggestion of Nishida et al. [50]. To trim PPM matrices, we removed columns at each flank until two consecutive columns with information content greater than 0.25 were encountered. In a small number of cases, matrices were heavily gapped and applying this criterion would have removed all columns. In these cases, positions were removed at each flank until one column was encountered with information content greater than 0.25. If even the less stringent trimming procedure engendered a matrix with fewer than 4 columns, the matrix was discarded.

We expected that some motifs in our initial set of 4378 would be redundant, since the databases we pooled are not entirely independent, and in some cases two independent experiments might yield similar motifs for a given protein. We therefore filtered the 4378 motifs to limit redundancy, preferentially retaining matrices with greater average information content. Filtering was carried out in two steps. First, we identified matrices of the same length with the same IUPAC consensus sequence. If two matrices with the same IUPAC consensus were found, we excluded whichever matrix had lower average information content, except when the average difference of values in PPM matrices exceeded 0.05. Based upon this criterion, we removed 715 matrices to yield a set of 3663. Secondly, we filtered out matrices that differed in length but which could, upon alignment, be shown to share a similar recognition sequence. For this purpose, inter-motif distances were calculated using Smith-Waterman alignment and the Pearson correlation coefficient (R package: MotIV, function: motifDistances) [51]. This identified groups of similar motifs (distance < 10^−14^) and for each group we selected the single motif with highest information content. This removed an additional 728 motifs to yield the final set of 2935 upon which further analyses were based.

The 2935 binding sites were recognized by TFs associated with 1422 unique human genes, most of which (1129) were associated with the Gene Ontology terms “TF activity”, “Cofactor activity”, or “DNA Binding” (Additional file 3). Of the 1422 genes, 1049 (74%) were included within the TFclass catalogue of human genes encoding TFs [52]. There were 447 TFs from the TFclass catalogue not represented by a motif in our dictionary. Despite this, the dictionary included motifs for TFs from each major DNA-binding domain superclass and class (e.g., zinc-coordinating C2H2, helix-turn-helix homeo and basic bHLH domains) [52] (Additional file 4). Cluster analysis demonstrated a high diversity of DNA recognition sequences among the 2935 binding site models (Additional file 5).

### Motif *k*-mer scores

The 2935 motifs were assigned *k*-mer scores reflecting the degree of sequence preference with respect to 3-mer (*n* = 64) and 4-mer (*n* = 256) words. *k*-mer scores were calculated using position probability matrices (PPM) for each motif and the following formula.

1 $$ k-\mathrm{m}\mathrm{e}\mathrm{r}\ \mathrm{score} = \max \left[ \min {{\left({p}_1,{p}_2,\dots, {p}_k\right)}_i}_{=1,\dots, m}\right] $$

For a given *k*-mer at PPM position *i*, the overall *k*-mer match score at position *i* was the lowest of the *k* probabilities associated with the *k* nucleotides. This lowest probability was recorded at each of the *m* possible PPM positions, and the overall match score for a given *k*-mer/PPM pairing was the highest of these minimal probabilities. This score was calculated using both 5′-3′ sequences for a given *k*-mer word, with the final assigned score equal to the higher of the two values. *k*-mer scores are thus [0, 1] probability values, with values near 1 indicating a motif’s strong preference for a given *k*-mer sequence. We here use *k*-mer word scores to visualize trends among sets of motifs, as well as to calculate inter-motif distances for clustering motifs using the HOPACH algorithm [53]. Using HOPACH, motifs highlighted by our analyses were assigned to separate groups, based upon correspondence of their *k*-mer scores and the Median/Mean Split Silhouette (MSS) criterion [53].

### Identification of motifs enriched in sequences upstream of psoriasis DEGs using generalized additive logistic regression models

Transcription start site (TSS) proximal sequences of protein-coding human genes were scanned for matches to the 2935 motifs (5 kb upstream – 500 bp downstream). Coding regions and sequence assembly gaps were masked. We did not mask repetitive sequences because prior work has demonstrated *in vivo* binding of such sequences by TFs [48,54]. For a given locus and PPM motif of width *m*, a correspondence score (*ψ*) was calculated using position weight matrices (PWMs), which were calculated from PPM nucleotide probabilities (*p*) and nucleotide background frequencies (*f*), as described in the following equation [55].

2 $$ \psi ={\displaystyle {\sum}_{\left(i=1\right)}^m log2}{\left(\frac{p}{f}\right)}_i $$

The correspondence *ψ* was calculated for each locus and a PWM motif match was called if this score exceeded 80% of the maximum score possible for a given PWM model (i.e., *ψ*/*ψ*_max_ ≥ 0.80; R package: Biostrings, R function: matchPWM) [25,55,56]. For scanning promoter regions, we used empirical background probabilities observed across all TSS-proximal sequences included in our analysis (A: 0.247, C: 0.251, G: 0.254, T: 0.248). All other sequence scans were performed using uniform background frequencies (i.e., 0.25 per nucleotide). Sequences were scanned using both 5′ to 3′ orientations for each PWM model and we summed the total number of matches obtained using both orientations. Overlapping matches were merged and not double-counted.

Semiparametric generalized additive logistic models (GAM) were used to identify those PWMs for which the number of matches to TSS-proximal sequences was significantly elevated among psoriasis DEGs [25]. For these analyses, we identified PWM models for which the number of matches was significantly elevated in (i) PP-increased DEGs as compared to all other skin-expressed genes, (ii) PP-decreased DEGs as compared to all other skin-expressed genes, and (iii) all psoriasis DEGs as compared to all other skin-expressed genes. GAM models included a 0–1 response variable indicating whether a gene belonged to the set of psoriasis DEGs, along with two covariates (*x*_1_ and *x*_2_) corresponding to the total number of TSS-proximal matches for a given PWM model (*x*_1_) and the length of non-masked sequence scanned for each gene (*x*_2_) [25]. Both covariates were log_10_-transformed and the significance of PWM enrichment was assessed based upon the *Z* statistic and p-value associated with *x*_1_. Separate GAM models were fit for all 2935 PWMs, with raw p-values adjusted using the Benjamini-Hochberg method [42]. A motif was classified as significantly enriched with respect to psoriasis DEGs if the FDR-adjusted p-value was less than 0.10 with *Z* statistic greater than zero.

### Risk allele effects on PWM matches at disease-associated SNP loci

Disease-associated SNPs may disrupt or engender TF/uDBP binding sites [30]. We thus identified 36 psoriasis-associated SNPs from a recent GWAS meta-analysis along with 536 SNPs in strong linkage disequilibrium (*r*^2^ ≥ 0.90) [12]. Linked SNPs were identified using PLINK and 1000 Genomes (phase 1) variant call format files [29,57]. This yielded a total of 572 disease-associated SNPs, and PWM match scores were calculated for each SNP with respect to risk (*ψ*_R_) and non-risk alleles (*ψ*_NR_). The difference in match scores was normalized by the maximum score possible for a given PWM matrix (*ψ*_max_), as shown in the following equation.

3 $$ \mathrm{Risk}\ \mathrm{allele}\ \mathrm{effect} = \frac{1}{\psi_{max}}\left({\psi}_R-{\psi}_{NR}\right) $$

Negative values thus denote SNP-motif combinations for which a risk allele is predicted to decrease affinity between a TF/uDBP and its recognition motif, whereas positive values denote combinations for which a risk allele is predicted to increase affinity. Quantified in this way, risk allele effects may be continuous, possibly strengthening or weakening PWM correspondence without altering a binding site (i.e., *ψ*/*ψ*_max_ < 0.80 for both risk and non-risk alleles). We thus additionally identified SNP-motif combinations for which the SNP is predicted to engender or disrupt a binding site, based upon the *ψ*/*ψ*_max_ threshold of 0.80. We defined a binding site as having been engendered by a risk allele if *ψ*_R_/*ψ*_max_ > 0.85 and *ψ*_NR_/*ψ*_max_ < 0.75. Conversely, we define a binding site as having been disrupted by a risk allele if *ψ*_R_/*ψ*_max_ < 0.75 and *ψ*_NR_/*ψ*_max_ > 0.85.

### Additional RNA-seq, microarray and ENCODE datasets

For selected genes, we used RNA-seq (GSE54456) to compare expression between 92 lesional skin samples from psoriasis patients (PP) and 82 normal skin samples from control subjects (NN) [58]. Raw sequence reads were downloaded, filtered, and mapped to the human genome (Ensembl GRCh37) following procedures described by Swindell et al. [59]. Expression responses of genes to cytokine treatments were evaluated using a panel of microarray experiments described previously [60]. Likewise, changes in the expression of genes across skin diseases were evaluated using microarray data from diseased and normal skin, as described in an earlier report [29]. To localize expression to anatomical skin compartments (dermis, basal epidermis or suprabasal epidermis), microarray data from normal skin sectioned by laser capture microdissection was used (GSE42114; Affymetrix Human Genome U133 Plus 2.0 array) [61]. Expression of genes in whole blood was also compared between psoriasis patients (*n* = 44) and control subjects (*n* = 30) (GSE55201; Affymetrix Human Genome U133 Plus 2.0 array) [62]. All Affymetrix data was normalized using robust multichip average (RMA) [38], except when raw data was unavailable, in which case it was necessary to use contributor-normalized expression values from GEO series matrix files. In all cases, significant gene expression differences were assessed using linear models and moderated *t* statistics (R package: limma, function: lmFit) [63]. Genome conservation scores (phastcons) and ENCODE peaks were retrieved from the UCSC browser using rtracklayer [64,65]. NHEK enhancers were identified in a prior study using multivariate hidden Markov models and combinatorial analysis of 15 chromatin states [66].

### Immunohistochemistry (IHC)

Lesional (PP) and uninvolved (PN) skin samples were obtained from 3 patients (European Caucasian ancestry) with informed written consent. Prior to biopsy collection, each patient was instructed to follow medication washout protocols as described previously [20]. Anti-EHF and anti-AVEN antibodies were obtained from Thermo-Scientific (cat no. PA5-30716) and Abnova (cat no. PAB13091), respectively. Diaminobenzidine staining of paraffin embedded tissue sections from both PP and PN skin was performed with 1:200 (EHF) or 1:400 (AVEN) antibody dilutions.

## Results

### Meta-analysis identifies differentially expressed genes and near-universal gene expression patterns in psoriasis lesions (*n* = 237 patients)

We used microarray data from seven prior studies to compare gene expression in lesional (PP) and uninvolved (PN) skin from psoriasis patients (*n* = 237) [18-24]. From among 16117 skin-expressed genes, we identified 1823 differentially expressed genes (DEGs) with significantly altered expression, including 1027 PP-increased DEGs (median FC > 1.50 and FDR < 0.05) and 796 PP-decreased DEGs (median FC < 0.67 and FDR < 0.05). Differential expression statistics for all 16117 skin-expressed genes are provided as supplemental data (Additional file 6).

PP-increased DEGs included late KC differentiation genes, such as *FAPB5*, *CALML5*, *TGM1*, *SPRR2G*, *SPRR3* and *LCE3D* (Additional file 7). Among all 237 patients, there was variability in expression shifts of genes expressed in the basal layer (*ITGA6*, *KRT5*, *KRT14*), granular layer and cornified envelope (*IVL*, *LOR*, *FLG*), and early KC differentiation genes (*KRT1*, *KRT10*, *DSG1*, *DSC1*) (Additional file 7). With respect to loricrin (*LOR*), for instance, expression decreased slightly on average (FC = 0.75; P = 2.0 × 10^−8^), but was reduced more than 0.26-fold in some patients (lowest 10%) while increased 2.59-fold in others (highest 10%) (Additional file 7). This reflects molecular-level heterogeneity among psoriasis lesions, which can only be discerned by studying a sufficiently large patient cohort [22,29].

We could not identify any genes with decreased expression in all 237 patients, but we identified 5 genes for which expression was increased in all 237 patients (*PI3*, *IL36G*, *KYNU*, *SERPINB13* and *WNT5A*) (Additional file 8: part A). These may be regarded as hallmark psoriasis genes for which expression is near-universally elevated in lesions. Using RNA-seq, we confirmed that expression of the 5 genes is elevated in lesions (*n* = 92) as compared to normal skin from non-psoriatic controls (*n* = 82) (Additional file 8: part B). The 5 genes were also induced in cultured KCs following treatment with TNF, IL-17A or the combination TNF + IL17A (Additional file 8: part C). The 5 genes did not exhibit a psoriasis-specific expression pattern, since their expression was also elevated in squamous cell carcinoma, Mediterranean spotted fever eschars and atopic eczema (Additional file 8: part D).

### Identification of “psoriasis response elements” (PREs) enriched in genomic sequences upstream of psoriasis DEGs

The shifts in gene expression we observed in psoriasis lesions are likely due, in part, to activation or repression of TF-mediated regulatory mechanisms. To understand which TFs/uDBPs and *cis*-regulatory elements have a dominant role, we screened 2935 position weight matrix (PWM) models (see Methods) to identify those for which matching motifs are most significantly enriched in genomic sequences upstream of the 1027 PP-increased DEGs, the 796 PP-decreased DEGs, and the complete set of all 1823 DEGs (PP-increased + PP-decreased). Altogether, this identified 126, 461 and 462 PWMs for which motifs were significantly enriched with respect to the PP-increased DEGs, PP-decreased DEGs, and the combined set of all DEGs, respectively (FDR < 0.10). We collectively refer to the set of DNA elements matching these significantly enriched motifs as “psoriasis response elements” (PREs).

### Combined analysis of differential expression and PREs highlights six transcription factor-encoding genes (*FOSL1*, *FOXM1*, *IRF1*, *SOX10*, *SOX8* and *GATA3*)

**Figure 1:**
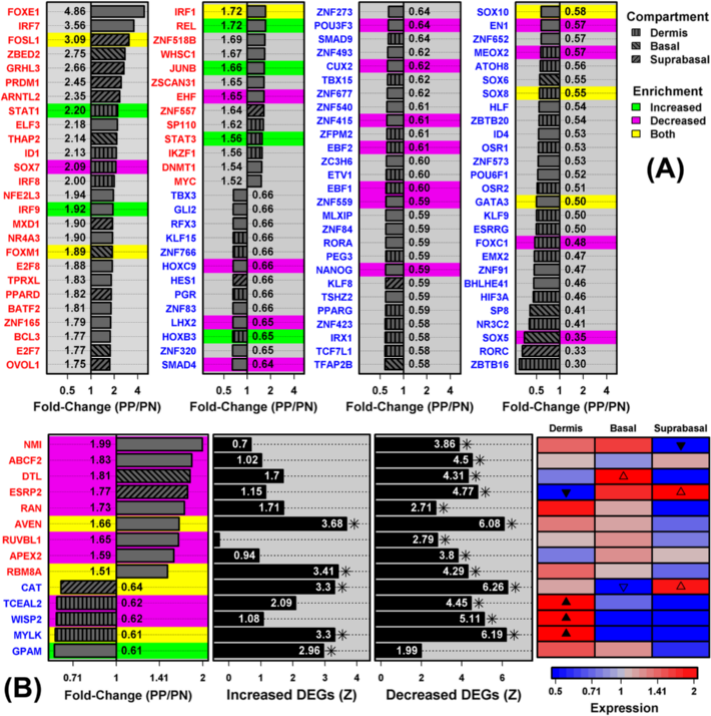
**Transcription factors and unconventional DNA-binding proteins with significantly altered expression in psoriasis lesions (** ***n*** **= 237 patients). (A)** Transcription factors. We identified 106 TF-encoding psoriasis DEGs and determined which of these recognize PRE motifs significantly enriched (FDR < 0.10) in sequences upstream of PP-increased DEGs (green background), PP-decreased DEGs (magenta), or both increased and decreased DEGs (yellow). For each DEG, laser capture microdissection data (GSE42114) was used to assess whether its expression is localized to a particular skin compartment (dermis, basal epidermis or suprabasal epidermis). **(B)** Unconventional DNA-binding proteins. We identified 14 DEGs encoding an uDBP that recognized a PRE motif significantly enriched (FDR < 0.10) in genomic sequences upstream of PP-increased DEGs (green background), PP-decreased DEGs (magenta), or both increased and decreased DEGs (yellow). The figure shows enrichment (*Z* statistic) for each uDBP motif with respect to sequences upstream of PP-increased and PP-decreased DEGs, respectively, as well as relative expression in dermis, suprabasal epidermis and basal epidermis (laser capture microdissection; GSE42114).

We identified many TF-encoding genes as differentially expressed in psoriasis lesions, but only a fraction of these encode TFs interacting with PREs. Of 1823 DEGs, 106 were included within the TFclass database of TF-encoding genes (39 PP-increased and 67 PP-decreased) (Figure 1A). These 106 TF-encoding DEGs were more likely to interact with PRE motifs than other TF-encoding non-DEGs (P ≤ 0.015; Additional file 9). Overall, 28 of the 106 interacted with PREs, with the direction of differential expression often matching the pattern of motif enrichment. TFs encoded by PP-decreased DEGs, for instance, more commonly interacted with PREs enriched in sequences upstream of PP-decreased DEGs (14 of 67), but less commonly interacted with PREs enriched in sequences upstream of PP-increased DEGs (1 of 67) (Figure 1A). We identified 6 TF-encoding DEGs interacting with PREs enriched in sequences upstream of PP-increased DEGs as well as PP-decreased DEGs (*FOSL1*, *FOXM1*, *IRF1*, *SOX10*, *SOX8* and *GATA3*) (Figure 1A). We identified 14 DEGs that encode uDBPs interacting with PREs (Figure 1B). Of these, 4 recognized a PRE enriched in sequences upstream of both PP-increased and PP-decreased DEGs (*AVEN*, *RBM8A*, *CAT* and *MYLK*; Figure 1B).

For nearly all TFs/uDBPs interacting with PREs, we confirmed differential expression in psoriasis lesions using RNA-seq (*n* = 92 patients vs. *n* = 82 normal controls; Additional files 10 and 11). Several TFs/uDBPs additionally showed altered expression in blood from psoriasis patients (increased: *JUNB*, *STAT3*, *DTL*, *CAT*; decreased: *CUX2*, *ZNF559*, *AVEN*, *RUVBL1*, *RAN*; Additional files 10 and 11). IHC staining was used to evaluate the distribution of ETS homologous factor (*EHF*) and apoptosis caspase activation inhibitor (*AVEN*) in PP and PN skin (Additional files 10 and 11). EHF is a differentiation-associated transcriptional repressor that interacts with 5-TTCCGA/TCGGAA-3 PRE elements (Figure 1A) [67-69], and IHC stains confirmed increased EHF abundance in PP skin, particularly within cell nuclei and the basal epidermis (Additional file 10). AVEN is an anti-apoptotic uDBP that interacts with 5-TTTCCA/TGGAAA-3 PREs (Figure 1B) [70], and IHC stains revealed diffuse elevation of AVEN in both the psoriatic epidermis and dermis (Additional file 11).

### PREs interact with IRF1, ISGF3, NF-κB and TFs with helix-turn-helix/homeo (*MEOX2*, *EN1*, *NANOG*) or other all-alpha-helical/high-mobility group (*SOX5*, *SOX8*, *SOX6*) DNA-binding domains

**Figure 2:**
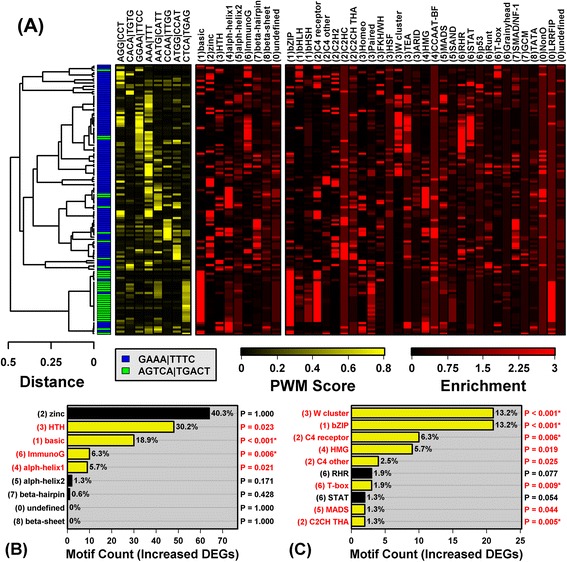
**PRE motifs significantly enriched in sequences upstream of psoriasis-increased DEGs.** We identified 126 PWM models matching motifs significantly enriched in sequences upstream of PP-increased DEGs (FDR < 0.10). **(A)** The 126 motifs were clustered based upon *k*-mer scores (HOPACH algorithm), leading to the identification of two motif sub-groups (blue and green, respectively). The shown *k*-mers were chosen by dividing the dendrogram into branches and identifying the 3- or 4-mer for which *k*-mer scores (yellow-black heatmap; see Methods) were most significantly elevated among motifs within a given branch. Red-black heatmaps show enrichment scores indicating how similar a PWM is to other PWMs associated with DNA-binding domain superfamily and class groups. To calculate such scores for a given motif, we first screened the 2935 PWMs to identify the 10% most similar “nearest neighbor” PWMs (Pearson correlation). We then assessed whether PWMs from a given superfamily or class were enriched among nearest neighbors (Fisher’s Exact Test), and the p-value from this test was used to calculate the enrichment score (−log_10_(p-value)). **(B)** Number of motifs associated with DNA-binding domain superfamilies. P-values assess whether motifs belonging to a superfamily are overrepresented among the 126 PRE motifs (right margin; Fisher’s Exact Test; asterisks denote FDR < 0.05). **(C)** Number of motifs associated with DNA-binding domain classes (top 10). P-values assess whether motifs from a given class are overrepresented among the 126 PRE motifs (right margin; Fisher’s Exact Test; asterisks denote FDR < 0.05).

**Figure 3:**
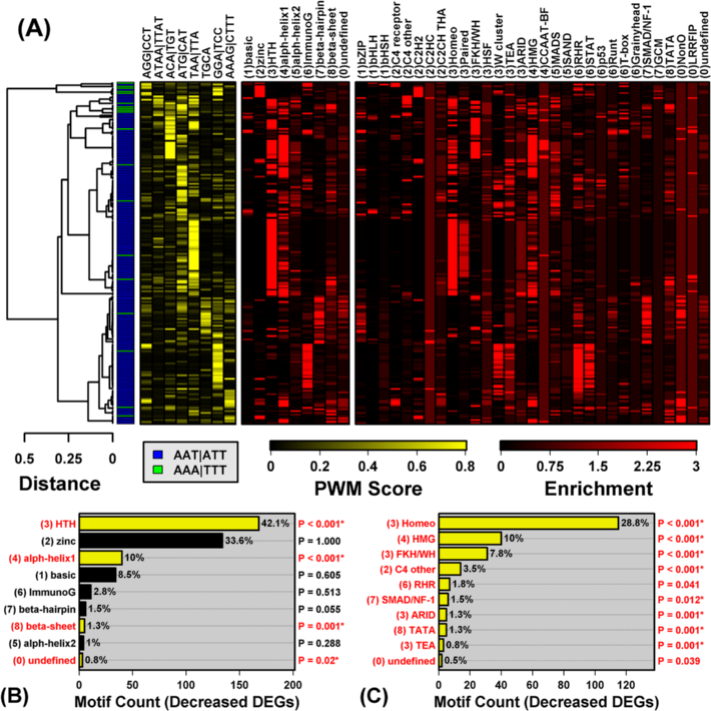
**PRE motifs significantly enriched in sequences upstream of psoriasis-decreased DEGs.** We identified 461 PWM models matching motifs significantly enriched in sequences upstream of PP-decreased DEGs (FDR < 0.10). **(A)** The 200 most significantly enriched motifs were clustered as described in Figure 2, leading to the identification of two motif sub-groups. The yellow-black heat map shows PWM *k*-mer scores (top margin). Red-black heatmaps show enrichment scores indicating how well PWMs match those associated with DNA-binding domain superfamily and class groups. **(B)** Number of motifs associated with DNA-binding domain superfamilies. P-values assess whether motifs belonging to a superfamily are overrepresented among the 461 PRE motifs (right margin; Fisher’s Exact Test; asterisks denote FDR < 0.05). **(C)** Number of motifs associated with DNA-binding domain classes (top 10). P-values assess whether motifs from a given class are overrepresented among the 461 PRE motifs (right margin; Fisher’s Exact Test; asterisks denote FDR < 0.05).

The spectrum of PREs revealed signatures of TF families that share particular DNA-binding domains (Figures 2 and 3). The 126 PREs associated with PP-increased DEGs were frequently recognized by IRF, ETS, Jun and Fos family TFs (Additional file 12, part A). Overall, the YY1 recognition site 5-ATGG/CCAT-3 was the most strongly enriched element in regions upstream of PP-increased DEGs (Additional file 13, part A). Cluster analysis of all 126 motifs identified two sub-groups, loosely characterized by the elements 5-AGTCA/TGACT-3 and 5-GAAA/TTTC-3, respectively (Figure 2). The first element partially matches the canonical AP-1 recognition sequence (5-TGANTCA-3), and accordingly, motifs from this group were associated with basic leucine zipper family TFs (e.g., AP-1 and RUNX1). The second element is strongly preferred by IRF1, the ISGF3 complex (STAT1, STAT2, IRF9) and, to a lesser degree, by NF-κB. Consistent with these trends, the 126 PREs were disproportionately associated with the helix-turn-helix, basic and immunoglobulin fold superfamilies, as well as the W cluster TF class (Figure 2).

Of 461 PRE motifs enriched in regions upstream of PP-decreased DEGs (FDR < 0.10), the top-ranked was recognized by a helix-turn-helix forkhead box TF (FOXP4) (consensus: 5-CTTTTCC/GGAAAAG-3) and most others also featured a TTTC core element (Additional file 13, part B). These elements partially match IRF1, ISGF3 and NF-κB recognition sequences. The set of 461 motifs was fairly homogenous, although two sub-groups could be discerned, with one set of 375 motifs enriched for 5-AAT/ATT-3 elements, and another set of 86 enriched for 5-AAA/TTT-3 elements (Figure 3). Enrichment for 5-AAT/ATT-3 elements was likely driven by two DNA-binding domain signature trends (Additional file 14). First, we discerned a distinct signature for TFs with the helix-turn-helix (homeo) DNA-binding domain (Figure 3). Consistent with this, several TFs with this domain interacted with PREs and were encoded by PP-decreased DEGs (i.e., *MEOX2*, *EN1*, *NANOG*, *CUX2*, *POU3F3*, *HOXB3*, *LHX2*, *HOXC9*; Figure 1A). Second, we identified a group of motifs interacting with TFs possessing an all-alpha-helical (high-mobility group) DNA-binding domain (Figure 3A), in agreement with down-regulation of SOX-related TFs in lesions (i.e., *SOX5*, *SOX8*, *SOX6*, *SOX10*; Figure 1A). Both of these binding domains (helix-turn-helix and other all-alpha-helical) were associated with PP-decreased TFs that preferred 5-AAT/ATT-3 elements (e.g., *MEOX2*, *EN1*, *LHX2*, *SOX5*, *SOX8*; Additional file 14).

There was only a slight correlation (*r*_s_ = 0.29) between motif enrichment scores obtained for PP-increased and PP-decreased DEGs (Additional file 13, part D). In part, such limited correspondence may be due to the AP-1 basic leucine zipper family signature, which was prominent with respect to PP-increased DEGs (Figure 2), but absent with respect to PP-decreased DEGs (Figure 3). Among 462 motifs enriched in sequences upstream of all DEGs (increased + decreased), trends were similar to those for PP-decreased DEGs (Figure 3), with clear signatures for TFs with helix-turn-helix/homeo and alpha-helical/high-mobility group DNA-binding domains (Additional file 15).

### PREs are prominent within enhancer regions of cytokine-encoding gene promoters (*IL17A*, *IL19* and *IL1B*)

Cytokines activate inflammatory and proliferative cascades in psoriasis lesions [71], as evidenced by the effectiveness of treatments directed against TNF, IL-17A and IL-23 [24,72]. We therefore considered whether PREs may contribute to regulation of cytokine gene expression.

**Figure 4:**
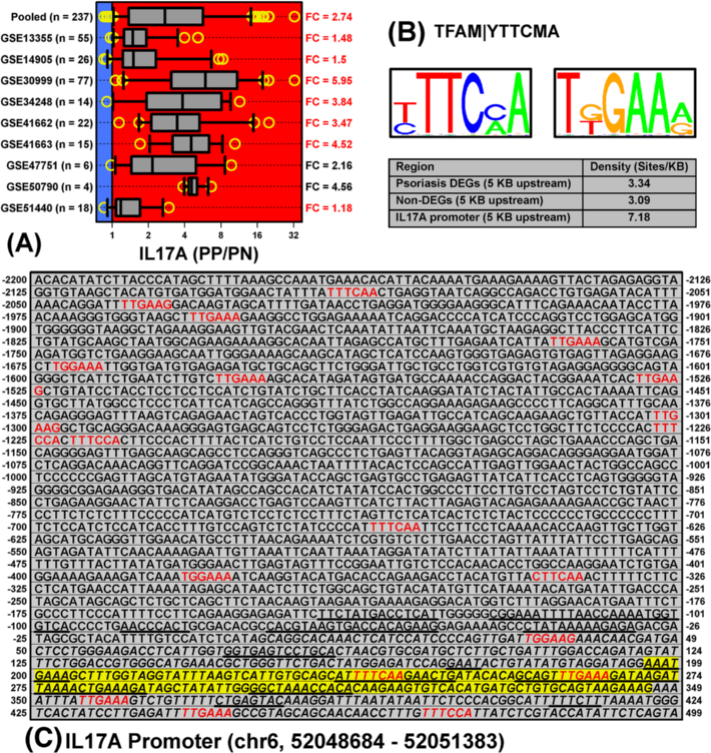
**PRE motifs are prominent in the** ***IL17A*** **promoter and present within an enhancer downstream from the transcription start site. (A)** *IL17A* expression is significantly elevated in psoriasis lesions. Grey boxes outline the middle 50% of fold-change (FC) estimates for each dataset (whiskers: middle 90%; yellow symbols: extreme values). The median FC for each dataset is listed (right margin; FDR < 0.05 for red labels). **(B)** Sequence logos for the TFAM PRE motif significantly overrepresented in sequence regions upstream of psoriasis DEGs. The motif’s frequency is elevated within the IL17A promoter (see table). **(C)** *IL17A* promoter (chr6, 52048984 – 52051683). TFAM motif matches (red font) and conserved elements are indicated (underlined sequence, phastcons ≥ 0.50). Yellow highlighted sequence denotes a DNase I hypersensitive site (Th17 cells).

Within psoriasis lesions, IL-17A is thought to be produced by Th17 cells, γδ T-cells, neutrophils, mast cells, and innate lymphoid cells [73]. Consistent with this, *IL17A* expression was significantly elevated in psoriasis lesions (FC = 2.74; *n* = 237 patients; Figure 4A). We inspected the *IL17A* promoter and noted high frequency of 5-TGGAAA/TTTCCA-3 elements. Such elements matched a motif associated with the all-alpha-helical (high mobility group) transcription factor A (TFAM). The motif was significantly enriched in sequences upstream of PP-increased DEGs (P = 5.2 × 10^−4^), PP-decreased DEGs (P = 8.8 × 10^−13^), and the full set of PP-increased and PP-decreased DEGs (P = 3.6 × 10^−14^). *TFAM* mRNA was not differentially expressed in psoriasis lesions (FC = 0.98, P = 0.366), but the TFAM motif contained elements similar to those present in IRF1, ISGF3 and NF-κB recognition sites (Figure 4B). Frequency of this motif was more than two-fold elevated in the *IL17A* promoter (Figure 4B). We could identify 19 such elements immediately surrounding the *IL17A* TSS (−2200 to 200 bp), but ENCODE data allowed us to pinpoint a Th17 cell DNase I hypersensitive site 200 – 350 bp downstream of the TSS (Figure 4C). Within this region, there were two TFAM recognition sites, both of which are conserved among mammalian species (i.e., phastcons ≥ 0.50; Figure 4C).

*IL19* is produced exclusively by KCs within lesions and recent work has shown that IL-19 can potentiate effects of IL-17A [74,75]. It was also reported that expression of *IL19* is more strongly elevated in psoriasis lesions than any other cytokine [75], and in agreement our data showed that *IL19* mRNA was elevated 5-fold in lesions (Additional file 16, part A; *n* = 237 patients). The *IL19* promoter featured increased frequency of a PRE motif recognized by nucleobindin 1 (NUCB1) (consensus: 5-ATGGGAA/TTCCCAT-3), which we found to be significantly enriched in regions upstream of PP-increased DEGs (P = 7.7 × 10^−5^), PP-decreased DEGs (P = 5.1 × 10^−4^), and the combined set of PP-increased and PP-decreased DEGs (P = 5.29 × 10^−8^) (Additional file 16, part B). We identified matches to this motif at eight loci 3600 – 4700 base pairs upstream from the *IL19* TSS (Additional file 16, part C). This region featured an NHEK histone modification associated with transcriptional activation and repression (histone H4 Lys 20 methylation, H4k20me1) [76]. Two NUCB1 motifs within this methylated element are conserved among mammals (chr1, 206968062–206968088; Additional file 16, part C).

IL-1 facilitates T-cell infiltration, blocks insulin-dependent KC differentiation and promotes KC proliferation [77,78]. *IL1B* expression was significantly elevated in psoriasis lesions (FC = 2.74; *n* = 237 patients; Additional file 17, part A). Within the *IL1B* promoter, there was increased frequency of a PRE motif recognized by TAL1 (consensus: 5-TTATCT/AGATAA-3), which was among the motifs most strongly enriched in promoter regions of PP-increased DEGs (P = 7.0 × 10^−3^), PP-decreased DEGs (P = 1.5 × 10^−9^), and the combined set of PP-increased and PP-decreased DEGs (P = 4.7 × 10^−10^). Density of this motif was elevated 4-fold in the *IL1B* promoter (Additional file 17, part B). We identified two such motifs within a candidate NHEK regulatory region upstream of the *IL1B* TSS, with one motif overlapping a conserved element (Additional file 17, part C). Combinatorial analysis of chromatin marks indicated that this region is an NHEK enhancer [66], and an open chromatin structure in NHEK was confirmed by independent DNase I hypersensitivity and Faire-seq data (Additional file 17, part C).

### Design of a complex decoy oligonucleotide (cdODN) directed against TFs activated in psoriasis lesions (FOXM1, ISGF3, IRF1 and NF-κB)

Complex decoy ODNs (cdODNs) with *cis*-regulatory elements recognized by multiple TFs can be used to block several disease-associated pathways concomitantly [79]. To design a candidate cdODN for psoriasis treatment, we focused on a limited set of TFs (FOXM1, IRF1 and NF-κB) as well as the IFN-stimulated gene factor 3 (ISGF3) complex (i.e., STAT1, STAT2 and IRF9). These TFs were considered because (i) they are encoded by PP-increased DEGs and (ii) they interact with PRE motifs enriched in sequences upstream of PP-increased DEGs (Figure 1). Prior work also supports these TFs as participants within the combined set of proliferative and inflammatory mechanisms driving lesion development [10,56,80,81].

**Figure 5:**
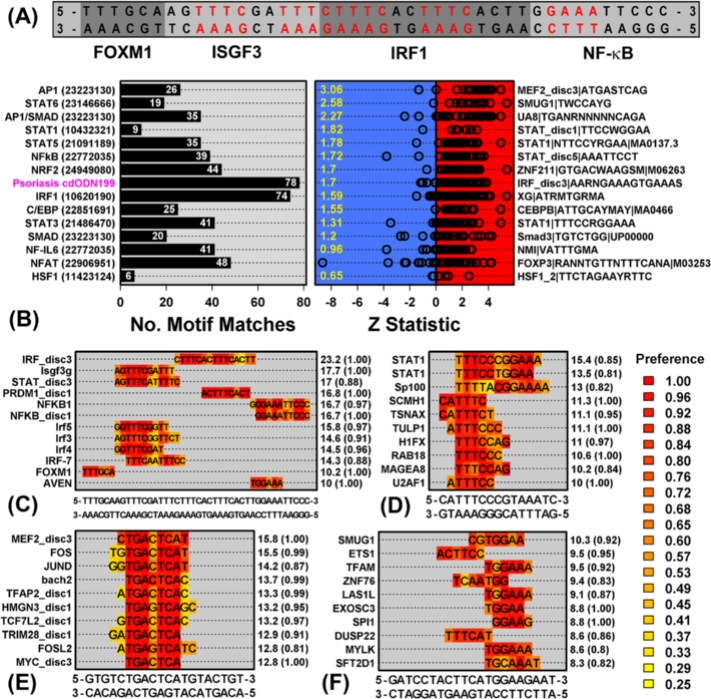
**Design of a complex decoy oligonucleotide (cdODN) directed against TFs activated in psoriasis lesions (FOXM1, ISGF3, IRF1 and NF-κB). (A)** Psoriasis cdODN199. The proposed design consists of consensus sequences from four PRE motifs significantly enriched in sequences upstream of PP-increased DEGs (FDR < 0.10). cdODN199 was chosen from among 384 possible combinations of the four PRE sequences (see text). 5-TTTC/GAAA-3 elements are shown with red font. **(B)** Top-ranked dODN molecules most closely matching PWM motifs enriched in sequences upstream of PP-increased DEGs. Each dODN was evaluated to identify matching motifs within our dictionary (2935 PWMs; *ψ*/*ψ*_max_ > 0.80, see Methods). The number of PWM matches is indicated (left) along with *Z* statistics (right), reflecting enrichment of matching motifs in sequences upstream of PP-increased DEGs. The PWM most closely matching each dODN is listed (right margin). Further details for each dODN can be obtained from the PubMed ID (left margin in parentheses). Parts **(C)** – **(F)** show the top 10 PWM models most closely matching **(C)** cdODN199, **(D)** STAT3 (15592573), (E) AP1 (23223130) and **(F)** STAT6 (23146666). dODN sequences are listed (bottom margin) and each figure shows consensus sequences for best-matching PWMs. Match scores are listed in the right margin (see Methods, Equation 2). The [0, 1] probability preference (PPM value) for each base is indicated by the color scale (right).

We identified top-ranking PRE motifs recognized by FOXM1, ISGF3, IRF1 and NF-κB, respectively. Given these four motifs, we enumerated 384 possible cdODN designs, based upon two 5′ to 3′ orientations for each site and alternative orderings within the cdODN. These designs varied in their specificity, since for any one cdODN we identified between 66 and 121 matches to the 2935 PWMs. For PWMs matching each cdODN design, we calculated an average enrichment score with respect to PP-increased DEGs (i.e., average *Z* statistic), and identified two designs for which this score was highest (designated “cdODN186” and “cdODN199”, respectively). The average *Z* statistic was similar for both designs (1.71 vs. 1.70), but cdODN199 was more specific, since it matched only 78 PWMs (as compared to 108 for cdODN186). cdODN199 was therefore examined further (Figure 5A). Notably, this design featured five of the 5-GAAA/TTTC-3 elements prominent within the *IL17A* promoter (Figure 4).

We compared cdODN199 to a set of 91 TF decoy molecules developed and validated in previous studies (Additional file 18). Surprisingly, most dODN designs were non-specific, often most closely matching a PWM associated with an off-target TF (Figure 5B). We identified 7 dODNs for which matching PWMs were associated with average *Z* statistics greater than cdODN199 (Figure 5B). Most of these, however, best matched an off-target TF, or were designed to block AP-1 activity, and may thus be expected to exacerbate lesion development rather than counteract it (Figures 5B, E and F) [82]. Despite the length of cdODN199 (42 bp), the most closely matching PWMs were associated with targeted TFs (i.e., IRF1, ISGF3 and NF-κB). Additionally, in combination, the IRF1 and NF-κB recognition sites create a binding site for AVEN (Figure 5C), an anti-apoptotic and PRE-associated uDBP with increased abundance throughout the psoriatic epidermis and dermis (Figure 1B and Additional file 11).

Although cdODN199 includes FOXM1, ISGF3, IRF1 and NF-κB recognition sites, it does not include a binding site for STAT3, previously validated as an effective dODN target in a psoriasis mouse model [17]. However, when we inspected the STAT3 decoy previously shown to resolve psoriasiform lesions in mice, we found that the decoy sequence most closely matched STAT1 PWMs (Figure 5D). Potentially, therefore, off-target inhibition of STAT1 or ISGF3 might have contributed to anti-psoriatic effects previously documented [17], and similar effects might be achieved using cdODN199, which includes an ISGF3 recognition sequence (Figure 5A).

### PRE motifs are disproportionately disrupted by SNP risk alleles at enhancer-associated non-coding psoriasis susceptibility loci

Genetic variants identified by psoriasis GWASs have been predominantly located in non-coding regions, suggesting that their influence on disease risk is indirect and could involve gene regulation [26]. We therefore asked whether psoriasis susceptibility variants disrupt or engender PREs within non-coding enhancers.

**Figure 6:**
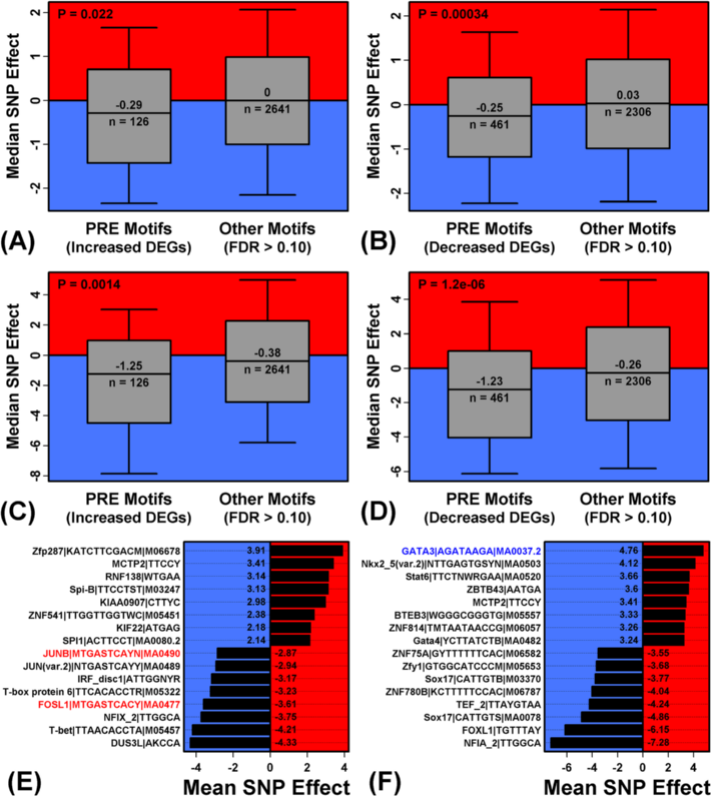
**PREs are disproportionately disrupted by SNP risk alleles at enhancer-associated non-coding psoriasis susceptibility loci.** We analyzed 572 psoriasis-associated SNPs, including **(A, B)** 324 non-coding SNPs and **(C, D)** 53 non-coding SNPs within an NHEK enhancer. Risk alleles for these SNPs were evaluated to assess whether they strengthened (effect > 0) or weakened (effect < 0) matches to the 2935 motifs included in our dictionary (see Methods, Equation 3). Parts **(A)** – **(D)** compare the median SNP effect between PRE motifs enriched in sequences upstream of psoriasis DEGs (FDR < 0.10) and all other non-enriched motifs (FDR > 0.10). For each motif group, boxes outline the middle 50% of risk allele effects and whiskers outline the middle 80% of effects (324 SNPs in parts **A** and **B**; 53 SNPs in parts **C** and **D)**. P-values assess whether the median risk allele effect differs between motif groups (Wilcoxon rank sum test). Part **(E)** lists PREs associated with PP-increased DEGs (FDR < 0.10) with the lowest and highest effects (on average among the 324 non-coding SNPs). Part **(F)** lists PREs associated with PP-decreased DEGs (FDR < 0.10) with the lowest and highest effects (on average among the 324 non-coding SNPs).

We identified 536 SNPs in strong linkage disequilibrium (*r*^2^ > 0.90) with 36 lead SNPs from a psoriasis GWAS meta-analysis [12], yielding a total of 572 SNPs (lead + linked SNPs combined). Of these 572 SNPs, 324 were non-coding, while 53 were both non-coding and within an NHEK enhancer. We screened the 2935 PWMs and calculated the average difference in binding affinity with respect to risk and non-risk alleles (Figure 6). The 126 PRE motifs enriched in regions upstream of PP-increased DEGs (FDR < 0.10) were more likely to be disrupted by risk alleles, as compared to all other 2641 motifs (FDR > 0.10) (P = 0.022 for non-coding SNPs; P = 0.0014 for non-coding enhancer-associated SNPs; Figures 6A and C). To an even greater degree, the 461 PRE motifs enriched in sequences upstream of PP-decreased DEGs (FDR < 0.10) were more likely to be disrupted by risk alleles, when compared to the other 2306 motifs (FDR > 0.10) (P = 0.00034 and P = 1.2 × 10^−6^; Figure 6B and D). Psoriasis risk alleles at non-coding SNPs therefore tend to abrogate, rather than engender, PRE motifs. PREs most frequently disrupted by risk alleles were recognized by AP-1 (Figure 6E), while PREs most commonly engendered by risk alleles were recognized by GATA3 (Figure 6F).

**Figure 7:**
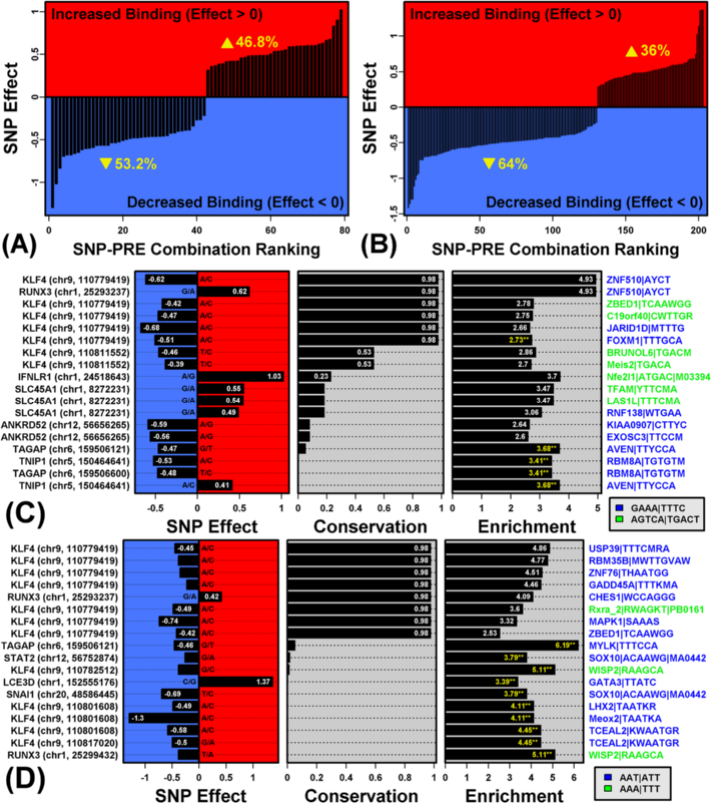
**Identification of enhancer-associated non-coding psoriasis susceptibility loci as potential sites of allele-specific transcription factor binding.** We examined 53 psoriasis-associated non-coding SNPs within an NHEK enhancer to identify SNP-PRE combinations representing possible sites of allele-specific TF binding. **(A)** Predicted effects of risk alleles with respect to 79 SNP-PRE combinations involving PRE motifs enriched in sequences upstream of PP-increased DEGs (FDR < 0.10) (see Methods, Equation 3). **(B)** Predicted effects of risk alleles with respect to 203 SNP-PRE combinations involving PRE motifs enriched in sequences upstream of PP-decreased DEGs (FDR < 0.10). **(C)** The 79 SNP-PRE combinations from **(A)** were filtered to identify those for which the SNP locus is conserved and/or the PRE is recognized by a PP-increased DEG. **(D)** The 203 SNP-PRE combinations from **(B)** were filtered to identify those for which the SNP locus is conserved and/or the PRE is recognized by a PP-decreased DEG. In both **(C)** and **(D)**, we list the SNP’s genomic location and nearest gene (left margin) and the PRE motif label for the corresponding PWM matrix (right margin). The phastcons conservation score for the SNP locus is also shown, along with *Z* statistic indicating how strongly the PWM motif was enriched in sequences upstream of **(C)** PP-increased DEGs or **(D)** PP-decreased DEGs. The Z statistic is listed within the bar graphs, with yellow text denoting cases in which the PWM is recognized by **(C)** a PP-increased DEG or **(D)** a PP-decreased DEG.

We screened 6678 SNP-PRE combinations involving one of the 53 non-coding enhancer-associated SNPs and one of the 126 PRE motifs enriched in sequences upstream of PP-increased DEGs. Of these, there were 79 cases (1.18%) in which the SNP risk variant engendered (37 cases; 0.554%) or disrupted (42 cases; 0.629%) a PRE match (Figure 7A). These percentages and the disrupted/engendered proportion (1.13) did not differ significantly from values observed in simulation trials, in which effects of randomly sampled SNPs on PRE matches were identically quantified (P ≥ 0.34; Additional file 19). We next screened 24433 SNP-PRE combinations involving one of the 53 non-coding enhancer-associated SNPs and one of the 461 PRE motifs enriched in sequences upstream of PP-decreased DEGs. Of these, there were 203 cases (0.83%) in which the SNP risk variant engendered (73 cases; 0.298%) or disrupted (130 cases; 0.532%) a PRE match (Figure 7B). These percentages differed slightly from those observed in simulation trials (P ≤ 0.162), while the disrupted/engendered proportion (1.79) was significantly large (P = 0.045; Additional file 19, part F). This again suggested that psoriasis risk alleles are more likely to disrupt, rather than engender, PRE motifs, particularly those enriched in sequences upstream of PP-decreased DEGs.

We next aimed to identify individual SNP-PRE combinations most likely to be associated with allele-specific TF/uDBP binding (Figure 7). The 79 and 203 SNP-PRE combinations cited above (Figure 7A and B) were filtered to identify those for which the SNP locus is conserved and/or the PRE is recognized by a TF/uDBP-encoding DEG (Figure 7C and D). This highlighted SNP-PRE pairs involving PREs recognized by TFs or uDBPs with increased expression in psoriasis lesions (i.e., *AVEN*, *RBM8A* and *FOXM1*; Figure 7C). For PP-decreased DEGs, nearly all (16/18) of the filtered SNP-PRE combinations involved PRE disruption by the risk allele (Figure 7D). Several of these PREs interacted with TFs/uDBPs encoded by PP-decreased mRNAs (i.e., *WISP2*, *TCEAL2*, *MEOX2*, *LHX2*, *SOX10*, *GATA3*, and *MYLK*; Figure 7D).

## Discussion

Psoriasis is debilitating for many patients with direct and indirect costs that exceed one billion dollars annually within the United States alone [3]. To identify TFs contributing to aberrant KC activity, including abnormal differentiation and excessive proliferation, we evaluated gene expression in psoriasis lesions from a meta-cohort of 237 patients. Through *in silico* screening of known DNA binding sites, our findings highlight proteins not yet well studied in psoriasis, including TFs (FOXM1, EHF, SOX5) and uDBPs (AVEN, RBM8A, GPAM, WISP2). We also uncovered “psoriasis response elements” (PREs) overrepresented in psoriasis DEG promoter regions, which are present within enhancers near cytokine-encoding genes (e.g., *IL17A*, *IL19* and *IL1B*). We show that PREs can be strategically combined to create a cdODN concomitantly targeting psoriasis-activated TFs (FOXM1, ISGF3, IRF1 and NF-κB), illustrating how transcriptome informatics can be directly connected to dODN development. Finally, our findings address the challenge of how to interpret GWAS hits within non-coding regions [26], and we have identified disease-associated SNPs within non-coding NHEK enhancers that disrupt or engender PRE motifs. As possible sites of allele-specific TF/uDBP binding, such SNPs represent priority candidates for functional studies. These findings offer new insights into the underlying transcriptional circuitry of psoriasis lesions, and demonstrate how sequence-specific TF/uDBP-DNA interactions can be exploited to support dODN drug development and enhance interpretation of non-coding GWAS signals.

**Figure 8:**
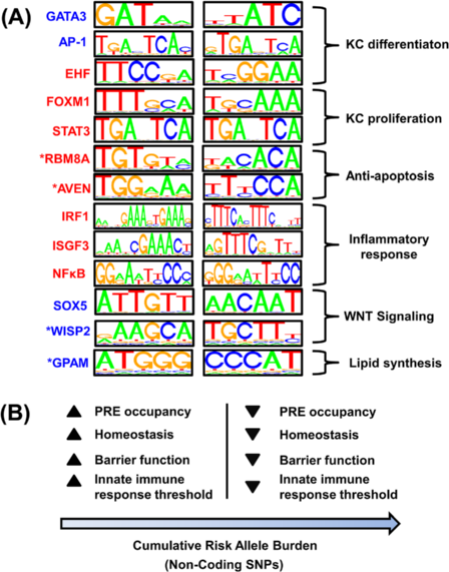
**Summary of differentially expressed TFs/uDBPs interacting with PREs and proposed model linking cumulative risk allele burden to PRE occupancy. (A)** Selected TFs and uDBPs that are differentially expressed in psoriasis lesions and interact with PREs (red font: PP-increased DEGs; blue font: PP-decreased DEGs; asterisks: uDBPs). Sequence logos depict DNA-binding affinities (both 5′-3′ orientations are shown). **(B)** Proposed model linking cumulative risk allele burden at non-coding SNPs to PRE occupancy and disease susceptibility. Risk alleles at disease-associated SNPs favor decreased PRE occupancy, thereby disrupting interactions between PREs and *trans*-acting factors (e.g., AP-1). This increases susceptibility by compromising epidermal homeostasis and barrier function, thus lowering the trigger threshold for innate immune responses to facilitate immune cell infiltration and lesion development.

Psoriasis lesions develop in response to interplay between lesion-infiltrating inflammatory cells and local KCs, which respond to cytokine signals by failing to differentiate completely and adopting a phenotype resembling that of proliferating basal-layer KCs [1,2]. This pathological KC activity proceeds in coordination with an underlying TF regulatory network. Previous studies have identified DEGs showing altered expression in psoriasis lesions, but many DEGs may play only a passive role in lesion development, without active participation in the disease process [18-24]. In our analyses, we first identified psoriasis DEGs, but then filtered these to define a more exclusive set of DEGs for which encoded proteins interact with PRE motifs (Figure 1). By combining information in this way, we narrowed the focus considerably, highlighting those DEGs with an extra layer of evidence for active participation in the psoriasis transcription network. In agreement with prior work, our findings lend support to AP-1, IRF1, NF-κB, STAT3, GATA3 and the ISGF3 complex (STAT1, STAT2 and IRF9) as “hubs” within this network (Figure 8A) [10,56,60]. Additionally, however, we uncovered TFs not extensively studied in psoriasis, but which may nonetheless have important roles in KC differentiation, KC proliferation, apoptosis, inflammation, WNT signaling and lipid synthesis (e.g., FOXM1 and EHF; Figure 8A) [67-69,80]. Our findings also suggest the possibility that repression of gene expression in lesions is driven, at least in part, by decreased abundance of TFs with helix-turn-helix (homeo) and other all-alpha-helical (high-mobility group) DNA-binding domains (i.e., MEOX2, EN1, NANOG, SOX5, SOX8, SOX6). Such TFs prefer 5-TAA/TTA-3 elements (overrepresented in promoters of psoriasis-decreased DEGs), and their decreased expression in psoriasis may contribute to incomplete KC differentiation, thereby favoring KC proliferation [83,84].

Unconventional DNA-binding proteins (uDBPs) participate in sequence-specific DNA interactions and cellular cytokine responses [33,43]. We identified two uDBPs encoded by PP-increased DEGs that recognize PRE motifs and have anti-apoptotic functions (*AVEN*, *RBM8A*). Within lesions, KCs from the basal layer are resistant to apoptosis [85-87], while those in the suprabasal differentiated epidermis appear susceptible [86], and this may alter the differentiation/proliferation balance maintaining homeostasis in normal skin. AVEN interferes with apoptosome assembly by interacting with the adaptor protein Apaf-1, but this activity requires proteolytic removal of the N-terminal domain [70]. The cleavage reaction is mediated by Cathepsin D (*CDSD*) [70], which also shows elevated expression in psoriasis lesions (FC = 1.56; P = 4.61 × 10^−38^). Expression of RNA-binding protein 8A (*RBM8A*) appears necessary to prevent apoptosis, since *RBM8A* deficiency triggers apoptosis and disrupts cell cycle progression [88,89]. Beyond this, RBM8A binds STAT3 to modulate its activity in cells stimulated by IL-6 or TNF [90,91]. Finally, expression of glycerol-3-phosphate acyltransferase (GPAM) was significantly decreased in psoriasis lesions and our analysis revealed that GPAM recognizes PRE motifs enriched in sequences upstream of PP-decreased DEGs (Figure 1). Since GPAM is required for triacylglycerol and phospholipid biosynthesis [92], decreased GPAM activity may contribute to defects in epidermal barrier and cornified envelope formation, which is hypothesized to be a factor triggering innate immune responses at initial stages of lesion development [93].

TF decoys have become an established approach for nucleic acid-based treatment of human disease and skin conditions [14-16]. We have here introduced a bioinformatic pipeline for data-driven cdODN design, in which we (i) screen binding sites of known TFs and uDBPs to identify *cis*-regulatory elements associated with a disease phenotype, (ii) select a small set of the enriched regulatory elements as cdODN “building blocks”, and (iii) enumerate and screen all possible cdODN conformations to select the one that best matches motifs overrepresented in promoters of disease-associated genes. Applying this approach, we designed a cdODN (cdODN199) targeting TFs whose activation in lesions is likely to augment KC proliferation and cytokine-trigged inflammatory cascades (i.e., FOXM1, ISGF3, IRF1 and NF-κB). We expect that, by testing the *in vivo* activity of cdODN199, it will be possible to introduce refinements, including the addition or removal of certain PRE elements. Our main innovation in the current study is development of a bioinformatic analysis protocol for designing a cdODN matched to the differential expression profile of psoriasis lesions. Computational screens of this type have not been previously used to ensure such a “lock-and-key” type relationship between cdODN sequence and disease phenotype. The importance of specificity is, however, clearly demonstrated by the clinical failure of Edifoligide, an E2F dODN developed to prevent neointimal hyperplasia in vein bypass grafts [94]. After many years and considerable development costs, Edifoligide was ineffective for its intended purpose, possibly because the dODN sequence was not sufficiently specific for the targeted E2F factor [94]. For most dODN molecules, such non-specificity may be the rule, rather than the exception, since we have shown that dODNs generally match PWMs associated with multiple TFs (Figure 5). By matching dODN sequence to the disease phenotype’s expression profile, however, we have outlined a computationally-driven approach for improving specificity. In particular, this provides a practical strategy for psoriasis and other skin diseases, since lesions can be readily sampled and analyzed by expression profiling.

GWAS findings have been instrumental for identifying the genes and pathways serving as genetic trigger points that predispose to psoriasis [12,20,93]. Similar to other complex diseases, however, most psoriasis GWAS signals have been identified in non-coding regions (intronic or intergenic), suggesting that their effects on gene regulation, rather than protein function, may explain their contribution to susceptibility [28,30,95]. This has challenged our interpretation of GWAS findings, in part because we lack a complete understanding of which sequence-specific TF-DNA or uDBP-DNA interactions coordinate plaque development. To bridge this gap, we characterized the core set of PRE *cis*-regulatory motifs enriched in psoriasis DEG promoters. This allowed us to identify SNPs at which risk alleles create or engender PREs recognized by DEG-associated TFs/uDBPs (e.g., *AVEN*, *RBM8A*, *FOXM1*, *WISP2*, *TCEAL2*, *MEOX2*, *LHX2*, *SOX10*, *GATA3* and *MYLK*; Figure 7C and D). Potentially, such SNPs may represent sites at which risk alleles have major impacts on TF/uDBP-PRE interaction, with important downstream consequences that predispose to psoriasis, or genetically-related autoimmune diseases [96].

An alternative model, however, is that an accumulation of risk alleles at non-coding loci, each with minor effects on TF/uDBP-PRE interaction, has an aggregate effect promoting susceptibility in those individuals with the greatest cumulative risk allele burden (Figure 8B). This latter view is consistent with an “analog” view of transcription [97], in which expression of genes ensuring homeostasis and normal epidermal barrier function gradually increases in proportion to noncooperative PRE-TF/uDBP interactions in key genome regions. Supporting this idea, risk alleles tended to decrease match scores between PRE motifs and genomic loci, often to a limited degree but nonetheless consistently across non-coding psoriasis-associated SNPs (Figure 6A – 6D). A consistent effect of non-coding risk alleles, moreover, was to degrade matches to PREs recognized by TFs supporting normal barrier function and KC differentiation (e.g., AP-1; Figure 6E). Such a pattern may be driven by haplotypes of linked non-coding risk alleles, where each individual allele may have only a minor effect on PRE occupancy at a given locus. Cumulatively, however, such minor effects may engender disease-associated haplotypes that contribute to population-level variation in PRE occupancy (e.g., by AP-1), which is in turn connected to susceptibility through its influence on the expression of genes promoting normal KC differentiation and barrier function (Figure 8B). Such effects may parallel those of some coding variants (e.g., *TRAF3IP2* and/or *TNFAIP3*), which may not increase risk by amplifying inflammatory responses directly, but instead increase risk by disrupting epidermal homeostasis under non-inflammatory conditions, thereby lowering immune response thresholds [98,99].

Cellular function depends upon a dynamic protein-DNA interactome, where disease states may correspond to aberrant connections or missing links within this network [100]. To better understand such network abnormalities, *in silico* screening of TF/uDBP binding sites offers a valuable approach, and we have shown that this can facilitate discovery of *cis*-regulatory modules, design of targeted dODN therapies, and interpretation of GWAS hits at non-coding loci. In coming years, this informatics strategy can be applied on a larger scale, as we develop a more complete empirical database of DNA sequence preferences for human TFs and uDBPs. We were, for instance, able to identify 447 known TFs for which no known binding site model is available in an existing database [43,45-49]. Our understanding of TF-DNA interactions may therefore be, at best, 70% complete, notwithstanding that many TFs have context-specific binding affinities dependent upon co-factors, cell type, cellular activation status, and/or genetic background [101,102]. Beyond this, we have only a partial catalogue of uDBP recognition sites, and although we now have foundational *in vitro* chromatin feature data for key cell types, the *in vivo* relevance of these features and their consistency across genetic backgrounds is not fully established [103]. Addressing these gaps will require continued systematic data aggregation with complementary development of statistical methods, such as improved approaches for modeling TF sequence specificity [104]. Despite these challenges, targeted analysis of the protein-DNA interactome can guide hypothesis-driven studies of human disease, while illuminating a data-driven pathway towards development of nucleic acid-based therapies.

## Conclusions

The psoriasis transcriptome points towards previously unknown “psoriasis response elements” (PREs) enriched in DEG upstream sequences. We show that PREs are located within TSS-proximal regulatory regions near key cytokine genes (e.g., *IL17A*, *IL19* and *IL1B*). Although 106 TFs are encoded by psoriasis DEGs, only a fraction interacts with PREs (26/106), and several of these have not yet been examined in psoriasis studies (e.g., *FOXM1*, *EHF*, *SOX5*). Similarly, we identified DEG-encoded uDBPs that interact with PREs, whose function in psoriasis is presently unknown (e.g., *AVEN*, *RBM8A, GPAM*, *WISP2*). Having identified diverse PRE motifs, we demonstrate two applications for this information, including (i) informatics-guided design of cdODN molecules with a lock-and-key relationship to the disease phenotype expression profile and (ii) identification of non-coding enhancer-associated SNPs that disrupt/engender PREs (i.e., allele-specific TF/uDBP binding). Our findings illustrate the strong potential of our *in silico* strategy with respect to both applications. These results can help guide development of psoriasis therapies, including first-line treatments for mild-to-moderate psoriasis and adjuvant medications for immunosuppressive therapy. We envision that data resources and the informatics pipeline developed here can be extended to other complex genetic diseases, as a general strategy to facilitate dODN design and enhance interpretation of GWAS findings.

## Additional files

Additional file 1:
**Quality control processing of lesional (PP) and uninvolved (PN) skin microarray samples.** (A) PP/PN fold-change comparison (PP/PN) between GSE51440 (HT HG-U133+ PM array plates) and datasets generated using Affymetrix Human Genome U133 Plus 2.0 arrays. Yellow ellipses outline the middle 50% of FC estimates (Mahalanobis distance). (B – I) QC metrics. We calculated (B) average background, (C) scale factor, (D) percentage of probe sets called present, (E) degradation scores, (F) NUSE median, (G) NUSE IQR, (H) RLE median and (I) RLE IQR. Yellow symbols denote excluded samples (*Z* scores > 3.5 in absolute value). (J) Median FC estimates among PP-increased (FC > 2; FDR < 0.05) and PP-decreased DEGs (FC < 0.50; FDR > 0.05). Two excluded outlier samples are indicated. (K) Principal component plot for GSE51440 samples (HT HG-U133+ PM array plates). (L) Principal component plot for all other samples (Affymetrix Human Genome U133 Plus 2.0 arrays). (M) Final cluster analysis of the 237 paired PP and PN samples, with distance between samples based upon PP – PN differences in RMA expression scores (i.e., Euclidean distance normalized to [0,1] interval). Additional file 2:
**Construction of motif dictionary by integration across seven sources. The initial set of 4378 motifs was filtered to remove redundant motifs and motifs with low information content, yielding the final set of 2935 motifs used in our analyses (see** Methods **).** The table lists the number of motifs obtained from each source before and after filtering. The number of unique human genes associated with motifs is listed in parentheses. Additional file 3:
**Gene ontology (GO) biological process (BP) terms and genes associated with DNA motifs within our dictionary.** The 2935 PWM motifs were associated with 1422 unique human genes. The Venn diagram shows the number of these genes associated with GO biological process terms “transcription factor activity” (GO:0003700), “transcription cofactor activity” (GO:0003712) and “DNA binding” (GO:0003677). Additional file 4:
**Transcription factor DNA-binding domain superfamily and class groups. 1509 human TF-encoding genes from the TFclass database were assigned to superfamily and class groups based upon their DNA-binding domain.** We identified the largest superfamily and class groups and determined the number of genes in each group associated with at least one PWM model from our dictionary of 2935 motifs (red). Additional file 5:
**Cluster analysis of the 2935 PWM models included within our motif dictionary.** Motifs were clustered as described in Figures 2A and 3A. The yellow-black heatmap shows motif *k*-mer scores (top margin). Red-black heatmaps show enrichment scores indicating how well a given PWM matches other PWMs associated with different DNA-binding domain superfamily and class groups (TFclass database). Additional file 6:
**Differential expression statistics for 16117 skin-expressed genes. This file provides differential expression statistics for the 16117 skin-expressed genes included in our analysis (PP versus PN skin;** ***n*** **= 237 patients).**
Additional file 7:
**KC proliferation and differentiation markers in psoriasis lesions and uninvolved skin (** ***n*** **= 237 patients).** (A) KC proliferation and differentiation markers (left margin). The number of patients showing increased (red) or decreased (blue) expression is indicated for each gene, along with the median PP/PN fold-change and p-value (right margin; Wilcoxon rank sum test). (B – F) Distribution of FC estimates across all patients for selected genes. Additional file 8:
**Hallmark psoriasis genes with near-universally increased expression in lesional skin (** ***PI3,*** ***IL36G,*** ***KYNU,*** ***SERPINB13*** **and** ***WNT5A*** **). We identified five genes for which expression was higher in lesional (PP) as compared to uninvolved skin (PN) for all patients (** ***n*** **= 237).** (A) Distribution of PP/PN fold-change (FC) estimates among patients (grey boxes: middle 50%; yellow boxes: middle 80%). Median FC estimates and p-values are listed (right margin). (B) Mean expression in lesional (PP) and normal skin (NN) from control subjects (RNA-seq, GSE54456). Expression is measured using fragments per kilobase of transcript per million mapped reads (FPKM). (C) Cytokine responses in cultured KCs (*HaCAT KCs; **reconstituted epidermis). The cytokine, concentration (per μL), duration of cytokine treatment, and Gene Expression Omnibus series identifier is listed for each experiment (top margin). (D) Skin disease panel. The expression of each gene was evaluated in other skin diseases and compared to its expression in normal skin. Additional file 9:
**TF-encoding DEGs are more likely to interact with PRE motifs than TF-encoding non-DEGs.** Our analysis identified 1149 TF-encoding genes expressed in human skin, including 39 PP-increased DEGs, 67 PP-decreased DEGs, and 1043 non-DEGs with similar expression in lesional and uninvolved skin. We evaluated whether TF-encoding DEGs are more likely to interact with PRE motifs than TF-encoding non-DEGs. The analysis was performed with respect to TF-encoding PP-increased DEGs (*n* = 39), PP-decreased DEGs (*n* = 67) and both PP-increased + PP-decreased DEGs (*n* = 106); additionally, analyses were performed with respect to PRE motifs enriched upstream of PP-increased DEGs (*n* = 126 PREs), PP-decreased DEGs (*n* = 461), and the combined set of all DEGs (*n* = 462). For each row of the table, the percentage of TF-encoding DEGs associated with a PRE motif was compared with that observed among TF-encoding non-DEGs (Fisher’s Exact Test). Additional file 10:
**TFs encoded by psoriasis DEGs that interact with PREs. (A) Expression in psoriasis lesions and normal skin from control subjects (RNA-seq; GSE54456).** Symbols denote average expression (±1 standard deviation). Expression is measured using fragments per kilobase of transcript per million mapped reads (FPKM). (B) Expression in blood from psoriasis patients and control subjects (GSE55201). In (A) and (B), genes in red and blue font have increased and decreased expression in PP vs. PN skin, respectively (*n* = 237 patients, microarray). (C) IHC stain for ETS homologous Factor (EHF) in PP skin (10X magnification). (D) IHC stain for EHF in PN skin (10X magnification). Additional file 11:
**uDBPs encoded by psoriasis DEGs that interact with PREs. (A) Expression in psoriasis lesions and normal skin from control subjects (RNA-seq; GSE54456).** Symbols denote average expression (±1 standard deviation). Expression is measured using fragments per kilobase of transcript per million mapped reads (FPKM). (B) Expression in blood from psoriasis patients and control subjects (GSE55201). In (A) and (B), genes in red and blue font have increased and decreased expression in PP vs. PN skin, respectively (*n* = 237 patients, microarray). (C) IHC stain for apoptosis caspase activation inhibitor (AVEN) in PP skin (10X magnification). (D) IHC stain for AVEN in PN skin (10X magnification). Additional file 12:
**DNA-binding domain families associated with psoriasis DEGs. DNA-binding domain families most strongly overrepresented among PRE motifs enriched in sequences upstream of (A) PP-increased DEGs, (B) PP-decreased DEGs, (C) PP-increased and PP–decreased DEGs.** P-values assess whether motifs belonging to a family are significantly overrepresented among the set of PRE motifs associated with (A) – (C), respectively (right margin; Fisher’s Exact Test). Example sequence logos are shown for the most strongly overrepresented TF families (i.e., interferon regulatory factors, part A; NK-related factors, part B; HOX-related factors, part C). Additional file 13:
**Psoriasis response elements (PREs) most strongly enriched in genomic sequences upstream of psoriasis DEGs.** We screened 2935 binding sites (PWM matrix models) to identify PRE motifs most significantly enriched in 5KB regions upstream of psoriasis DEGs. (A – C) Top 12 motifs enriched with respect to (A) PP-increased DEGs, (B) PP-decreased DEGs, and (C) all psoriasis DEGs, respectively. For each motif, enrichment is proportional to the *Z* statistic obtained from semiparametric generalized additive logistic modeling (see Methods). The ratio between the number of motif occurrences in regions upstream of psoriasis DEGs and the number of occurrences among other skin-expressed genes is listed (Ratio). Labels in red or blue font (left margin) denote cases in which the motif is recognized by a protein encoded by a PP-increased DEG or PP-decreased DEG, respectively. (D) Comparison between enrichment *Z* statistics obtained with respect to PP-increased DEGs and PP-decreased DEGs (*n* = 2935 PWM models). The yellow circle outlines the 50% of values closest to the centroid (Mahalanobis distance). (E) PWM sequence logos associated with top-ranking motifs from (A) – (C). Additional file 14:
**PREs upstream of PP-decreased DEGs interact with helix-turn-helix (homeo) and other all-alpha-helical (high-mobility group) DNA-binding domains.** (A) TFs encoded by helix-turn-helix (homeo) and other all-alpha-helical (high-mobility group) DNA-binding domains show decreased expression in psoriasis (*n* = 237 patients; microarray). Most PP-decreased TFs with these domains recognize DNA elements enriched in sequences upstream of PP-increased and/or PP-decreased DEGs (see *Z* statistics; middle two figures). The right-most figure shows relative expression in dermis, suprabasal epidermis and basal epidermis (laser capture microdissection, GSE42114). (B) Mean expression in lesional (PP) and normal skin (NN) from control subjects (RNA-seq; GSE54456). Expression is measured using fragments per kilobase of transcript per million mapped reads (FPKM). (C) Sequence logos for helix-turn-helix (homeo) TFs. (D) Sequence logos for other all-alpha-helical (high-mobility group) TFs. Additional file 15:
**PRE motifs significantly enriched in sequences upstream of psoriasis-increased and psoriasis-decreased DEGs.** We identified 462 PWM models matching motifs significantly enriched in sequences upstream of PP-increased and PP-decreased DEGs (FDR < 0.10). (A) The 200 most significantly enriched motifs were clustered as described in Figure 2, leading to the identification of three motif sub-groups. The yellow-black heat map shows *k*-mer scores for each motif (top margin). Red-black heatmaps show enrichment scores indicating how well a given PWM matches others associated with DNA-binding domain superfamily and class groups (TFclass database). Additional file 16:
**PRE motifs are prominent in the** ***IL19*** **promoter and present within an upstream enhancer region.** (A) *IL19* expression is significantly elevated in psoriasis lesions. Grey boxes outline the middle 50% of fold-change (FC) estimates for each dataset (whiskers: middle 90%; yellow symbols: extreme values). The median FC for each dataset is listed (right margin; FDR < 0.05 for red labels). (B) Sequence logos for the NUCB1 motif significantly overrepresented in sequence regions upstream of psoriasis DEGs. The motif’s frequency is elevated within the *IL19* promoter (see table). (C) *IL19* promoter (chr1, 206967214–206969913). NUCB1 motif matches (red font) and conserved elements are indicated (underlined, phastcons ≥ 0.50). Yellow highlighted sequence denotes H4k20me1 histone modification (NHEKs). Additional file 17:
**PRE motifs are prominent in the** ***IL1B*** **promoter and present within an upstream enhancer region.** (A) *IL1B* expression is significantly elevated in psoriasis lesions. Grey boxes outline the middle 50% of fold-change (FC) estimates for each dataset (whiskers: middle 90%; yellow symbols: extreme values). The median FC for each dataset is listed (right margin; FDR < 0.05 for red labels). (B) Sequence logos for the TAL1 motif significantly overrepresented in sequence regions upstream of psoriasis DEGs. The motif’s frequency is elevated within the *IL1B* promoter (see table). (C) *IL1B* promoter (chr2, 113594157–113596856). TAL1 motif matches (red font) and conserved elements are indicated (underlined, phastcons ≥ 0.50). Yellow highlighted sequence denotes a DNase I hypersensitive site and Faire-seq peak (NHEKs). Additional file 18:
**List of 91 unique TF decoy oligonucleotides (dODNs).** A literature review identified 167 dODNs used and validated in prior studies. These were screened to exclude redundant dODNs with the same sequence, yielding a set of 91 unique dODNs. For those dODNs reported by multiple publications, the table in this file lists the earliest publication reporting the dODN sequence. Additional file 19:
**Variants at psoriasis-associated non-coding/enhancer SNP loci disproportionately disrupt PRE motifs in sequences upstream of PP-decreased DEGs (simulation analysis).** Simulation was used to assess the effects of risk variants at psoriasis-associated SNP loci on PRE motifs in comparison to the effects of genetic variants at randomly sampled SNP loci (1000 trials). We considered 53 psoriasis-associated non-coding SNPs within NHEK enhancers and the effects of associated risk variants on PRE motif matches. In each simulation trial, 53 SNPs were randomly chosen from a larger pool of 1.82 million SNPs, which was generated by identifying autosomal SNPs positioned within non-coding NHEK enhancer regions and located at least 500 kb from any psoriasis-associated SNP (at least 4 Mb in the MHC region). The 53 random SNPs were frequency-matched with respect to the 53 psoriasis-associated SNPs. For each random SNP, one associated genetic variant was randomly designated as the risk allele. Analyses were performed with respect to the 126 PRE motifs enriched in sequences upstream of PP-increased DEGs (parts A – C), as well as the 461 PRE motifs enriched in sequences upstream of PP-decreased DEGs (parts D – F). In each trial, we evaluated the percentage of SNP-PRE combinations in which a PRE match was disrupted (parts A and D) or engendered (parts B and E), as well as the ratio of disrupted to engendered matches (parts C and F). Figures show the null distribution generated by random SNP sampling in relation to the corresponding value calculated with respect to the 53 psoriasis-associated SNPs (red vertical line). P-values indicate the proportion of the null distribution for which values are more extreme than those calculated based upon the 53 psoriasis-associated SNPs (one-sided hypothesis test).

## Abbreviations

cdODN: Complex decoy oligonucleotide
DEG: Differentially expressed gene
dODN: Decoy oligonucleotide
GWAS: Genome-wide association study
IHC: Immunohistochemistry
KC: Keratinocyte
NHEK: Normal human epidermal keratinocytes
PRE: Psoriasis response element
PWM: Position weight matrix
TF: Transcription factor
TSS: Transcription start site
uDBP: Unconventional DNA-binding protein
