# Supplementary material for: Psoriasis drug development and GWAS interpretation through in silico analysis of transcription factor binding sites
Source: Clin Transl Med. 2015 Mar 19;4:13. doi: 10.1186/s40169-015-0054-5 (PMC4392043; doi:10.1186/s40169-015-0054-5)
Supplement: Additional file 1: — Quality control processing of lesional (PP) and uninvolved (PN) skin microarray samples. (A) PP/PN fold-change comparison (PP/PN) between GSE51440 (HT HG-U133+ PM array plates) and datasets generated using Affymetrix Human Genome U133 Plus 2.0 arrays. Yellow ellipses outline the middle 50% of FC estimates (Mahalanobis distance). (B – I) QC metrics. We calculated (B) average background, (C) scale factor, (D) percentage of probe sets called present, (E) degradation scores, (F) NUSE median, (G) NUSE IQR, (H) RLE median and (I) RLE IQR. Yellow symbols denote excluded samples (Z scores > 3.5 in absolute value). (J) Median FC estimates among PP-increased (FC > 2; FDR < 0.05) and PP-decreased DEGs (FC < 0.50; FDR > 0.05). Two excluded outlier samples are indicated. (K) Principal component plot for GSE51440 samples (HT HG-U133+ PM array plates). (L) Principal component plot for all other samples (Affymetrix Human Genome U133 Plus 2.0 arrays). (M) Final cluster analysis of the 237 paired PP and PN samples, with distance between samples based upon PP – PN differences in RMA expression scores (i.e., Euclidean distance normalized to [0,1] interval). [file 40169_2015_54_MOESM1_ESM.pdf]

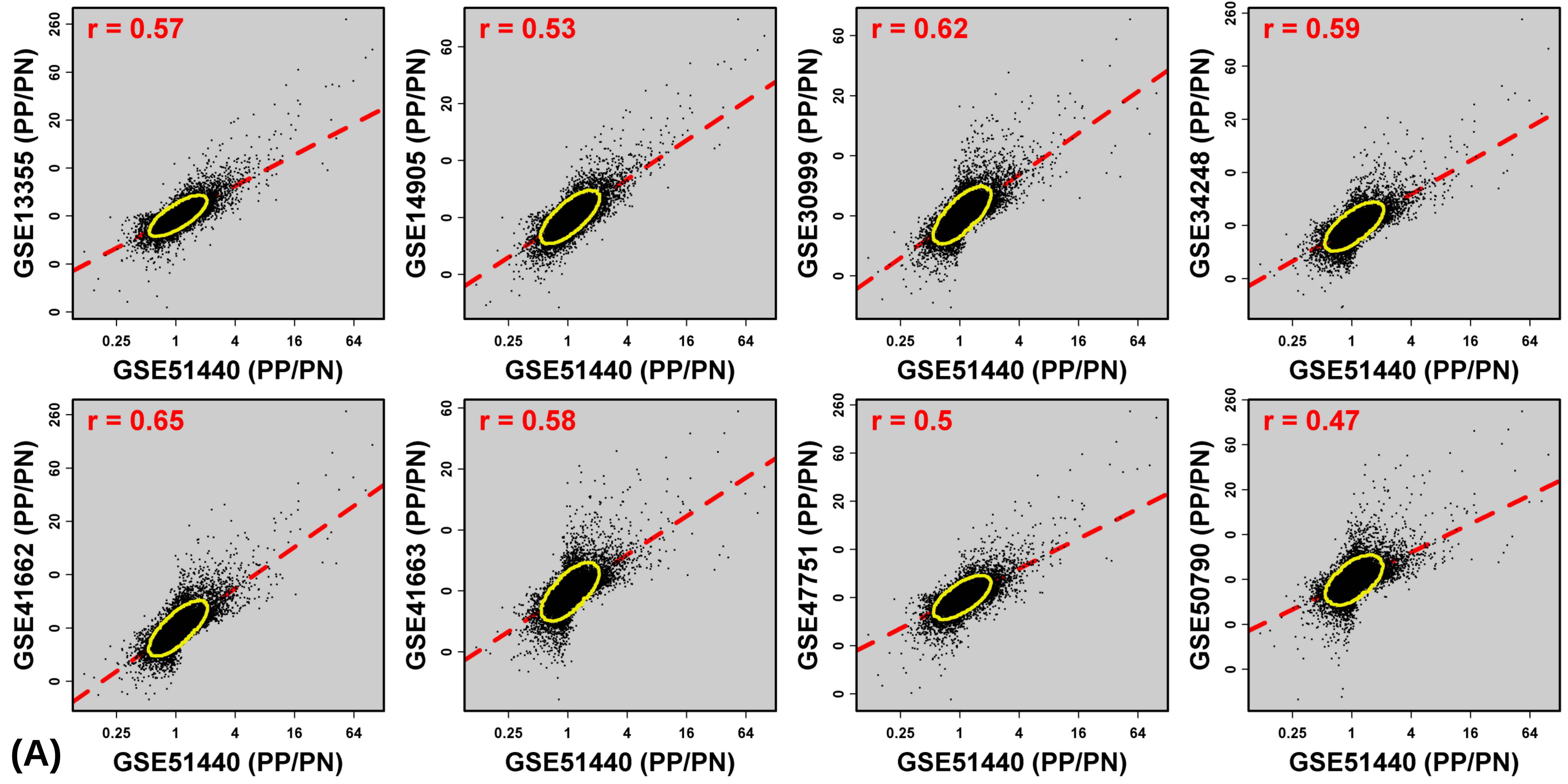
r = 0.57
r = 0.53
r = 0.62
r = 0.59
r = 0.65
r = 0.58
r = 0.5
r = 0.47
GSE13355 (PP/PN)
GSE14905 (PP/PN)
GSE30999 (PP/PN)
GSE34248 (PP/PN)
GSE41662 (PP/PN)
GSE41663 (PP/PN)
GSE47751 (PP/PN)
GSE50790 (PP/PN)
GSE51440 (PP/PN)
0.25
1
4
16
64
(A)

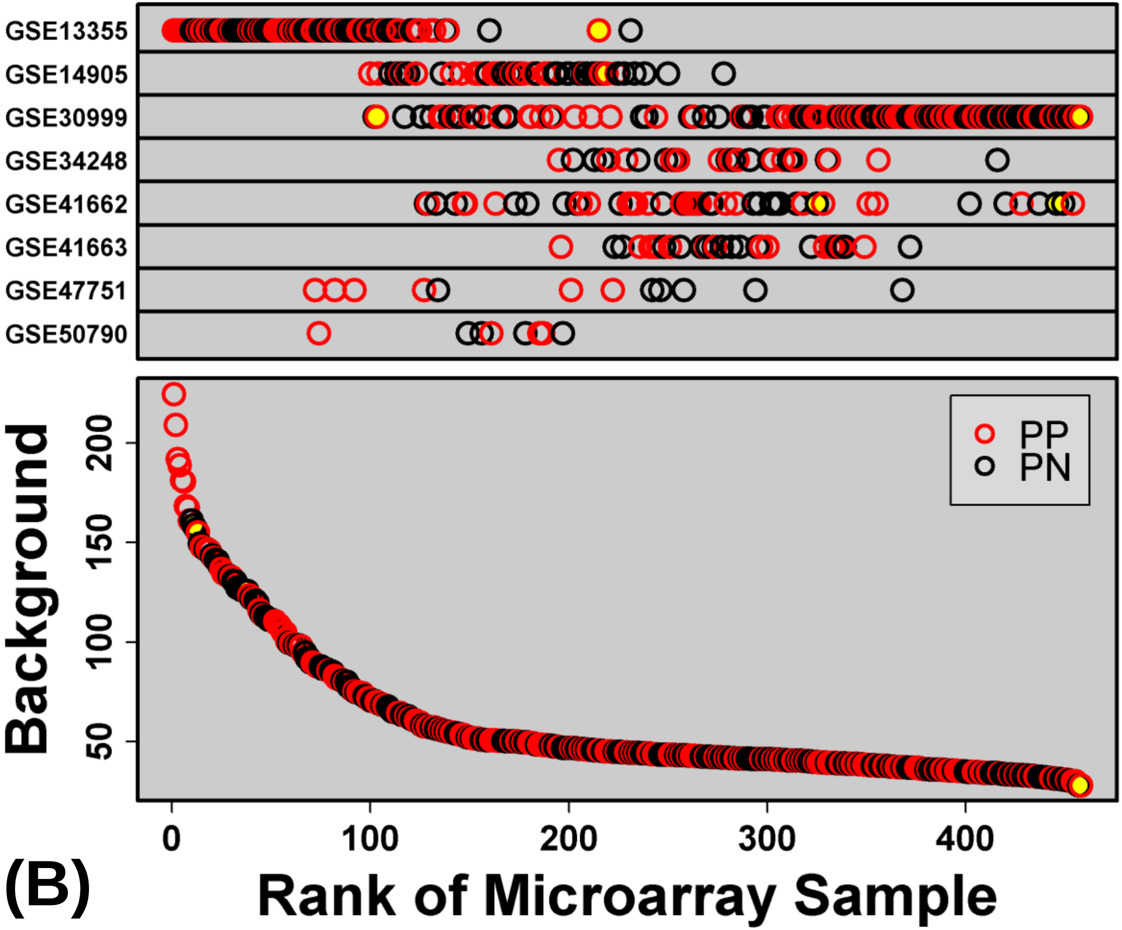

GSE13355
GSE14905
GSE30999
GSE34248
GSE41662
GSE41663
GSE47751
GSE50790
Background
50
100
150
200
PP
PN
0
100
200
300
400
(B)
Rank of Microarray Sample

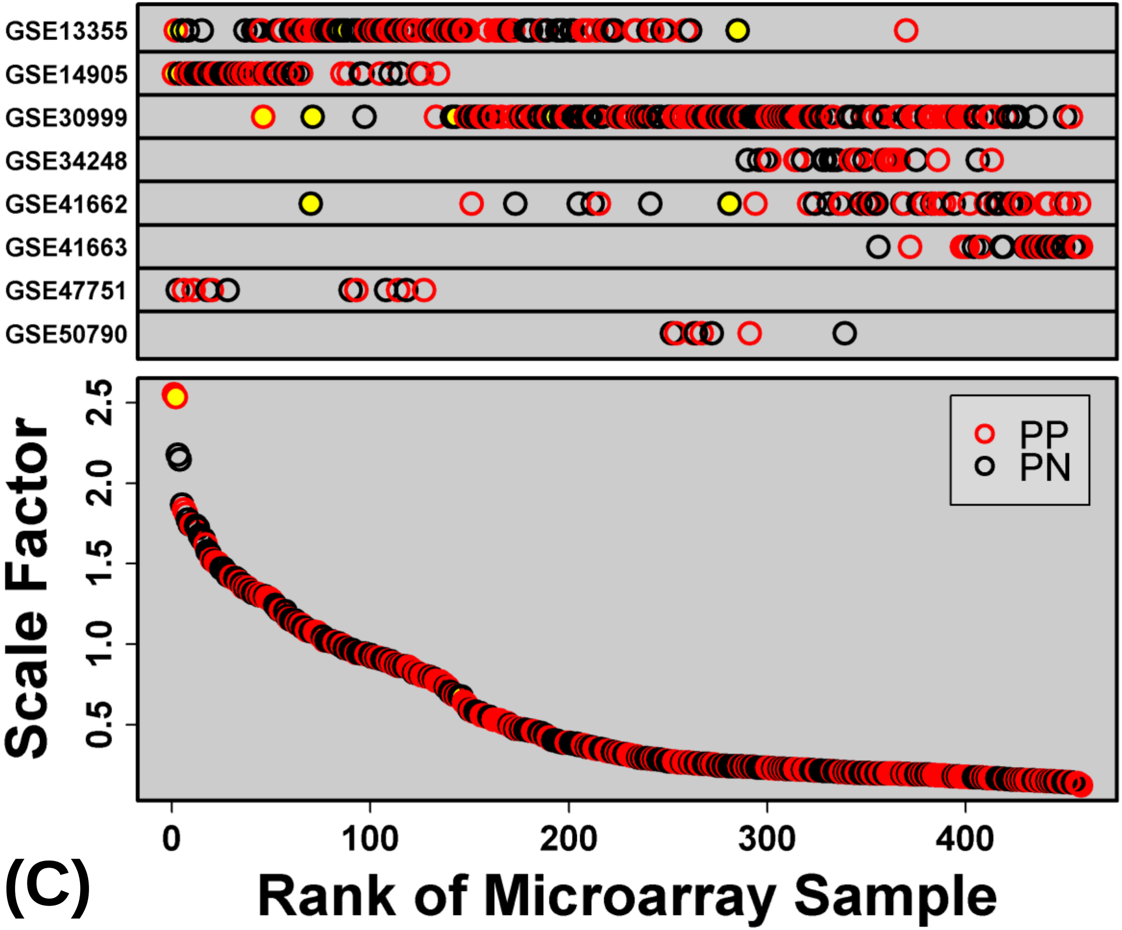
GSE13355
GSE14905
GSE30999
GSE34248
GSE41662
GSE41663
GSE47751
GSE50790
Scale Factor
0.5
1.0
1.5
2.0
2.5
PP
PN
0
100
200
300
400
Rank of Microarray Sample

(C)

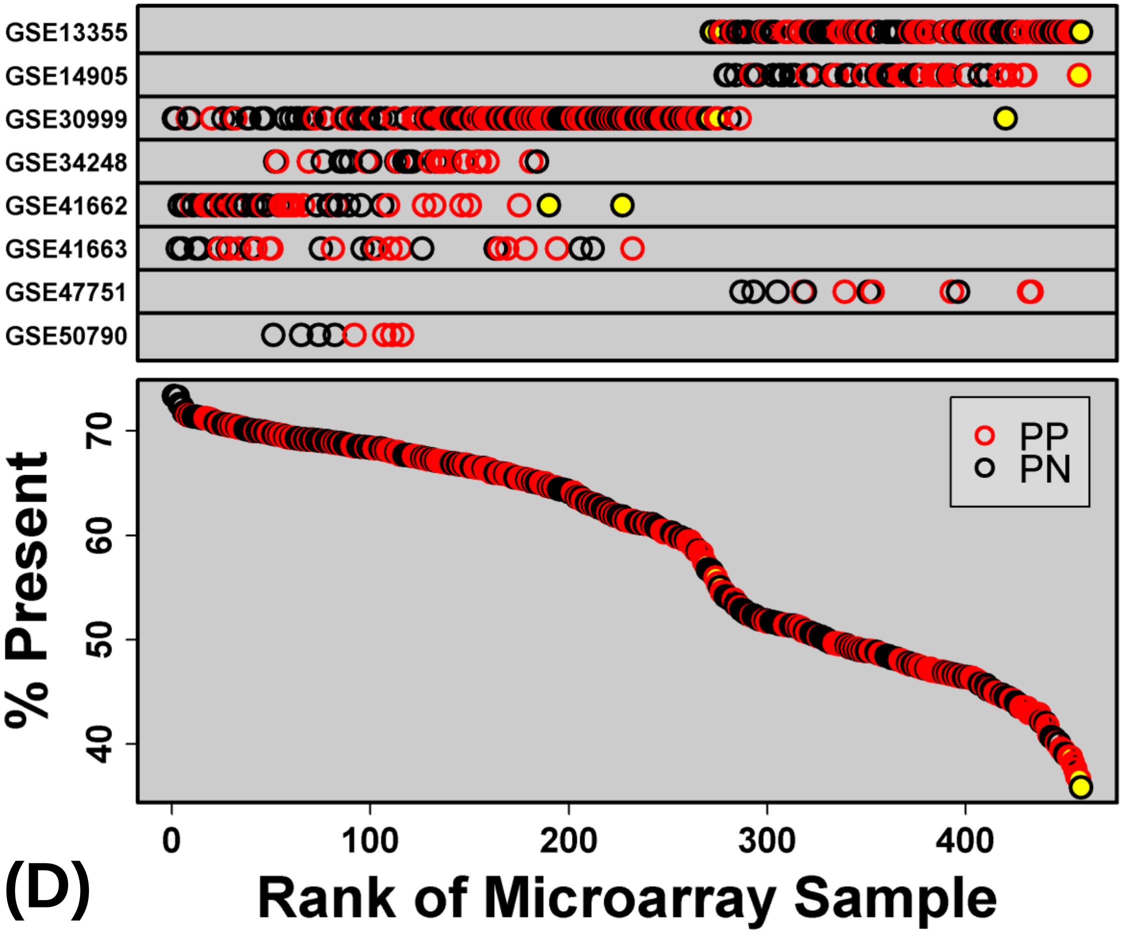

GSE13355
GSE14905
GSE30999
GSE34248
GSE41662
GSE41663
GSE47751
GSE50790
PP
PN
% Present
70
60
50
40
0
100
200
300
400
Rank of Microarray Sample
(D)

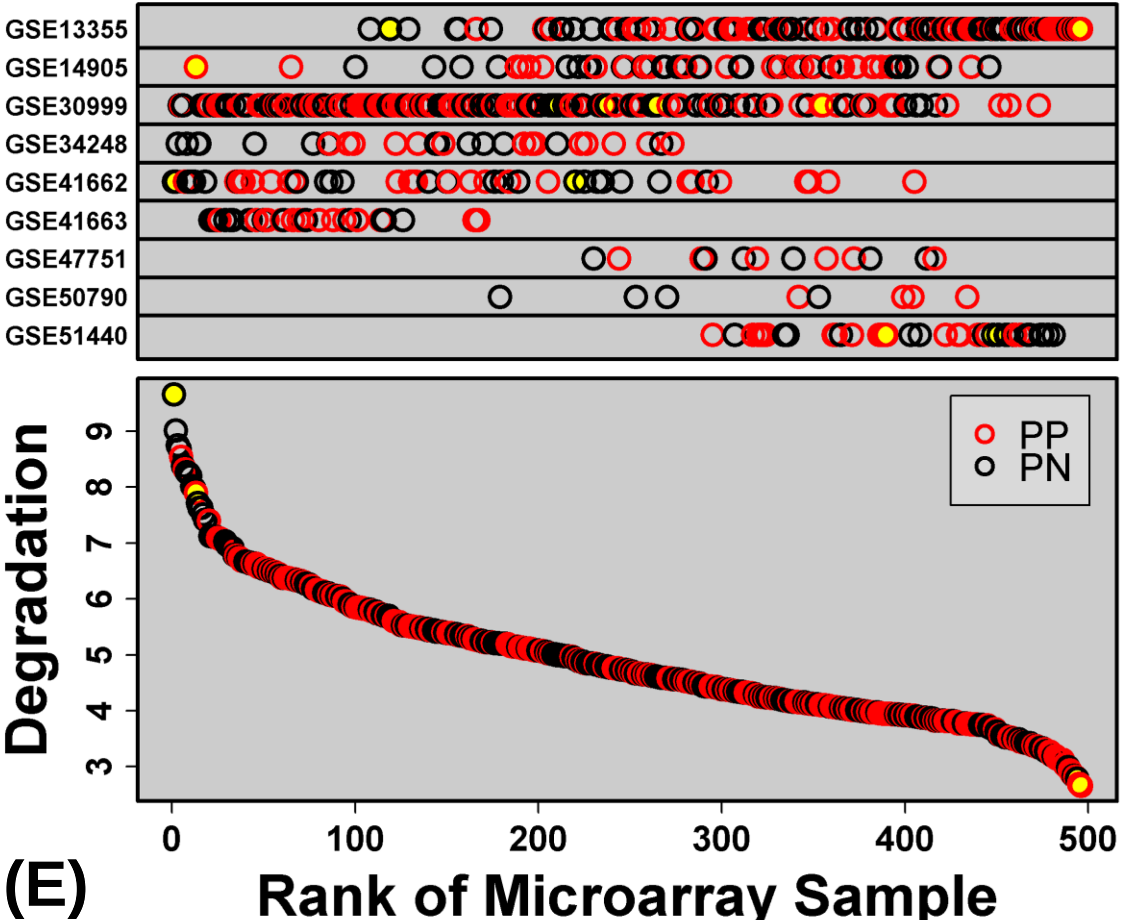
GSE13355
GSE14905
GSE30999
GSE34248
GSE41662
GSE41663
GSE47751
GSE50790
GSE51440
Degradation
3
4
5
6
7
8
9
PP
PN
0
100
200
300
400
500
Rank of Microarray Sample

(E)

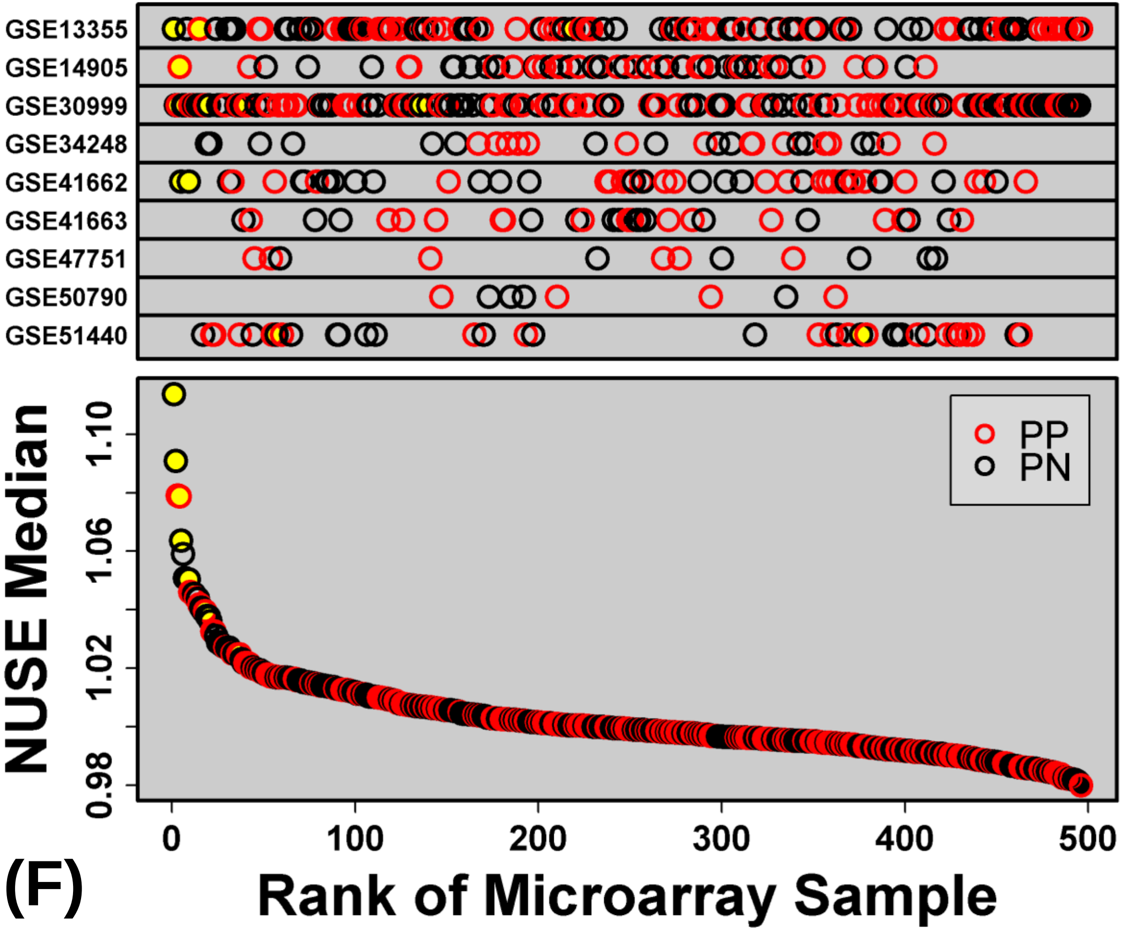
GSE13355
GSE14905
GSE30999
GSE34248
GSE41662
GSE41663
GSE47751
GSE50790
GSE51440
NUSE Median
0.98
1.02
1.06
1.10
PP
PN
0
100
200
300
400
500
Rank of Microarray Sample

(F)

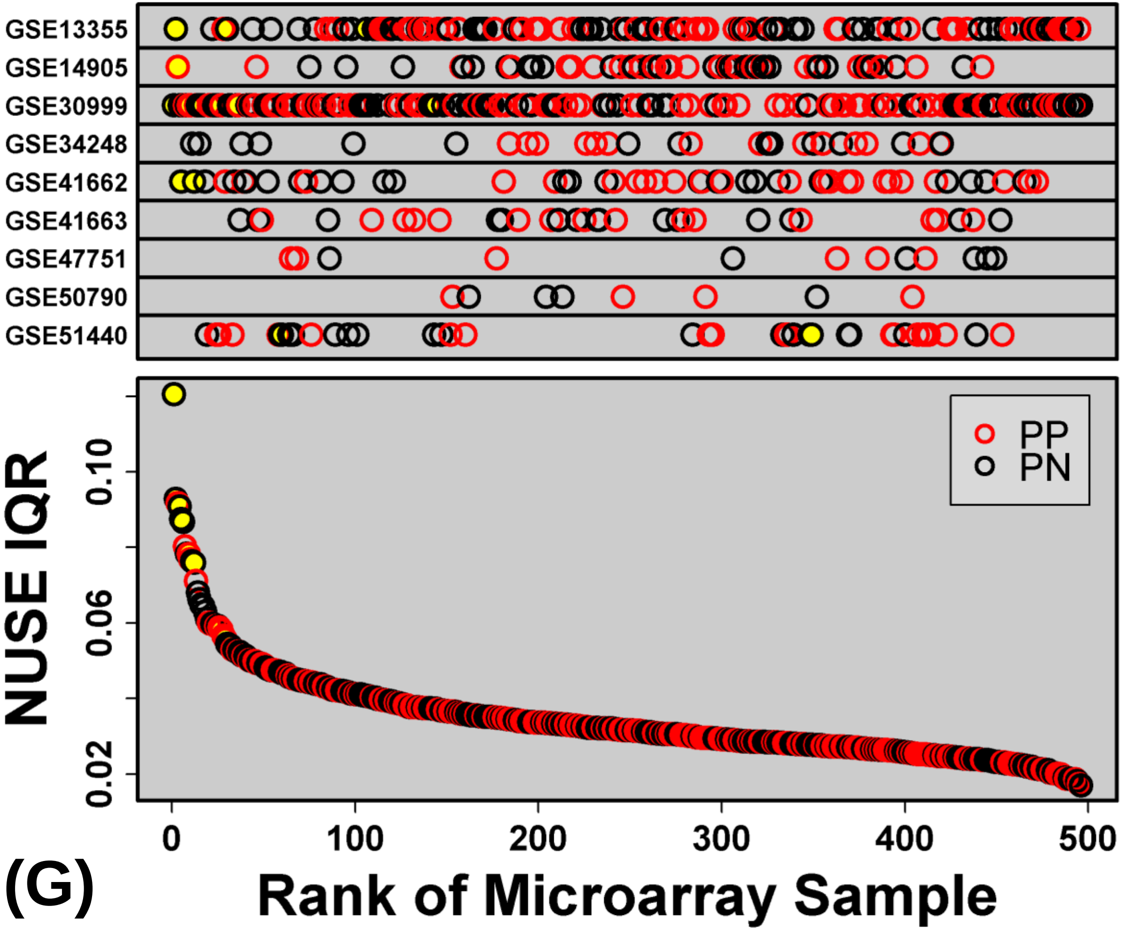

GSE13355
GSE14905
GSE30999
GSE34248
GSE41662
GSE41663
GSE47751
GSE50790
GSE51440
NUSE IQR
0.02
0.06
0.10
0
100
200
300
400
500
PP
PN
Rank of Microarray Sample

(G)

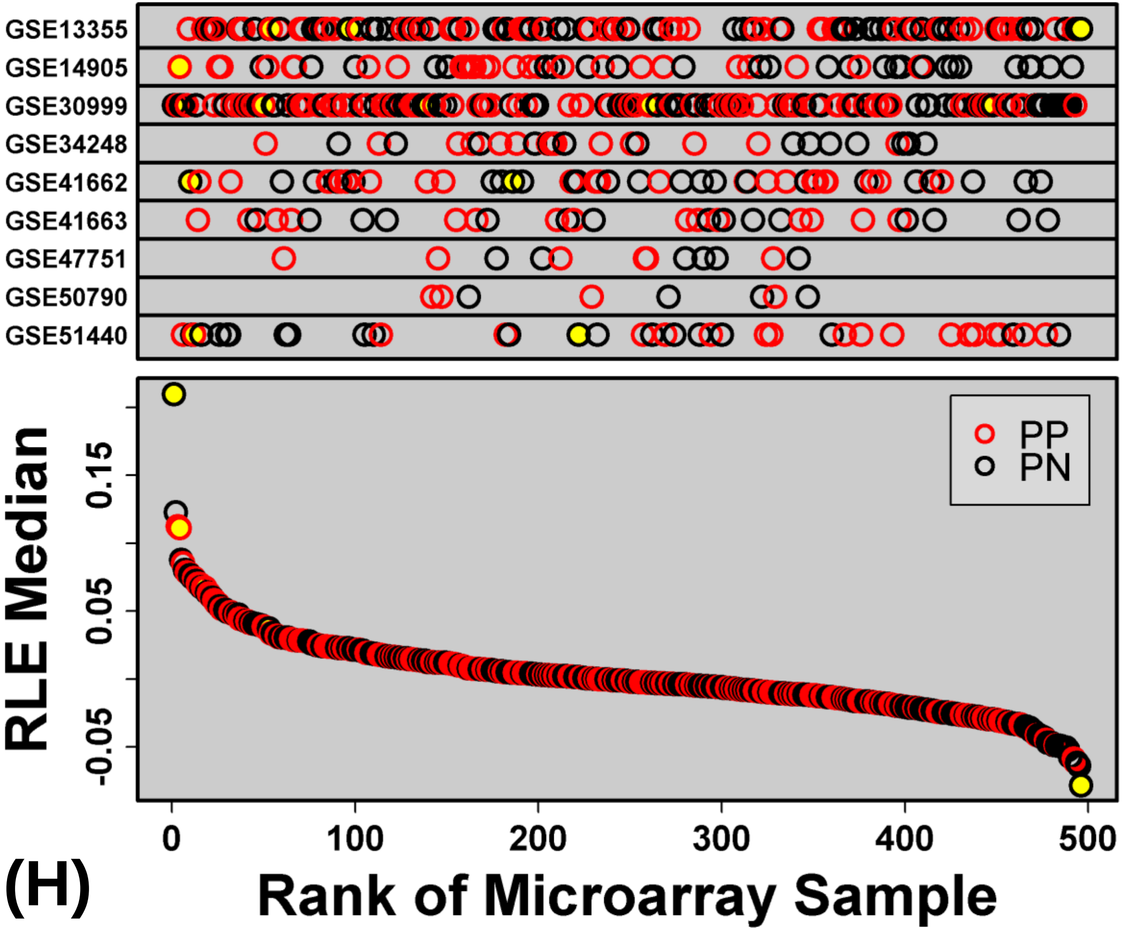
GSE13355
GSE14905
GSE30999
GSE34248
GSE41662
GSE41663
GSE47751
GSE50790
GSE51440
RLE Median
0.15
0.05
-0.05
PP
PN
0
100
200
300
400
500
(H)
Rank of Microarray Sample

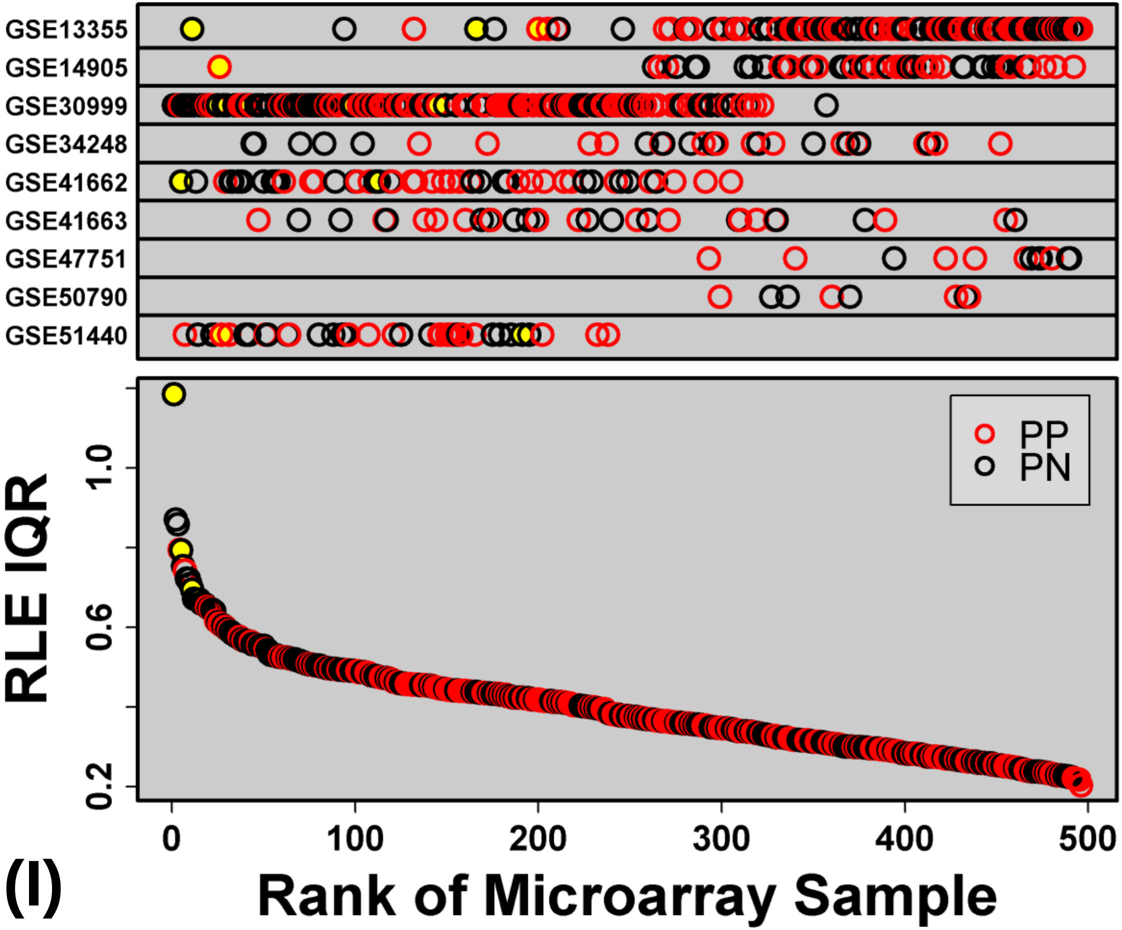
GSE13355
GSE14905
GSE30999
GSE34248
GSE41662
GSE41663
GSE47751
GSE50790
GSE51440
PP
PN
RLE IQR
1.0
0.6
0.2
0
100
200
300
400
500
Rank of Microarray Sample
(I)

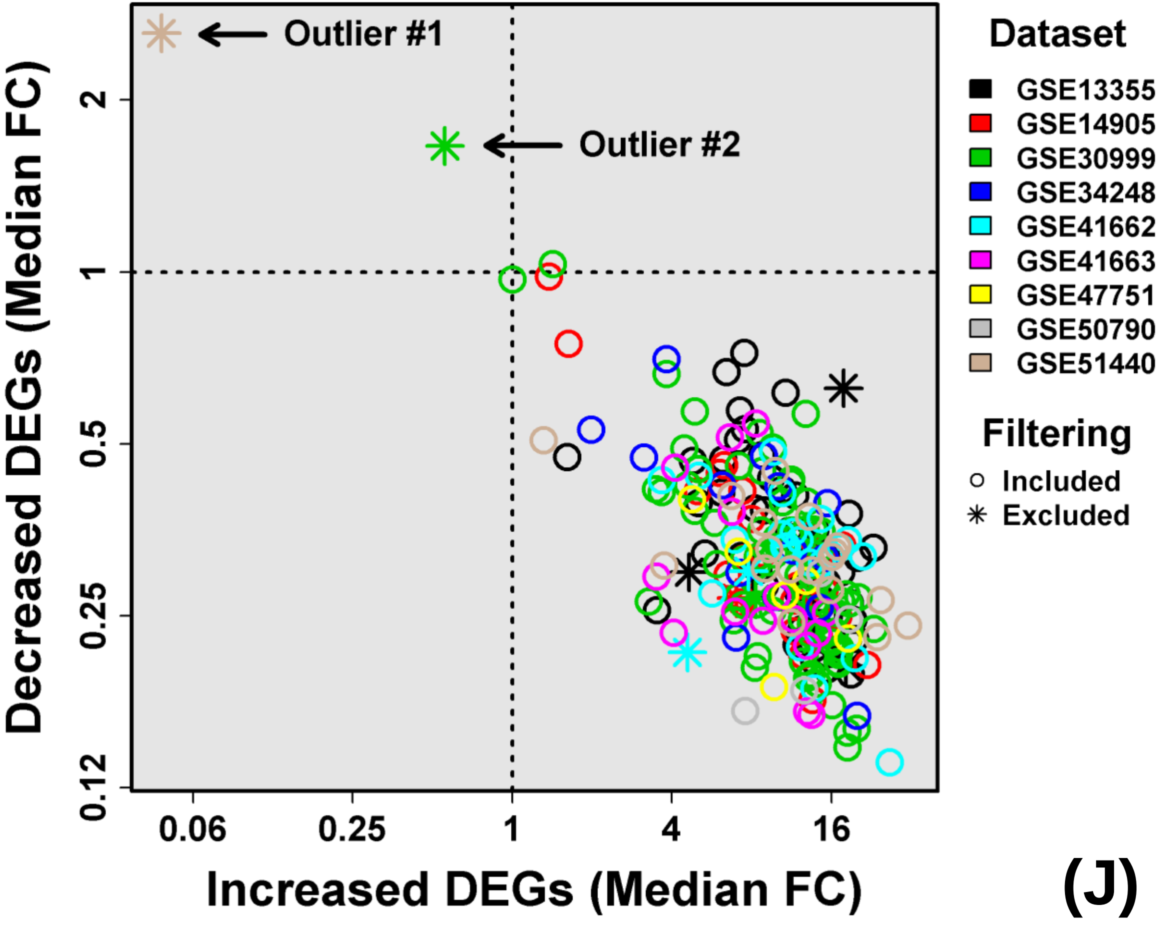
Outlier #1
Outlier #2
Decreased DEGs (Median FC)
Increased DEGs (Median FC)
0.12
0.25
0.5
1
2
0.06
0.25
1
4
16
Dataset
GSE13355
GSE14905
GSE30999
GSE34248
GSE41662
GSE41663
GSE47751
GSE50790
GSE51440
Filtering
Included
Excluded
(J)

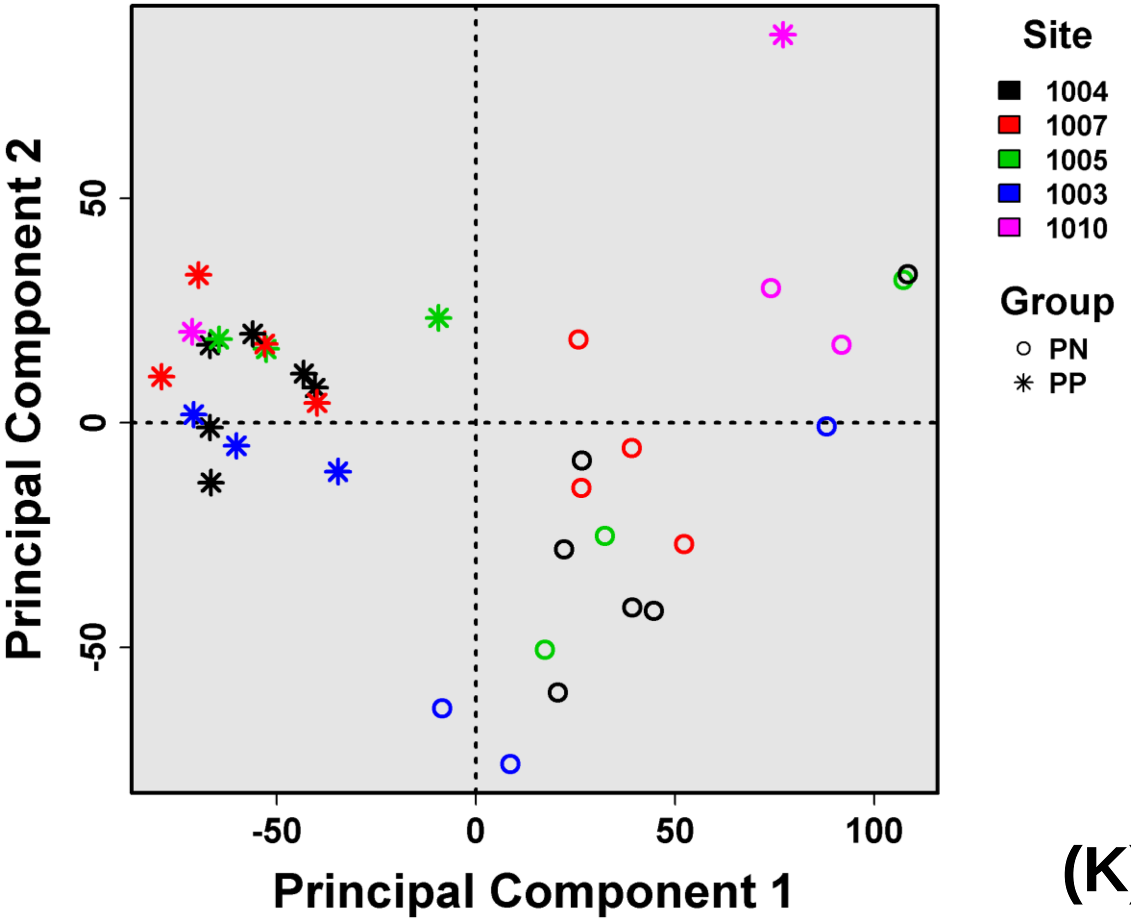

Principal Component 2
Principal Component 1
50
0
-50
-50
0
50
100
Site
1004
1007
1005
1003
1010
Group
PN
PP

(K)

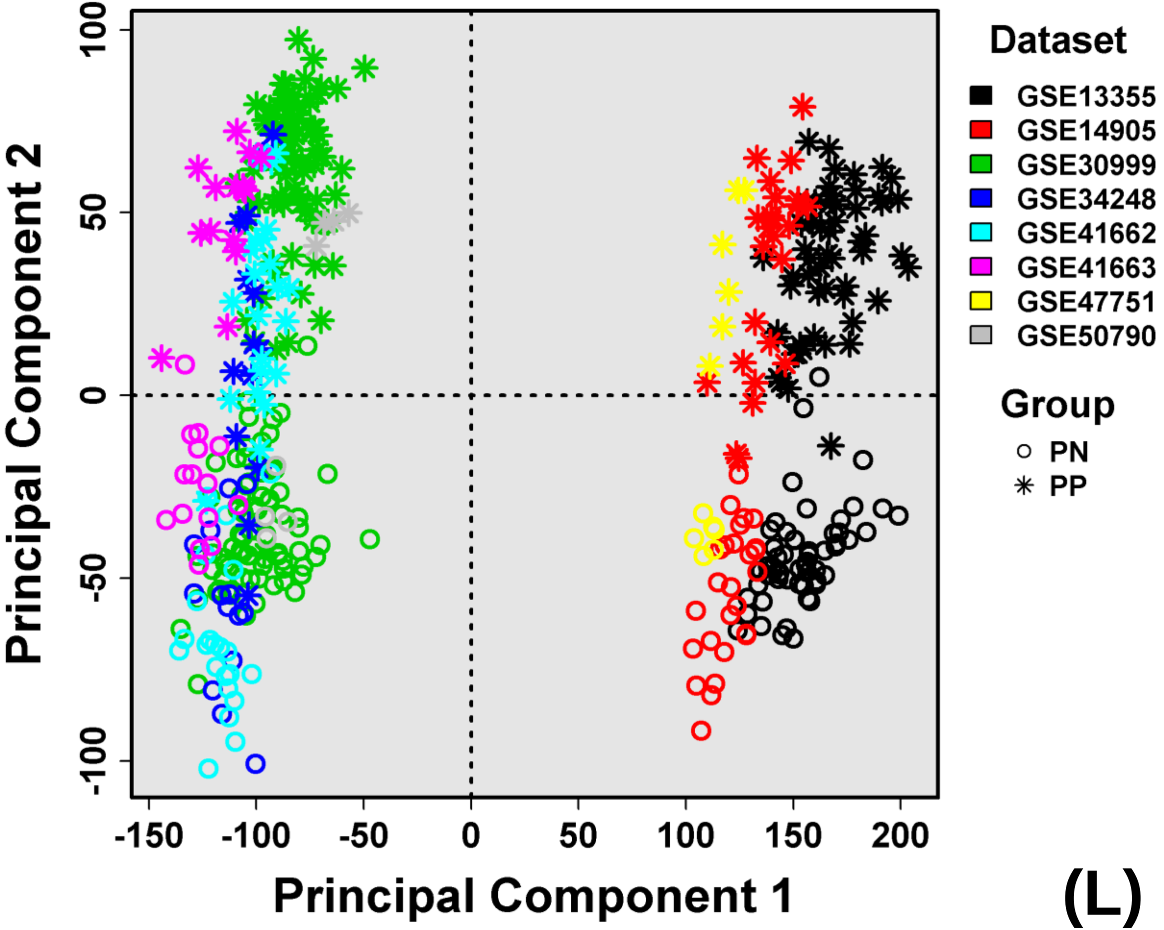

Dataset
GSE13355
GSE14905
GSE30999
GSE34248
GSE41662
GSE41663
GSE47751
GSE50790
Group
PN
PP
Principal Component 1
Principal Component 2
-150
-100
-50
0
50
100
150
200
100
50
0
-50
-100

(L)

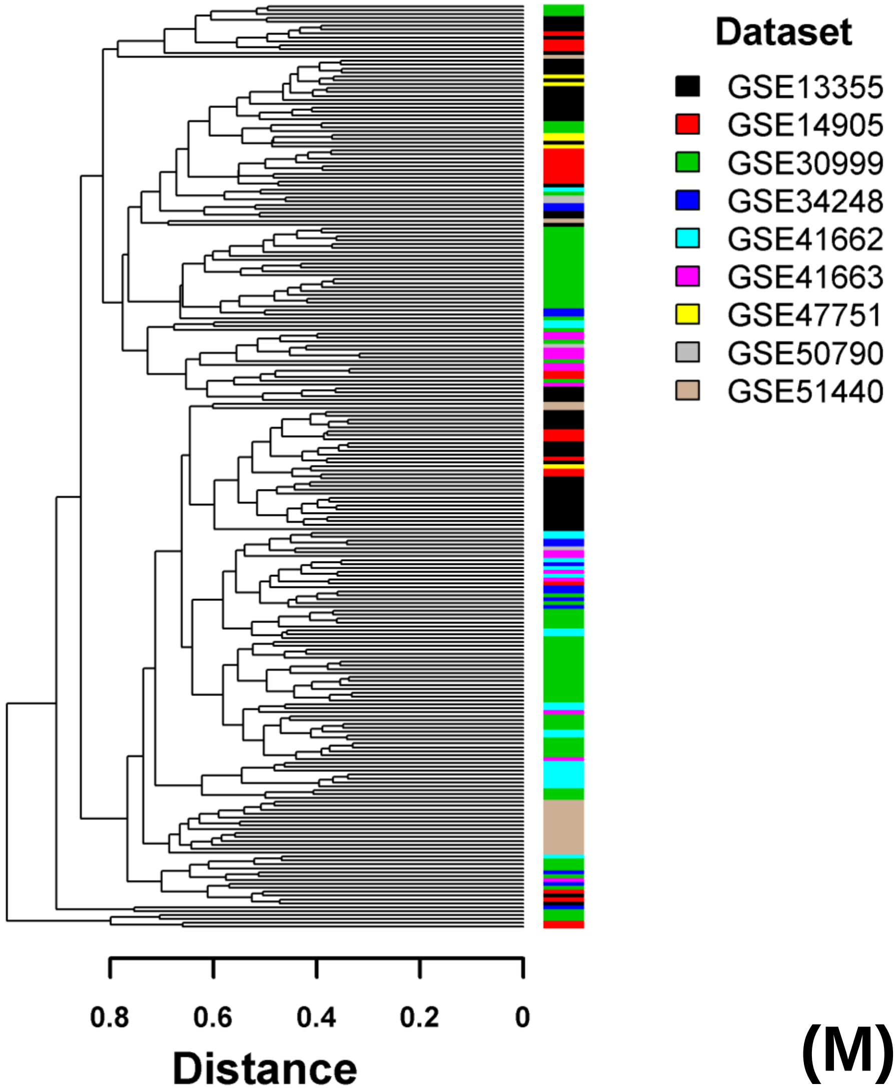

Dataset
GSE13355
GSE14905
GSE30999
GSE34248
GSE41662
GSE41663
GSE47751
GSE50790
GSE51440
0.8
0.6
0.4
0.2
0
Distance

(M)
