# Supplementary material for: Psoriasis drug development and GWAS interpretation through in silico analysis of transcription factor binding sites
Source: Clin Transl Med. 2015 Mar 19;4:13. doi: 10.1186/s40169-015-0054-5 (PMC4392043; doi:10.1186/s40169-015-0054-5)
Supplement: Additional file 2: — Construction of motif dictionary by integration across seven sources. The initial set of 4378 motifs was filtered to remove redundant motifs and motifs with low information content, yielding the final set of 2935 motifs used in our analyses (see Methods ). The table lists the number of motifs obtained from each source before and after filtering. The number of unique human genes associated with motifs is listed in parentheses. [file 40169_2015_54_MOESM2_ESM.pdf]

Additional File 2. Construction of motif dictionary by integration across seven sources. The table lists the number of motifs obtained from each source before and after filtering. The number of unique human genes associated with motifs is listed in parentheses. The initial set of 4378 motifs was filtered to remove redundant motifs and motifs with low information content, yielding the final set of 2935 motifs used in our analyses.

Source	No. Motifs (pre-filter)	No. Motifs (post-filter)
hPDI[1]	436 (436)	420 (420)
Jaspar[2]	633 (412)	285 (228)
Jolma Taipale 2013[3]	843 (404)	333 (245)
Kheradpour Kellis 2013[4]	292 (94)	220 (84)
TRANSFAC[5]	1799 (801)	1449 (720)
UniPROBE[6]	296 (277)	192 (185)
Wang Weng 2013[7]	79 (110)	36 (56)
Total	4378 (1559)	2935 (1422)

[1]Xie et al. 2010, Bioinformatics 26:287-289
[2]Mathelier 2014, Nucleic Acids Res 42:D142-147
[3]Jolma et al. 2013, Cell 152:327-339
[4]Kheradpour and Kellis 2014, Nucleic Acids Res 42:2976-2987
[5]Matys et al. 2006, Nucleic Acids Res 34:D108-110
[6]Robasky and Bulyk 2011, Nucleic Acids Res 39:D124-D128
[7]Wang et al. 2012, Genome Res 22:1798-1812
