# Supplementary figures and images for: Psoriasis drug development and GWAS interpretation through in silico analysis of transcription factor binding sites
Source: Clin Transl Med. 2015 Mar 19;4:13. doi: 10.1186/s40169-015-0054-5 (PMC4392043; doi:10.1186/s40169-015-0054-5)

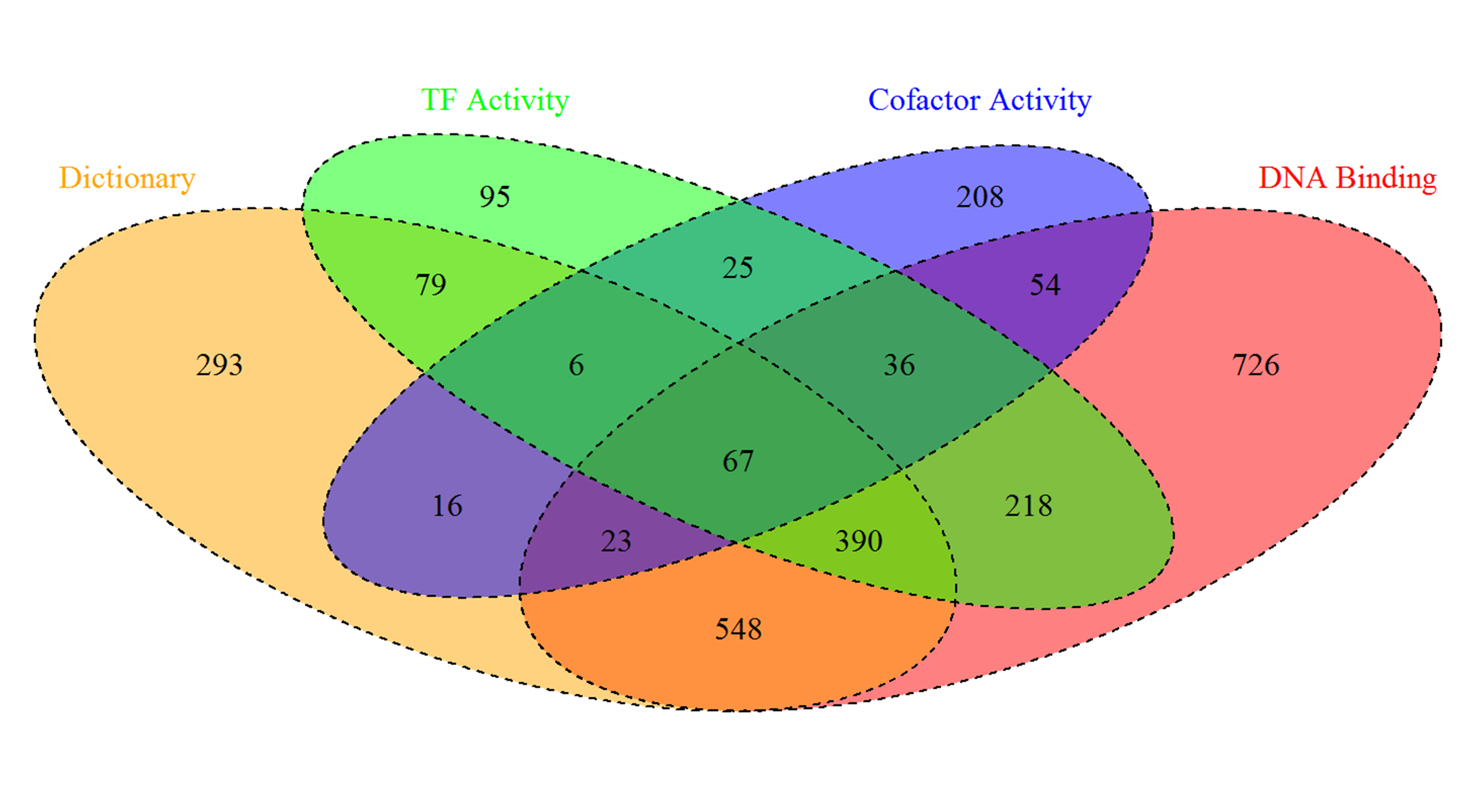

Supplement: Additional file 3: — Gene ontology (GO) biological process (BP) terms and genes associated with DNA motifs within our dictionary. The 2935 PWM motifs were associated with 1422 unique human genes. The Venn diagram shows the number of these genes associated with GO biological process terms “transcription factor activity” (GO:0003700), “transcription cofactor activity” (GO:0003712) and “DNA binding” (GO:0003677). [file 40169_2015_54_MOESM3_ESM.tiff]

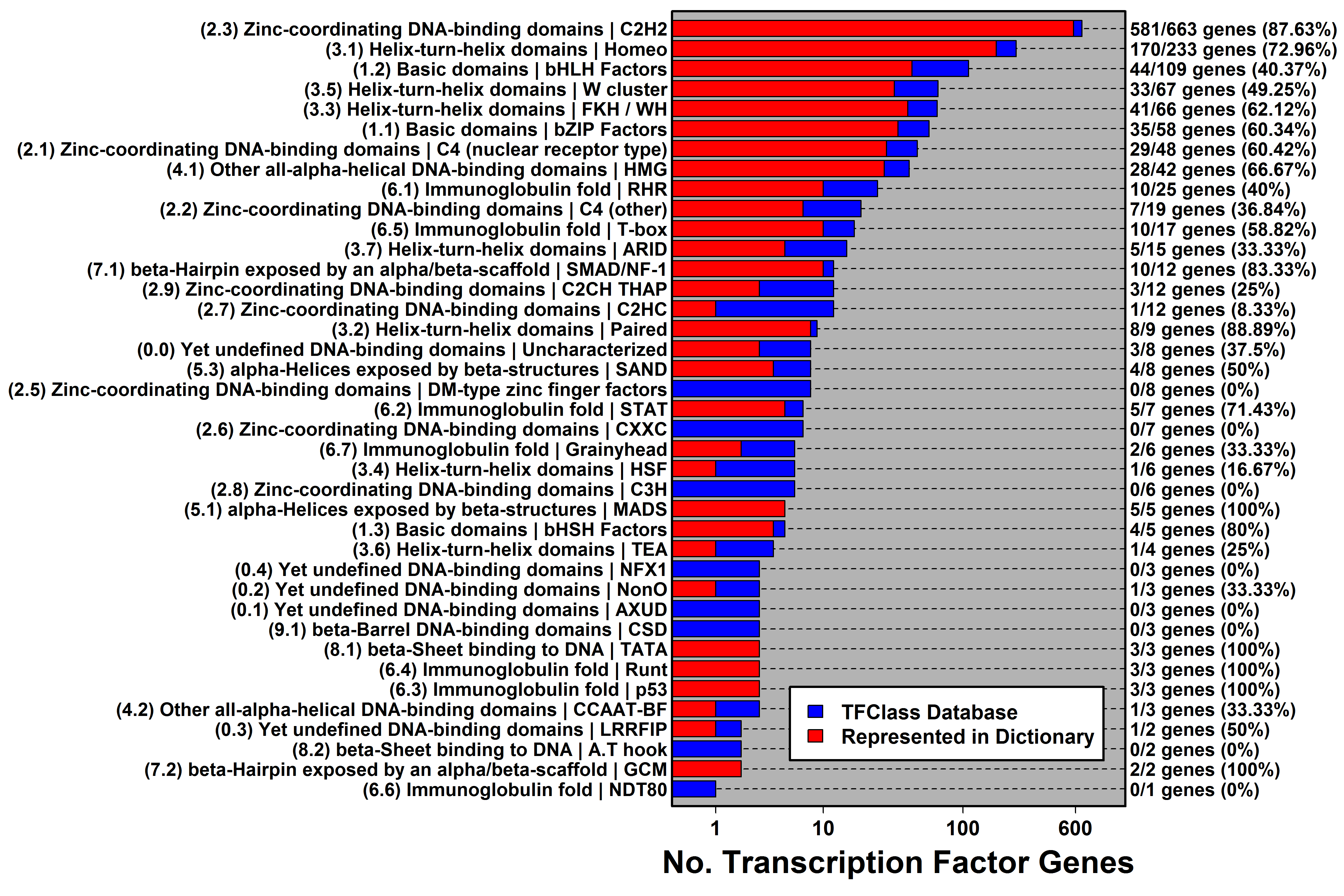

Supplement: Additional file 4: — Transcription factor DNA-binding domain superfamily and class groups. 1509 human TF-encoding genes from the TFclass database were assigned to superfamily and class groups based upon their DNA-binding domain. We identified the largest superfamily and class groups and determined the number of genes in each group associated with at least one PWM model from our dictionary of 2935 motifs (red). [file 40169_2015_54_MOESM4_ESM.tiff]

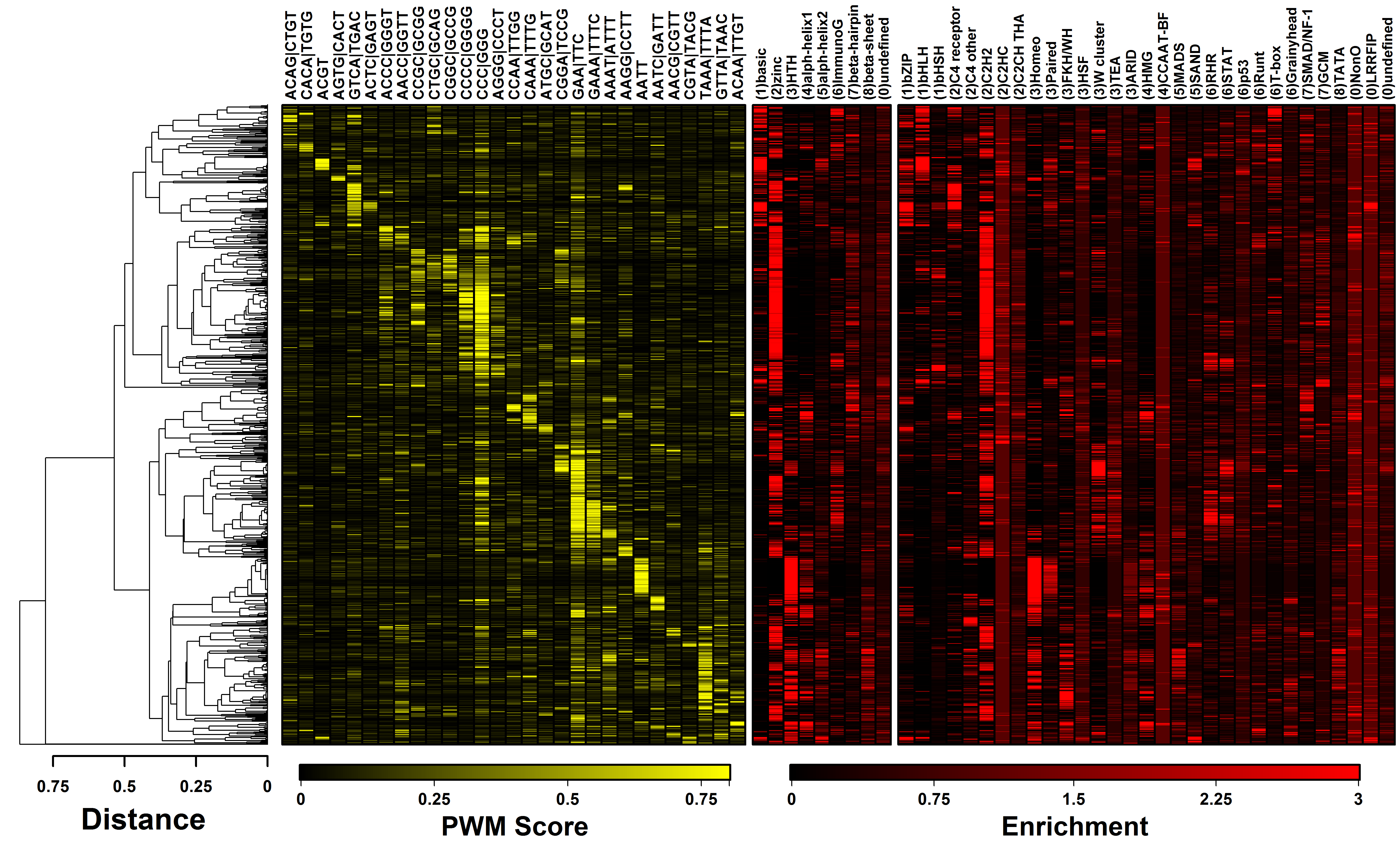

Supplement: Additional file 5: — Cluster analysis of the 2935 PWM models included within our motif dictionary. Motifs were clustered as described in Figures 2A and 3A. The yellow-black heatmap shows motif k-mer scores (top margin). Red-black heatmaps show enrichment scores indicating how well a given PWM matches other PWMs associated with different DNA-binding domain superfamily and class groups (TFclass database). [file 40169_2015_54_MOESM5_ESM.tiff]

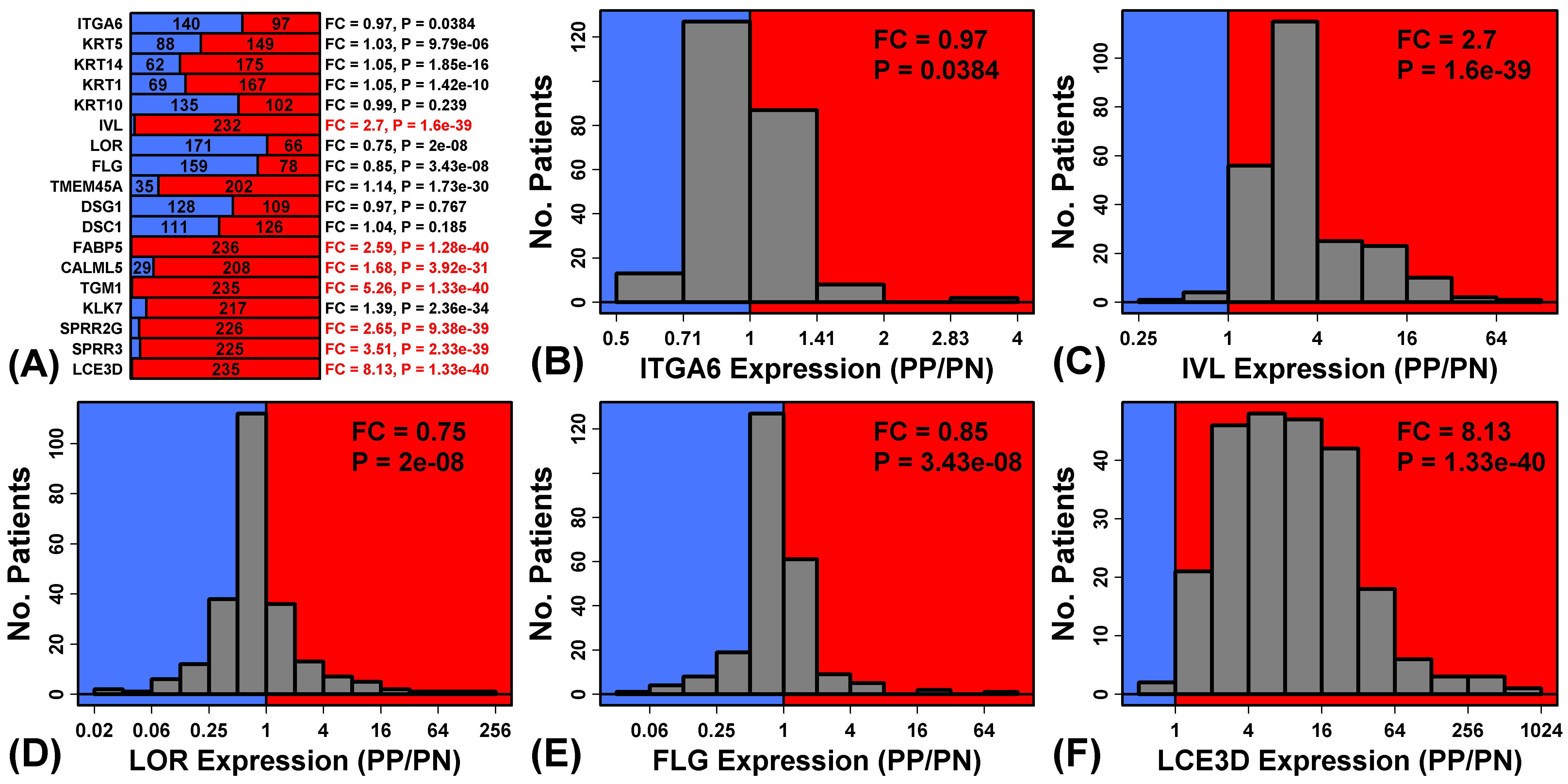

Supplement: Additional file 7: — KC proliferation and differentiation markers in psoriasis lesions and uninvolved skin ( n = 237 patients). (A) KC proliferation and differentiation markers (left margin). The number of patients showing increased (red) or decreased (blue) expression is indicated for each gene, along with the median PP/PN fold-change and p-value (right margin; Wilcoxon rank sum test). (B – F) Distribution of FC estimates across all patients for selected genes. [file 40169_2015_54_MOESM7_ESM.tiff]

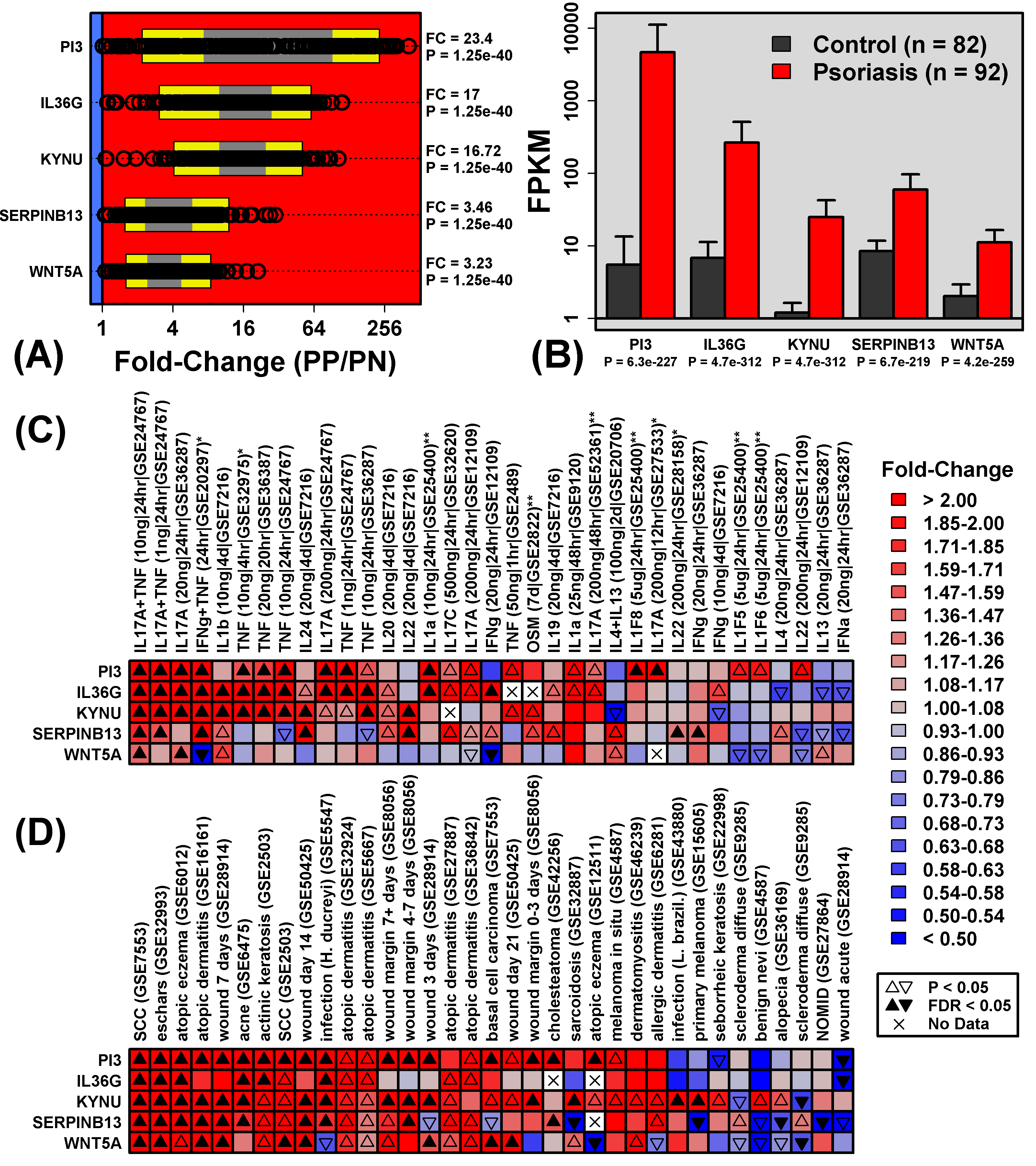

Supplement: Additional file 8: — Hallmark psoriasis genes with near-universally increased expression in lesional skin ( PI3, IL36G, KYNU, SERPINB13 and WNT5A ). We identified five genes for which expression was higher in lesional (PP) as compared to uninvolved skin (PN) for all patients ( n = 237). (A) Distribution of PP/PN fold-change (FC) estimates among patients (grey boxes: middle 50%; yellow boxes: middle 80%). Median FC estimates and p-values are listed (right margin). (B) Mean expression in lesional (PP) and normal skin (NN) from control subjects (RNA-seq, GSE54456). Expression is measured using fragments per kilobase of transcript per million mapped reads (FPKM). (C) Cytokine responses in cultured KCs (*HaCAT KCs; **reconstituted epidermis). The cytokine, concentration (per μL), duration of cytokine treatment, and Gene Expression Omnibus series identifier is listed for each experiment (top margin). (D) Skin disease panel. The expression of each gene was evaluated in other skin diseases and compared to its expression in normal skin. [file 40169_2015_54_MOESM8_ESM.tiff]

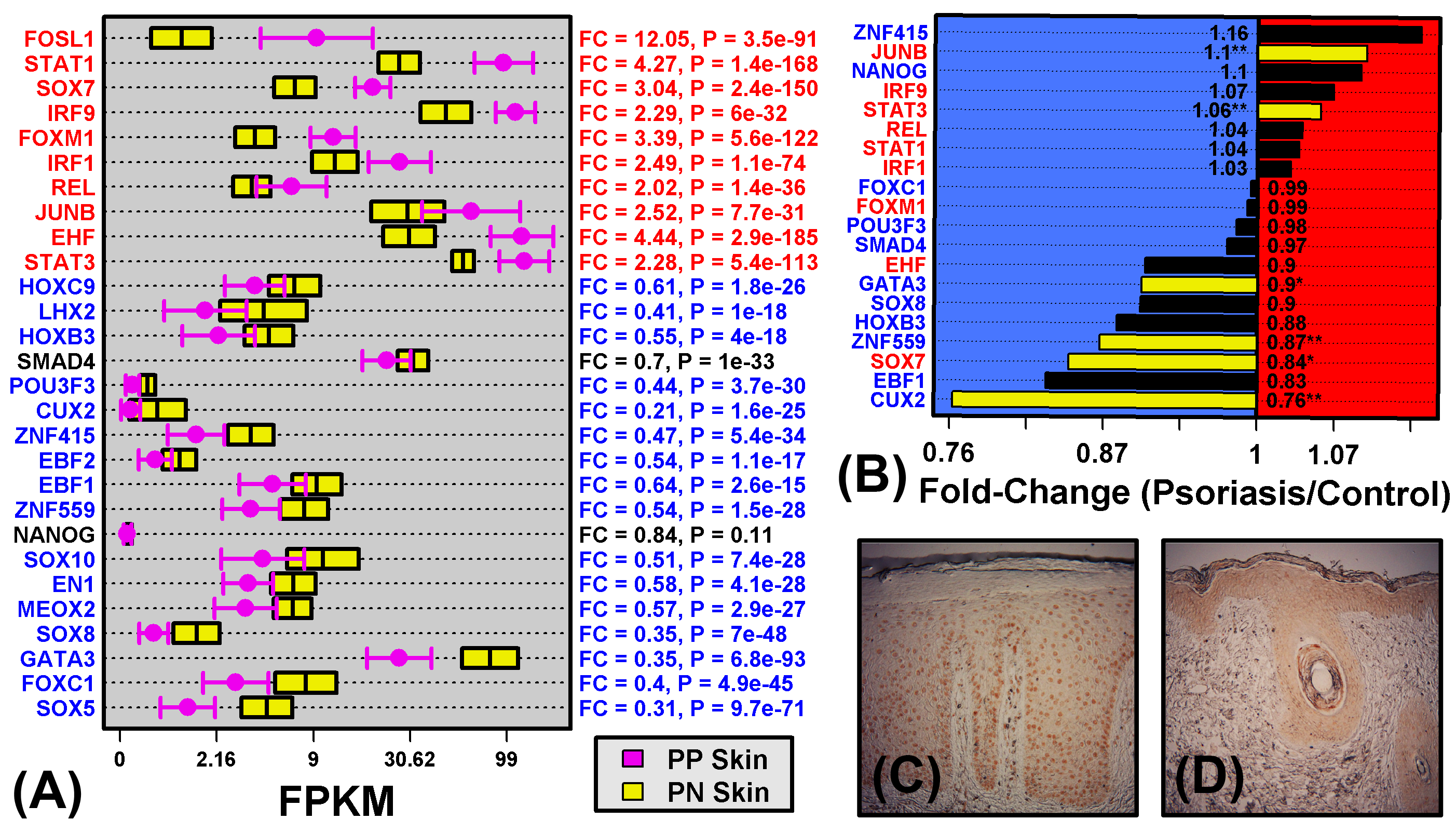

Supplement: Additional file 10: — TFs encoded by psoriasis DEGs that interact with PREs. (A) Expression in psoriasis lesions and normal skin from control subjects (RNA-seq; GSE54456). Symbols denote average expression (±1 standard deviation). Expression is measured using fragments per kilobase of transcript per million mapped reads (FPKM). (B) Expression in blood from psoriasis patients and control subjects (GSE55201). In (A) and (B), genes in red and blue font have increased and decreased expression in PP vs. PN skin, respectively (n = 237 patients, microarray). (C) IHC stain for ETS homologous Factor (EHF) in PP skin (10X magnification). (D) IHC stain for EHF in PN skin (10X magnification). [file 40169_2015_54_MOESM10_ESM.tiff]

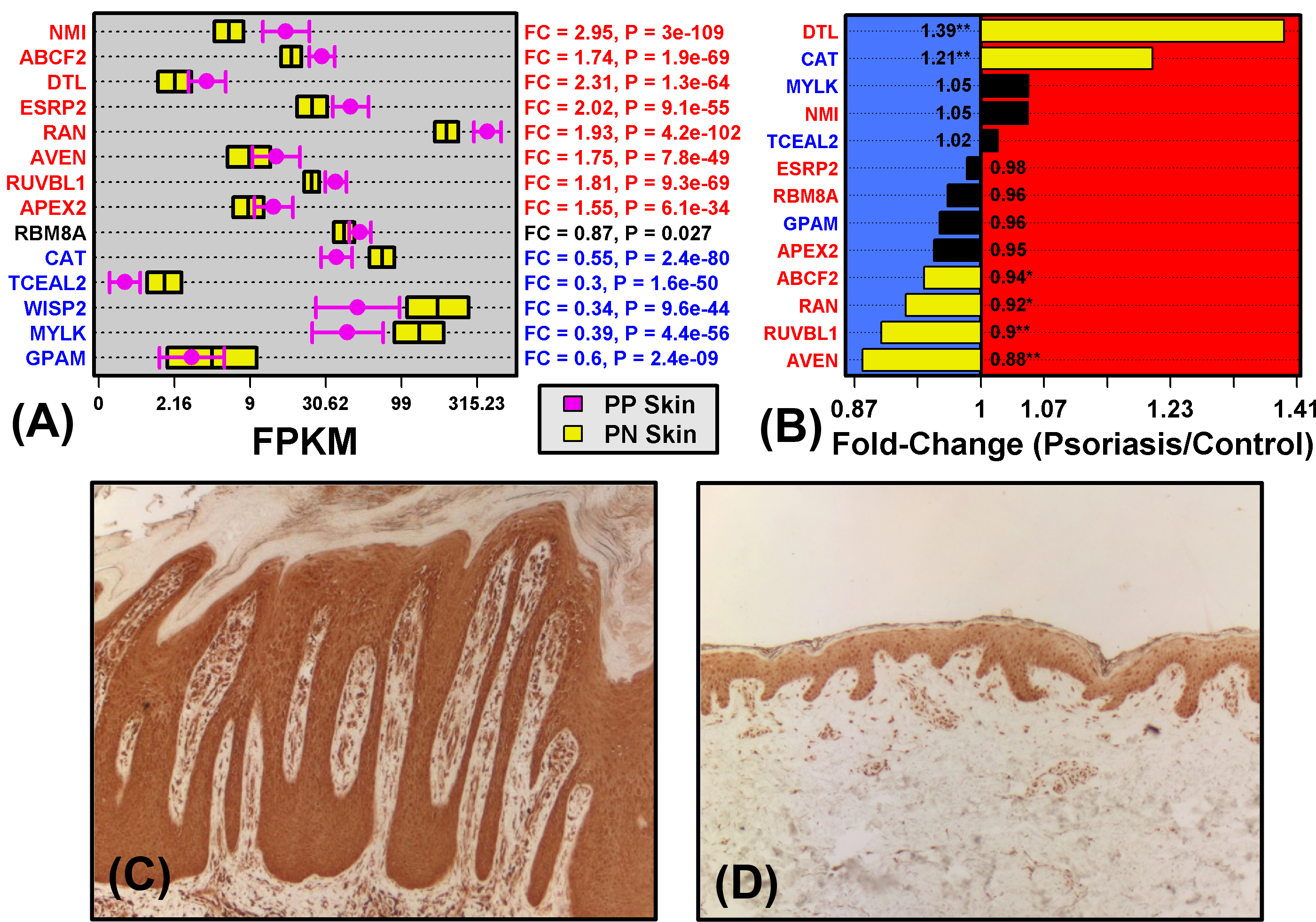

Supplement: Additional file 11: — uDBPs encoded by psoriasis DEGs that interact with PREs. (A) Expression in psoriasis lesions and normal skin from control subjects (RNA-seq; GSE54456). Symbols denote average expression (±1 standard deviation). Expression is measured using fragments per kilobase of transcript per million mapped reads (FPKM). (B) Expression in blood from psoriasis patients and control subjects (GSE55201). In (A) and (B), genes in red and blue font have increased and decreased expression in PP vs. PN skin, respectively (n = 237 patients, microarray). (C) IHC stain for apoptosis caspase activation inhibitor (AVEN) in PP skin (10X magnification). (D) IHC stain for AVEN in PN skin (10X magnification). [file 40169_2015_54_MOESM11_ESM.tiff]

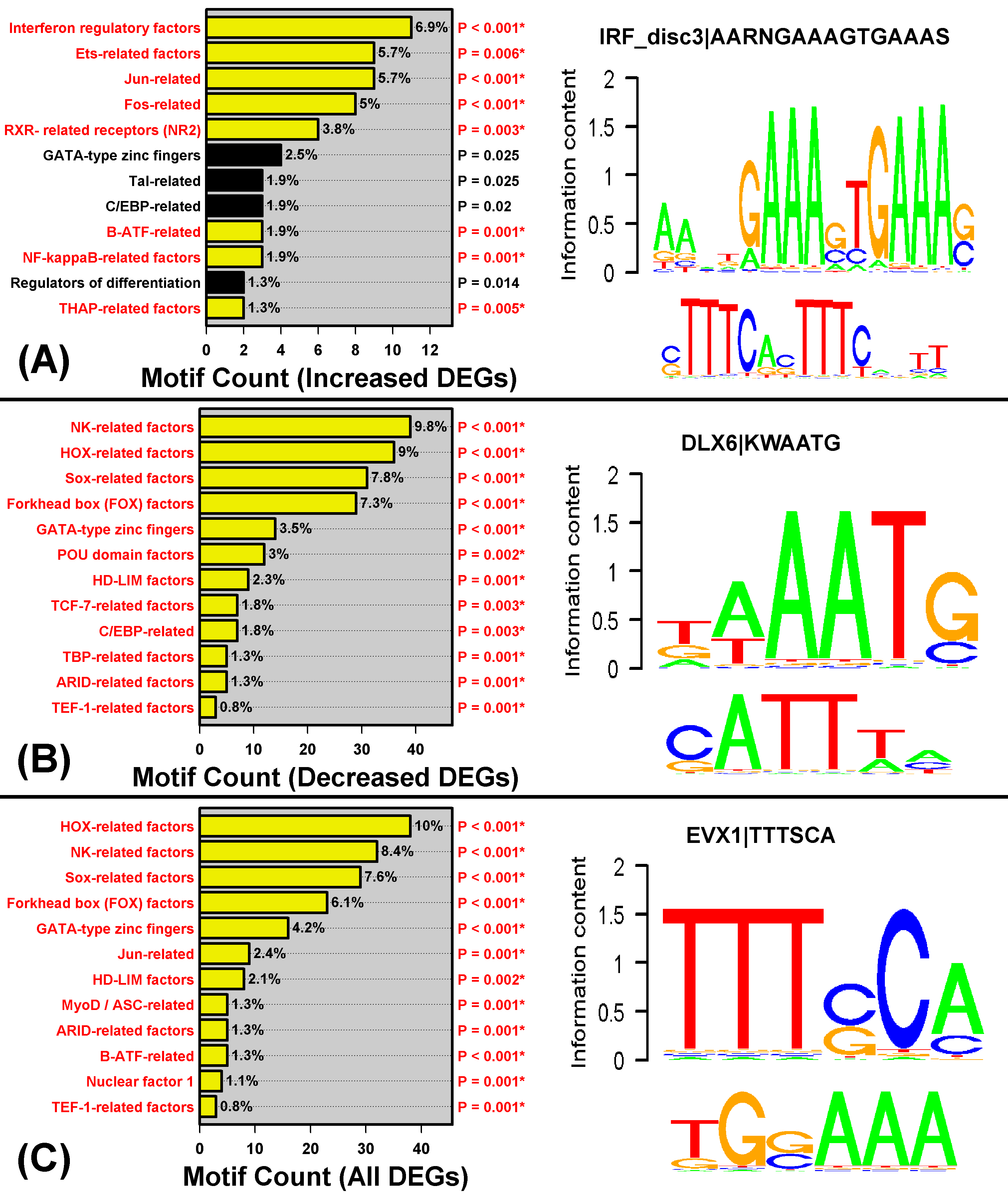

Supplement: Additional file 12: — DNA-binding domain families associated with psoriasis DEGs. DNA-binding domain families most strongly overrepresented among PRE motifs enriched in sequences upstream of (A) PP-increased DEGs, (B) PP-decreased DEGs, (C) PP-increased and PP–decreased DEGs. P-values assess whether motifs belonging to a family are significantly overrepresented among the set of PRE motifs associated with (A) – (C), respectively (right margin; Fisher’s Exact Test). Example sequence logos are shown for the most strongly overrepresented TF families (i.e., interferon regulatory factors, part A; NK-related factors, part B; HOX-related factors, part C). [file 40169_2015_54_MOESM12_ESM.tiff]

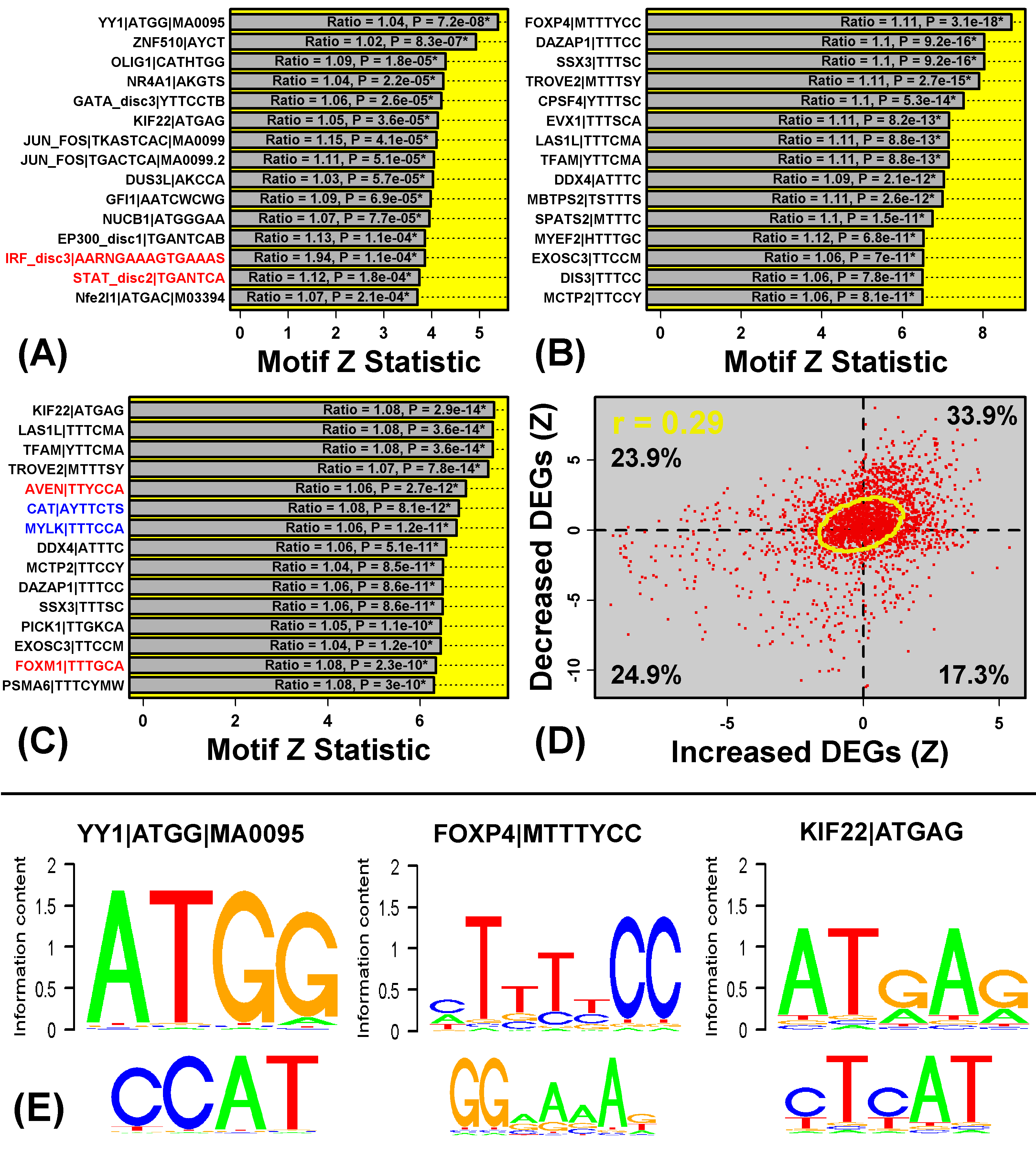

Supplement: Additional file 13: — Psoriasis response elements (PREs) most strongly enriched in genomic sequences upstream of psoriasis DEGs. We screened 2935 binding sites (PWM matrix models) to identify PRE motifs most significantly enriched in 5KB regions upstream of psoriasis DEGs. (A – C) Top 12 motifs enriched with respect to (A) PP-increased DEGs, (B) PP-decreased DEGs, and (C) all psoriasis DEGs, respectively. For each motif, enrichment is proportional to the Z statistic obtained from semiparametric generalized additive logistic modeling (see Methods). The ratio between the number of motif occurrences in regions upstream of psoriasis DEGs and the number of occurrences among other skin-expressed genes is listed (Ratio). Labels in red or blue font (left margin) denote cases in which the motif is recognized by a protein encoded by a PP-increased DEG or PP-decreased DEG, respectively. (D) Comparison between enrichment Z statistics obtained with respect to PP-increased DEGs and PP-decreased DEGs (n = 2935 PWM models). The yellow circle outlines the 50% of values closest to the centroid (Mahalanobis distance). (E) PWM sequence logos associated with top-ranking motifs from (A) – (C). [file 40169_2015_54_MOESM13_ESM.tiff]

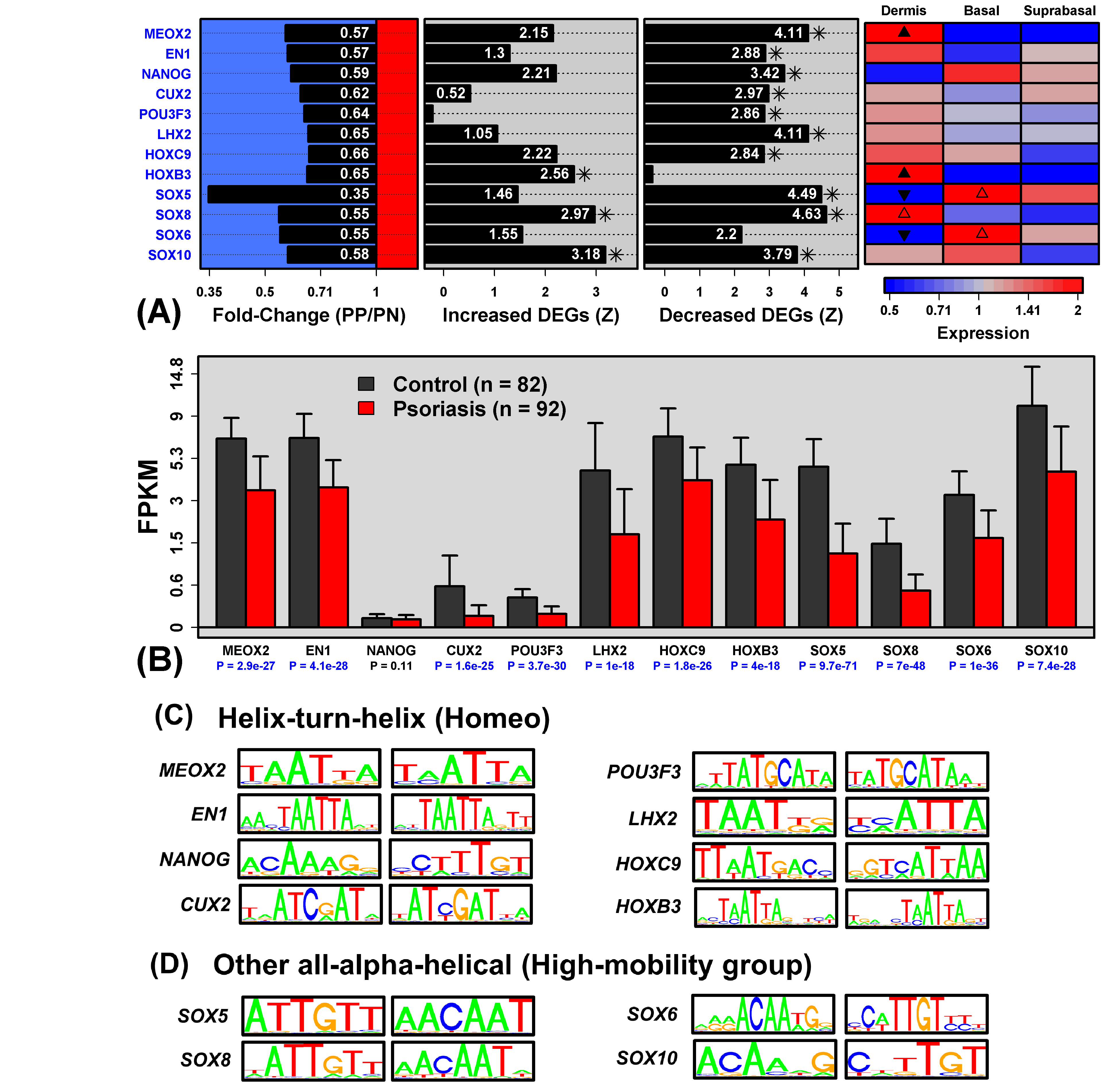

Supplement: Additional file 14: — PREs upstream of PP-decreased DEGs interact with helix-turn-helix (homeo) and other all-alpha-helical (high-mobility group) DNA-binding domains. (A) TFs encoded by helix-turn-helix (homeo) and other all-alpha-helical (high-mobility group) DNA-binding domains show decreased expression in psoriasis (n = 237 patients; microarray). Most PP-decreased TFs with these domains recognize DNA elements enriched in sequences upstream of PP-increased and/or PP-decreased DEGs (see Z statistics; middle two figures). The right-most figure shows relative expression in dermis, suprabasal epidermis and basal epidermis (laser capture microdissection, GSE42114). (B) Mean expression in lesional (PP) and normal skin (NN) from control subjects (RNA-seq; GSE54456). Expression is measured using fragments per kilobase of transcript per million mapped reads (FPKM). (C) Sequence logos for helix-turn-helix (homeo) TFs. (D) Sequence logos for other all-alpha-helical (high-mobility group) TFs. [file 40169_2015_54_MOESM14_ESM.tiff]

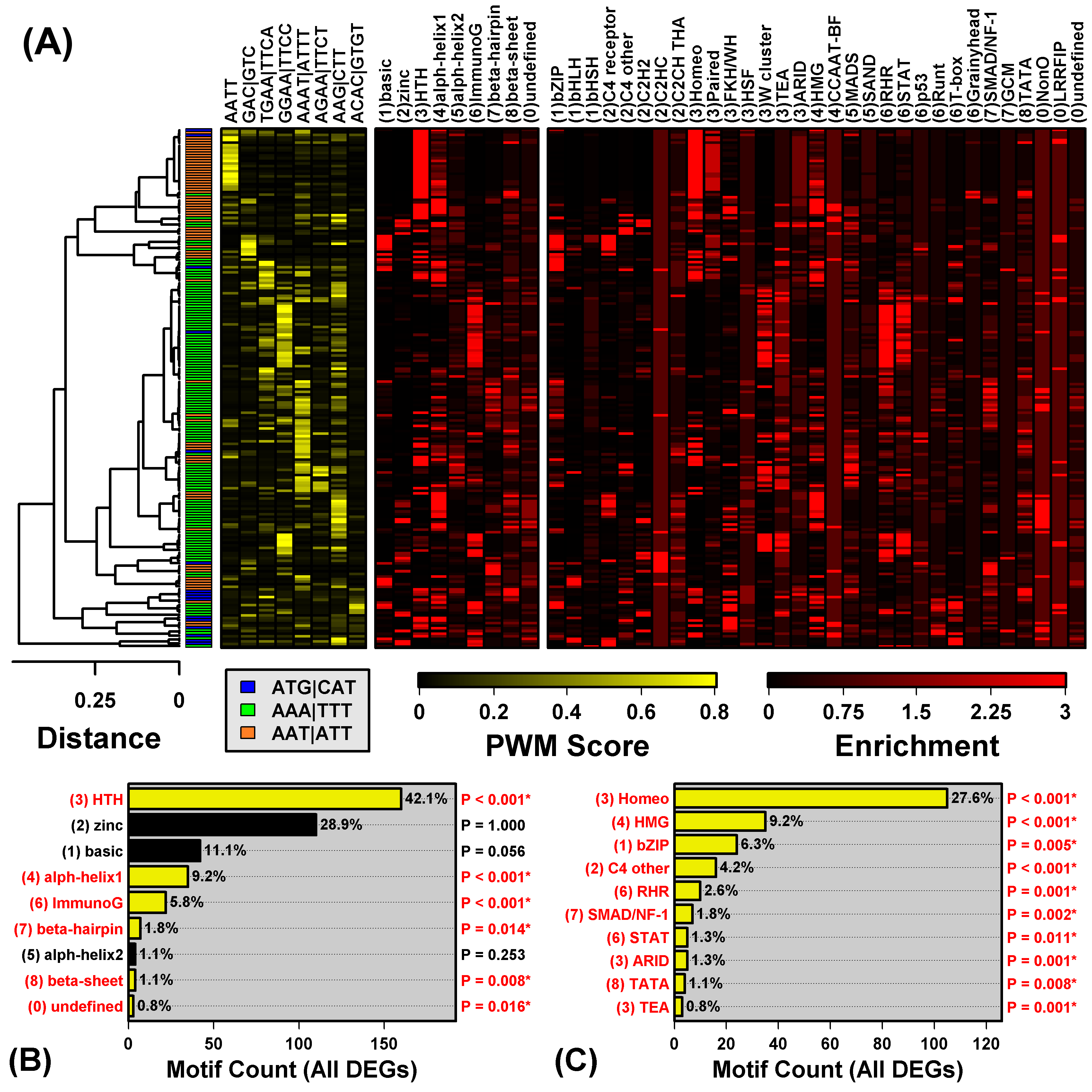

Supplement: Additional file 15: — PRE motifs significantly enriched in sequences upstream of psoriasis-increased and psoriasis-decreased DEGs. We identified 462 PWM models matching motifs significantly enriched in sequences upstream of PP-increased and PP-decreased DEGs (FDR < 0.10). (A) The 200 most significantly enriched motifs were clustered as described in Figure 2, leading to the identification of three motif sub-groups. The yellow-black heat map shows k-mer scores for each motif (top margin). Red-black heatmaps show enrichment scores indicating how well a given PWM matches others associated with DNA-binding domain superfamily and class groups (TFclass database). [file 40169_2015_54_MOESM15_ESM.tiff]

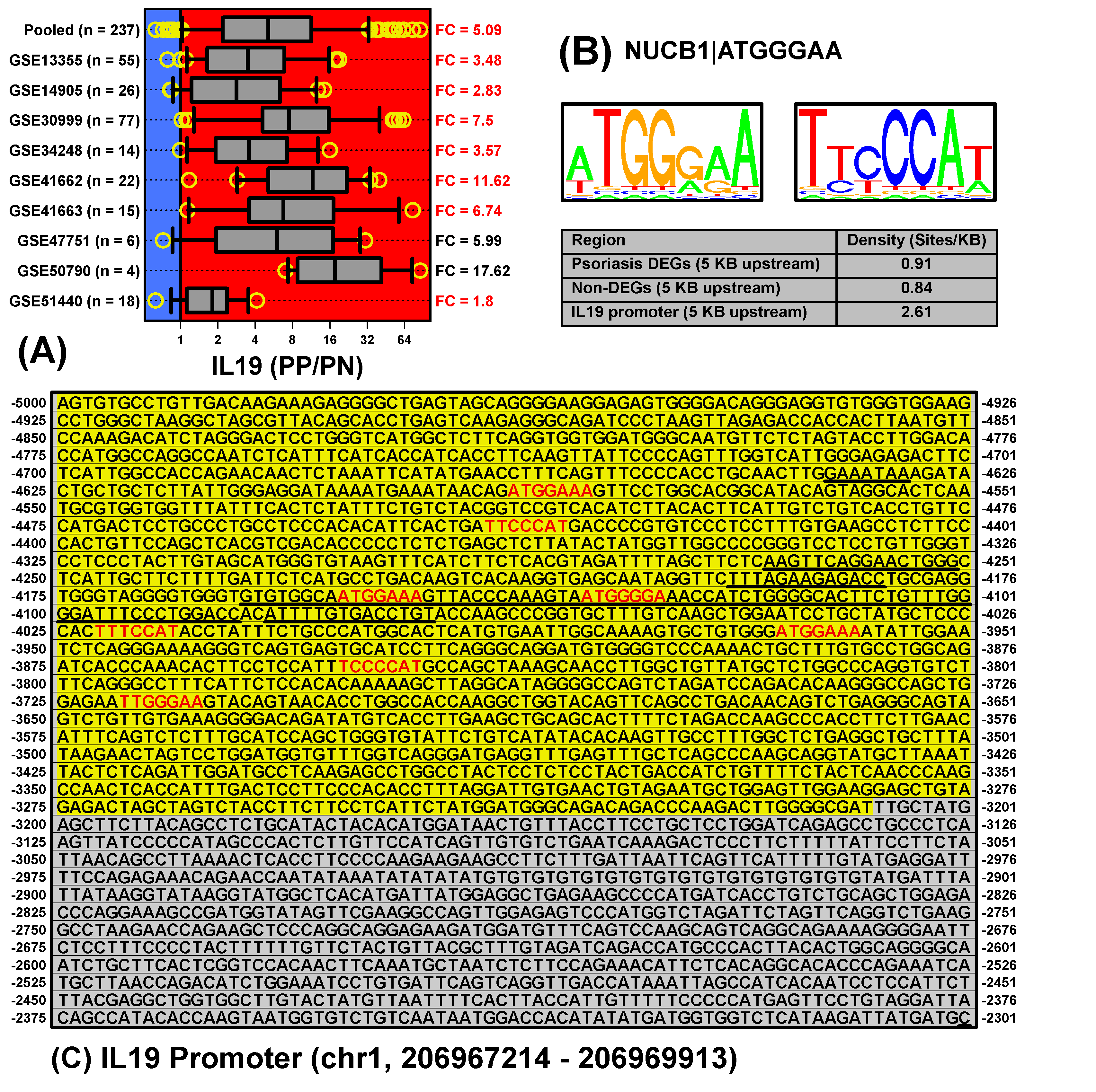

Supplement: Additional file 16: — PRE motifs are prominent in the IL19 promoter and present within an upstream enhancer region. (A) IL19 expression is significantly elevated in psoriasis lesions. Grey boxes outline the middle 50% of fold-change (FC) estimates for each dataset (whiskers: middle 90%; yellow symbols: extreme values). The median FC for each dataset is listed (right margin; FDR < 0.05 for red labels). (B) Sequence logos for the NUCB1 motif significantly overrepresented in sequence regions upstream of psoriasis DEGs. The motif’s frequency is elevated within the IL19 promoter (see table). (C) IL19 promoter (chr1, 206967214–206969913). NUCB1 motif matches (red font) and conserved elements are indicated (underlined, phastcons ≥ 0.50). Yellow highlighted sequence denotes H4k20me1 histone modification (NHEKs). [file 40169_2015_54_MOESM16_ESM.tiff]

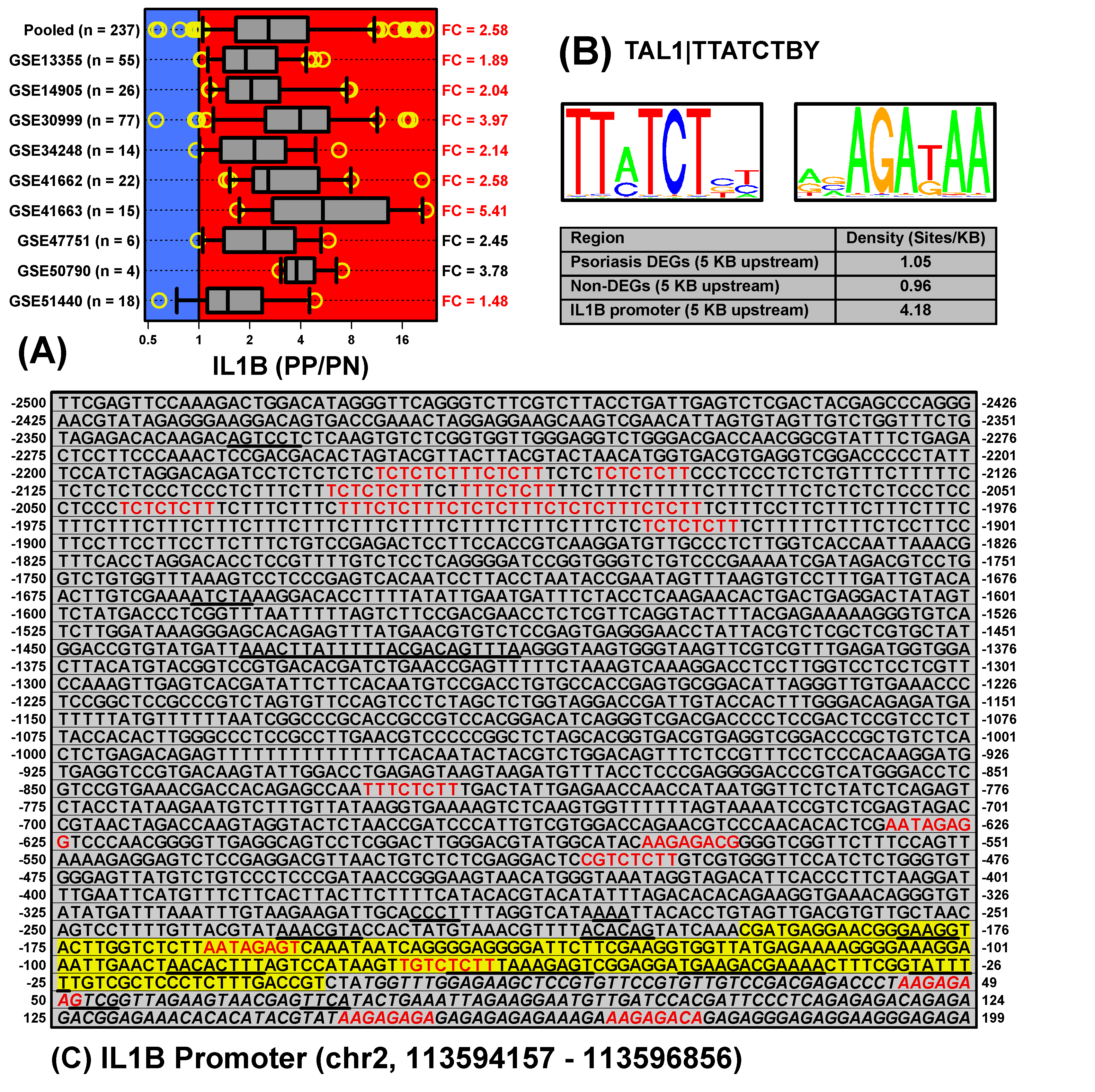

Supplement: Additional file 17: — PRE motifs are prominent in the IL1B promoter and present within an upstream enhancer region. (A) IL1B expression is significantly elevated in psoriasis lesions. Grey boxes outline the middle 50% of fold-change (FC) estimates for each dataset (whiskers: middle 90%; yellow symbols: extreme values). The median FC for each dataset is listed (right margin; FDR < 0.05 for red labels). (B) Sequence logos for the TAL1 motif significantly overrepresented in sequence regions upstream of psoriasis DEGs. The motif’s frequency is elevated within the IL1B promoter (see table). (C) IL1B promoter (chr2, 113594157–113596856). TAL1 motif matches (red font) and conserved elements are indicated (underlined, phastcons ≥ 0.50). Yellow highlighted sequence denotes a DNase I hypersensitive site and Faire-seq peak (NHEKs). [file 40169_2015_54_MOESM17_ESM.tiff]

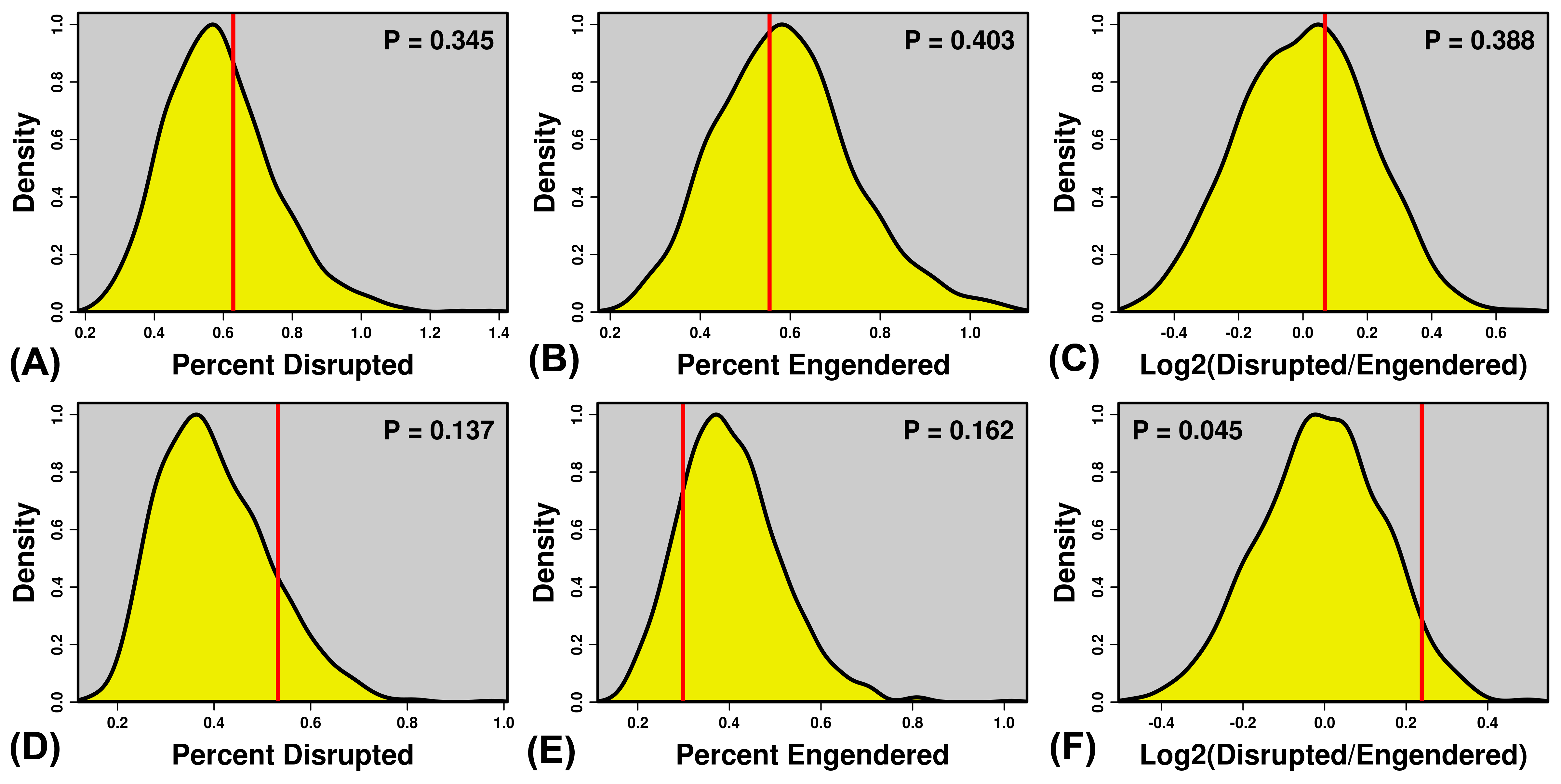

Supplement: Additional file 19: — Variants at psoriasis-associated non-coding/enhancer SNP loci disproportionately disrupt PRE motifs in sequences upstream of PP-decreased DEGs (simulation analysis). Simulation was used to assess the effects of risk variants at psoriasis-associated SNP loci on PRE motifs in comparison to the effects of genetic variants at randomly sampled SNP loci (1000 trials). We considered 53 psoriasis-associated non-coding SNPs within NHEK enhancers and the effects of associated risk variants on PRE motif matches. In each simulation trial, 53 SNPs were randomly chosen from a larger pool of 1.82 million SNPs, which was generated by identifying autosomal SNPs positioned within non-coding NHEK enhancer regions and located at least 500 kb from any psoriasis-associated SNP (at least 4 Mb in the MHC region). The 53 random SNPs were frequency-matched with respect to the 53 psoriasis-associated SNPs. For each random SNP, one associated genetic variant was randomly designated as the risk allele. Analyses were performed with respect to the 126 PRE motifs enriched in sequences upstream of PP-increased DEGs (parts A – C), as well as the 461 PRE motifs enriched in sequences upstream of PP-decreased DEGs (parts D – F). In each trial, we evaluated the percentage of SNP-PRE combinations in which a PRE match was disrupted (parts A and D) or engendered (parts B and E), as well as the ratio of disrupted to engendered matches (parts C and F). Figures show the null distribution generated by random SNP sampling in relation to the corresponding value calculated with respect to the 53 psoriasis-associated SNPs (red vertical line). P-values indicate the proportion of the null distribution for which values are more extreme than those calculated based upon the 53 psoriasis-associated SNPs (one-sided hypothesis test). [file 40169_2015_54_MOESM19_ESM.tiff]
