# Supplementary material for: Psoriasis drug development and GWAS interpretation through in silico analysis of transcription factor binding sites
Source: Clin Transl Med. 2015 Mar 19;4:13. doi: 10.1186/s40169-015-0054-5 (PMC4392043; doi:10.1186/s40169-015-0054-5)
Supplement: Additional file 9: — TF-encoding DEGs are more likely to interact with PRE motifs than TF-encoding non-DEGs. Our analysis identified 1149 TF-encoding genes expressed in human skin, including 39 PP-increased DEGs, 67 PP-decreased DEGs, and 1043 non-DEGs with similar expression in lesional and uninvolved skin. We evaluated whether TF-encoding DEGs are more likely to interact with PRE motifs than TF-encoding non-DEGs. The analysis was performed with respect to TF-encoding PP-increased DEGs (n = 39), PP-decreased DEGs (n = 67) and both PP-increased + PP-decreased DEGs (n = 106); additionally, analyses were performed with respect to PRE motifs enriched upstream of PP-increased DEGs (n = 126 PREs), PP-decreased DEGs (n = 461), and the combined set of all DEGs (n = 462). For each row of the table, the percentage of TF-encoding DEGs associated with a PRE motif was compared with that observed among TF-encoding non-DEGs (Fisher’s Exact Test). [file 40169_2015_54_MOESM9_ESM.pdf]

Additional File 9. TF-encoding DEGs are more likely to interact with PRE motifs than TF-encoding non-DEGs. Our analysis identified 1149 TF-encoding genes expressed in human skin, including 39 PP-increased DEGs, 67 PP-decreased DEGs, and 1043 non-DEGs with similar expression in lesional and uninvolved skin. We evaluated whether TF-encoding DEGs are more likely to interact with PRE motifs than TF-encoding non-DEGs. The analysis was performed with respect to TF-encoding PP-increased DEGs (n = 39), PP-decreased DEGs (n = 67) and both PP-increased + PP-decreased DEGs (n = 106); additionally, analyses were performed with respect to PRE motifs enriched upstream of PP-increased DEGs (n = 126 PREs), PP-decreased DEGs (n = 461), and the combined set of all DEGs (n = 462). For each row of the table, the percentage of TF-encoding DEGs associated with a PRE motif was compared with that observed among TF-encoding non-DEGs (Fisher's Exact Test).

TF DEG Group	PRE Group	% PRE-Associated (DEGs)[1]	% PRE-Associated (non-DEGs)[2]	P-Value
Increased (n = 39)	Increased (n = 126)	8/39 (20.5%)	64/1110 (5.8%)	0.000417*
Decreased (n = 67)	Increased (n = 126)	4/67 (6%)	68/1082 (6.3%)	0.412
Both (n = 106)	Increased (n = 126)	12/106 (11.3%)	60/1043 (5.8%)	0.0112*
Increased (n = 39)	Decreased (n = 461)	5/39 (12.8%)	181/1110 (16.3%)	0.624
Decreased (n = 67)	Decreased (n = 461)	17/67 (25.4%)	169/1082 (15.6%)	0.0152*
Both (n = 106)	Decreased (n = 461)	22/106 (20.8%)	164/1043 (15.7%)	0.0731
Increased (n = 39)	Both (n = 462)	7/39 (17.9%)	178/1110 (16%)	0.283
Decreased (n = 67)	Both (n = 462)	11/67 (16.4%)	174/1082 (16.1%)	0.391
Both (n = 106)	Both (n = 462)	18/106 (17%)	167/1043 (16%)	0.338

[1]Fraction of TF-encoding DEGs interacting with PREs (FDR < 0.10).

[2]Fraction of TF-encoding non-DEGs interacting with PREs (FDR < 0.10).
