# Supplementary material for: Psoriasis drug development and GWAS interpretation through in silico analysis of transcription factor binding sites
Source: Clin Transl Med. 2015 Mar 19;4:13. doi: 10.1186/s40169-015-0054-5 (PMC4392043; doi:10.1186/s40169-015-0054-5)
Supplement: Additional file 18: — List of 91 unique TF decoy oligonucleotides (dODNs). A literature review identified 167 dODNs used and validated in prior studies. These were screened to exclude redundant dODNs with the same sequence, yielding a set of 91 unique dODNs. For those dODNs reported by multiple publications, the table in this file lists the earliest publication reporting the dODN sequence. [file 40169_2015_54_MOESM18_ESM.pdf]

Additional File 18. List of 91 unique TF decoy oligonucleotides (dODNs). A literature review identified 167 dODNs used and validated in prior studies. These were screened to exclude redundant dODNs with the same sequence, yielding a set of 91 unique dODNs listed in the table below. For those dODNs reported by multiple publications, the table below lists the earliest publication in which the dODN sequence was reported.

Target (PubMed ID)	Sense Sequence (5'-3')	Antisense Sequence (5'-3')	Reference
AGF2 (8613193)	CCCAGCCTCTGTACAGAGTAGCCC A	TGGGCTACTCTGTACAGAGGCT GGG	Morishita et al. 1996, Hypertension 27:502-7
AGF3 (8613193)	AGGGGATAGCTGTGCTTGTCTAGG TT	AACCTAGACAAGCACAGCTATC CCCT	Morishita et al. 1996, Hypertension 27:502-7
AP1 (10918504)	AGCTTGTGAGTCAGAAGCT	AGCTTCTGACTCACAAGCT	Tomita et al. 2000, Gene Ther 7:1326-32
AP1 (11024008)	CGCTTGATGACTCAGCCGGAA	TTCCGGCTGAGTCATCAAGCG	Viedt et al. 2000, FASEB J 14:2370-2
AP1 (11895002)	GATCCTGTACAGGATGTTCTAGCT ACA	TGTAGCTAGAACATCCTGTACA GGATC	Yoshimura et al. 2001, Gene Ther 8:1635-42
AP1 (12089071)	GGATCCATGACTCAGAAGACGAC ACACGTCTTCTGAGTCAT	ATGACTCAGAAGACGTGTGTCG TCTTCTGAGTCATGGATCC	Ahn et al. 2002, Circ Res 90:1325-32
AP1 (12089071)	AGCTTGTGACTCAGAAGCT	AGCTTCTGAGTCACAAGCT	Ahn et al. 2002, Circ Res 90:1325-32
AP1 (12089071)	GGATCCAAATCTCAGAAGACGAC ACACGTCTTCTGAGATTT	AAATCTCAGAAGACGTGTGTCG TCTTCTGAGATTTGGATCC	Ahn et al. 2002, Circ Res 90:1325-32
AP1 (17890327)	GGAACATGAGTTCATCTATTTC	GAAATAGATGAACTCATGTTCC	Vayalil et al. 2007, Am J Physiol Lung Cell Mol Physiol 293:L1281-92
AP1 (18458670)	TGTCTGACTCATGTC	GACATGAGTCAGACA	Moriyama et al. 2008, Lab Invest 88:652-63
AP1 (22258905)	AAACATATGATTCACCAGGCA	TGCCTGGTGAATCATATGTTT	Börner et al. 2012, Mol Pharmacol 81:587-97
AP1 (22258905)	TTACCTATGAGTTATCTGTTT	AAACAGATAACTCATAGGTAA	Börner et al. 2012, Mol Pharmacol 81:587-97
AP1 (22772035)	TGTGATGACTCAGGTTTG	CAAACCTGAGTCATCACA	Finotti et al. 2012, Artif DNA PNA XNA 3:97-296
AP1 (23223130)	GTGTCTGACTCATGTACTGTCTTA CCTCATGTC	GACATGAGGTAAGACAGTACAT GAGTCAGACAC	Yuan et al. 2013, J Invest Dermatol 133:1080-7
C/EBP (12067903)	TGCAGATTGCGCAATCTGCA	TGCAGATTGCGCAATCTGCA	Kelkenberg et al. 2002, Arterioscler Thromb Vasc Biol

			22:949-54
C/EBP (22851691)	TGCAGATTGCACAATCTG	CAGATTGTGCAATCTGCA	Jung et al. 2012, Mol Cell Biol 32:3938-48
CHOP (22772035)	CGCTGGTGTGATGCACGG	CCGTGCATCACACCAGCG	Finotti et al. 2012, Artif DNA PNA XNA 3:97-296
CREB (22772035)	AAAACTTTCGTCATACTC	GAGTATGACGAAAGTTTT	Finotti et al. 2012, Artif DNA PNA XNA 3:97-296
CREB (23825534)	TGACGTCATGACGTCATGACGTCA	TGACGTCATGACGTCATGACGT CA	Chou et al. 2013, PLoS ONE 8:e66268
CREB (23840351)	TGACGTCAGAGAGCGCTCTCTGAC GTCA	TGACGTCAGAGAGCGCTCTCTG ACGTCA	Wang et al. 2013, PLoS ONE 8:e65661
E2F (9691019)	CTAGATTTCCCGCG	CGCGGGAAATCTAG	Tomita et al. 1998, Am J Physiol 275:F278-84
E2F (12457282)	GGATCCGTTTCGCGCTATTGCAAA AGCAATAGCGCGAAAC	GTTTCGCGCTATTGCTTTTGCAA TAGCGCGAAACGGATCC	Ahn et al. 2002, Gene Ther 9:1682-92
E2F (12457282)	ATTTAAGTTTCGCGCCCTTTCTC	GAGAAAGGGCGCGAAACTTAA AT	Ahn et al. 2002, Gene Ther 9:1682-92
EGR-1 (14712296)	CCGAGAGCGGGGGCGAGCGTG	CACGCTCGCCCCCGCTCTCGG	Ohtani et al. 2004, Gene Ther 11:126-32
EGR-1 (22906951)	GGATCCAGCGGGGGCGAGCGGGG GCGA	TCGCCCCCGCTCGCCCCCGCTG GATCC	Cattaruzza et al. 2012, FASEB J 26:4864-75
ETS1 (17885220)	AATTCACCGGAAGTATTCGA	TCGAATACTTCCGGTGAATT	Miyake et al. 2007, Circ Res 101:1175-84
HIF1 (14980978)	GCCCTACGTGCTGTCTCA	TGAGACAGCACGTAGGGC	Yang et al. 2004, Br J Pharmacol 141:988-96
HSF1 (11423124)	CTAGAAGCTTCTAGAAGCTTCTAG	CTAGAAGCTTCTAGAAGCTTCT AG	Ianaro et al. 2001, FEBS Lett 499:239-44
IRF1 (10620190)	ATATTTCACTTTCATAATGGAAAA TTC	GAATTTTCCATTATGAAAGTGA AATAT	Amoah-Apraku et al. 2000, Kidney Int 57:83-91
IRF1 (11781233)	GGAAGCGAAAATGAAATTGAC	GTCAATTTCATTTTCGCTTCC	Wagner et al. 2002, Blood 99:520-5
MAZ (21931711)	CGAGGCCGGGGCGGGGCGGGGGC GGGGGCGCGCGGT	ACCGCGCGCCCCCGCCCCCGCC CCGCCCCGGCCTCG	Membrino et al. 2011, PLoS ONE 6:e24421
MAZ (21931711)	CGGCTCGGGTTGCGGGCGCAGGG CACGGGCGGC	GCCGCCCGTGCCCTGCGCCCGC AACCCGAGCCG	Membrino et al. 2011, PLoS ONE 6:e24421
MAZ (21931711)	TCGGGTTGCGGGCGCAGGGCACG	CGCCCGTGCCCTGCGCCCGCAA	Membrino et al. 2011, PLoS ONE

	GGCG	CCCGA	6:e24421
MAZ (21931711)	TCGGGTTGCGGGCGCAGGGCACGGGCGG	CCGCCCGTGCCCTGCGCCCGCAACCCGA	Membrino et al. 2011, PLoS ONE 6:e24421
MAZ (21931711)	CGGGGCGGGGCGGGGGCGGGGGCG	CGCCCCCGCCCCGCCCCGCCCCG	Membrino et al. 2011, PLoS ONE 6:e24421
MAZ (21931711)	GCGGTGTCGCAAGACGCAAGACGCGGAGGCAG	CTGCCTCCGCGTCTTGCGTCTTGCGACACCGC	Membrino et al. 2011, PLoS ONE 6:e24421
MAZ (23471001)	GCGGTGTGGGAAGAGGGAAGAGGGGGAGGCAG	CTGCCTCCCCCTCTTCCCTCTTCCCACACCGC	Cogoi et al. 2013, Nucleic Acids Res 41:4049-64
MAZ (23471001)	GCGGTGTGGAAAGAGGGAAGAGGGGGAGGCAG	CTGCCTCCCCCTCTTCCCTCTTTCCACACCGC	Cogoi et al. 2013, Nucleic Acids Res 41:4049-64
MAZ (23471001)	GCGGTGTGGGAAGAGGGGGGGGAGGCAG	CTGCCTCCCCCCCCTCTTCCCACACCGC	Cogoi et al. 2013, Nucleic Acids Res 41:4049-64
MAZ (23471001)	GCGGTGTGGGGGGAAGAGGGGGAGGCAG	CTGCCTCCCCCTCTTCCCCCCACACCGC	Cogoi et al. 2013, Nucleic Acids Res 41:4049-64
MAZ (23471001)	GCGGTGTGGGGGGGGGGAGGCAG	CTGCCTCCCCCCCCCCACACCGC	Cogoi et al. 2013, Nucleic Acids Res 41:4049-64
MAZ (23471001)	GCGGTGTCGCAAGACGCAAGACGCGGAGGCCG	CGGCCTCCGCGTCTTGCGTCTTGCGACACCGC	Cogoi et al. 2013, Nucleic Acids Res 41:4049-64
NFAT (22906951)	CGCCCAAAGAGGAAAATTTGTTTCATA	TATGAAACAAATTTTCCTCTTTGGGCG	Cattaruzza et al. 2012, FASEB J 26:4864-75
NF-IL6 (22772035)	CATCAGTTGCAAATCGTGG	CCACGATTTGCAACTGATG	Finotti et al. 2012, Artif DNA PNA XNA 3:97-296
NFkB (10341880)	CCTTGAAGGGATTTCCCTCC	GGAGGGAAATCCCTTCAAGG	Kawamura et al. 1999, Gene Ther 6:91-7
NFkB (10744638)	AGTTGAGGGGACTTTCCCAGGC	GCCTGGGAAAGTCCCCTCAACT	Vos et al. 2000, FASEB J 14:815-22
NFkB (10864580)	CTTGAAGGGATTTCCCTCC	GGAGGGAAATCCCTTCAAG	Tomita et al. 2000, J Am Soc Nephrol 11:1244-52
NFkB (10933964)	AGGGACTTTCCGCTGGGGACTTTCC	GGAAAGTCCCCAGCGGAAAGTCCCT	Giannoukakis et al. 2000, Mol Ther 1:430-7
NFkB (11083494)	GATCGAGGGGACTTTCCCTAGC	GCTAGGGAAAGTCCCCTCGATC	D'Acquisto et al. 2000, Gene Ther 7:1731-7
NFkB (17328896)	CTGGAAAGTCCCTCGAAGAGGGACTTTCCAG	CTGGAAAGTCCCTCTTCGAGGGACTTTCCAG	Laguillier et al. 2007, FEBS Lett 581:1143-50
NFkB (22258905)	AAAAAAAAGGGACTTTCATTGTAC	ACCAGTACAATGAAAGTCCCTT	Börner et al. 2012, Mol Pharmacol

	TGGT	TTTTTT	81:587-97
NFkB (22258905)	GTGGGAGGGGGCTATACGCAGAG G	CCTCTGCGTATAGCCCCCTCCC AC	Börner et al. 2012, Mol Pharmacol 81:587-97
NFkB (22772035)	AGAGGAATTTCCACGATT	AATCGTGGAAATTCCTCT	Finotti et al. 2012, Artif DNA PNA XNA 3:97-296
NFkB (23056313)	GATCGAGGGGACTTTCCCTAG	CTAGGGAAAGTCCCCTCGATC	Ungaro et al. 2012, PLoS ONE 7:e46457
NRF2 (24949080)	CTAATGGTGACAAAGCAACTTT	AAAGTTGCTTTGTCACCATTAG	Qi et al. 2014, Evid Based Complement Alternat Med 2014:945814
NRSF (21905079)	GGAGCTGTCCACAGTTCTGAA	TTCAGAACTGTGGACAGCTCC	McClelland et al. 2011, Ann Neurol 70:454-64
SMAD (17890327)	GGGAGAGACAGACACAGGCAG	CTGCCTGTGTCTGTCTCTCCC	Vayalil et al. 2007, Am J Physiol Lung Cell Mol Physiol 293:L1281-92
SMAD (23223130)	GTCTGAGCCAGACATAGTGATGCA GACATACTC	GAGTATGTCTGCATCACTATGT CTGGCTCAGAC	Yuan et al. 2013, J Invest Dermatol 133:1080-7
SMAD (23791891)	CTGTAACAAAAGTTACAGACCGA ACAGAAAACTGTTCGGT	ACCGAACAGTTTTCTGTTCGGT CTGTAACTTTTGTTACAG	Sung et al. 2013, Exp Mol Pathol 95:136-43
SMAD (23791891)	GTAACCAAGCCAGACATTGAAAA CAATGTCTGGCTT	AAGCCAGACATTGTTTTCAATG TCTGGCTTGGTTAC	Sung et al. 2013, Exp Mol Pathol 95:136-43
SP1 (15342415)	ATTCCGGGGGCGGGGGCATG	CATGCCCCCGCCCCCGGAAT	Uchida et al. 2004, Cancer Res 64:6271-9
SP1 (17890327)	GTGGGTGGGGCTGGAACAT	ATGTTCCAGCCCCACCCAC	Vayalil et al. 2007, Am J Physiol Lung Cell Mol Physiol 293:L1281-92
SP1 (22023388)	ATTACGAGGCGGGGGGGCTAC	GTAGCCCCCCCGCCTCGTAAT	Li et al. 2012, Biochem J 441:859-67
SP1 (22906951)	ATTCGATCGGGGCGGGGCGAGC	GCTCGCCCCGCCCCGATCGAAT	Cattaruzza et al. 2012, FASEB J 26:4864-75
SP1 (23338822)	GCCCCGATCTTTTGATCGGGGCGG GGCGAGCTTTTGCTCGCCCC	GGGGCGAGCAAAAGCTCGCCCC GCCCCGATCAAAAGATCGGGGC	Deng et al. 2013, Mol Med Rep 7:785-90
SP1 (23791891)	CCCCAACAAAAGTTGGGGCGGGG ACAGAAAACTGTCCCCG	CGGGGACAGTTTTCTGTCCCCG CCCCAACTTTTGTTGGGG	Sung et al. 2013, Exp Mol Pathol 95:136-43
SP1 (23791891)	GGTTACAGGGGCGGGGTTCAAAA GAACCCCGCCCCT	AGGGGCGGGGTTCTTTTGAACC CCGCCCCTGTAACC	Sung et al. 2013, Exp Mol Pathol 95:136-43

STAT1 (10432321)	ATATTCCTGTAAGTG	CACTTACAGGAATAT	Huang et al. 1999, Biochem J 342:231-8
STAT1 (11781233)	CATGTTATGCATATTCCTGTAAGTG	CACTTACAGGAATATGCATAACATG	Wagner et al. 2002, Blood 99:520-5
STAT1 (17981315)	TGTGAATTACCGGAAGTG	CACTTCCGGTAATTCACA	Wagner et al. 2008, J Allergy Clin Immunol 121:158-165
STAT3 (10432321)	GATCCTTCTGGGAATTCCTAGATC	GATCTAGGAATTCCCAGAAGGATC	Huang et al. 1999, Biochem J 342:231-8
STAT3 (17683579)	CATTTCCCGTAAATC	GATTTACGGGAAATG	Zhang et al. 2007, BMC Cancer 7:149
STAT3 (21486470)	CATTTCCCGTAAATCGAAGATTTACGGGAAATG	CATTTCCCGTAAATCTTCGATTTACGGGAAATG	Souissi et al. 2011, BMC Cell Biol 12:14
STAT3 (22423663)	TATTTCCCCTAAATGGAACATTTAGGGGAAATA	TATTTCCCCTAAATGTTCCATTTAGGGGAAATA	Souissi et al. 2012, Mol Cancer 11:12
STAT3 (22423663)	CATTTCCCCTAAATCGAAGATTTAGGGGAAATG	CATTTCCCCTAAATCTTCGATTTAGGGGAAATG	Souissi et al. 2012, Mol Cancer 11:12
STAT3 (22423663)	GCTTTCCCCTAAATGGAACATTTAGGGGAAAGC	GCTTTCCCCTAAATGTTCCATTTAGGGGAAAGC	Souissi et al. 2012, Mol Cancer 11:12
STAT3 (22719020)	CATTTCCCGTTAATC	GATTAACGGGAAATG	Sen et al. 2012, Cancer Discov 2:694-705
STAT5 (21091189)	AGATTTCTAGGAATTCAAATC	GATTTGAATTCCTAGAAATCT	Wang et al. 2011, DNA Cell Biol 30:71-8
STAT5 (23146666)	GATCGCATTTCGGAGAAGACG	CGTCTTCTCCGAAATGCGATC	Busch-Dienstfertig et al. 2012, Mol Pain 8:83
STAT6 (23146666)	GATCCTACTTCATGGAAGAAT	ATTCTTCCATGAAGTAGGATC	Busch-Dienstfertig et al. 2012, Mol Pain 8:83
ARNTL/CLOCK (23291558)	TTTAGCCACGTGACAGTGTAACGACACGTGGGCCCTCAAGTCCACGTGCAGGGA	TCCCTGCACGTGGACTTGAGGGCCCACGTGTCGTTACACTGTCACGTGGCTAAA	Xu et al. 2013, Toxicol Sci 132:368-78
AP1/SMAD (23223130)	GTGTCTGCATGACTCAGCCAGACATGAGGTAAC	GTTACCTCATGTCTGGCTGAGTCATGCAGACAC	Yuan et al. 2013, J Invest Dermatol 133:1080-7
AP1/SMAD (23223130)	GTGACTCATGCAGACATGAGGTAAGCCAGACAC	GTGTCTGGCTTACCTCATGTCTGCATGAGTCAC	Yuan et al. 2013, J Invest Dermatol 133:1080-7
AP1/SMAD (23223130)	GATGCAGACATGAGGTAAGCCAGACATGACTCAC	GTGAGTCATGTCTGGCTTACCTCATGTCTGCATC	Yuan et al. 2013, J Invest Dermatol 133:1080-7
AP1/SMAD	GAGCCAGACATGAGTCATGTCTGC	GTGAGGTAATGCAGACATGACT	Yuan et al. 2013, J Invest Dermatol

(23223130)	ATTACCTCAC	CATGTCTGGCTC	133:1080-7
cMYC/Oct4/Sox2 (24040121)	CATTGTTGCATTACAATGCTAATAGCACGTG	CACGTGCTATTAGCATTGTAATGCAACAATG	Wang et al. 2013, PLoS ONE 8:e73942
E2F/NFkB (18515844)	GAAGGGATTTCCCTCCATTTCCCGCGGA	TCCGCGGGAAATGGAGGGAAATCCCTTC	Miyake et al. 2008, Cardiovasc Res 79:706-14
E2F/NFkB/STAT3 (16936227)	TCTGAGCTTCTGGGAACTTGGGGACTTTCGCGCCCTA	TAGGGCGCGAAAGTCCCCAAGTTCCCAGAAGCTCAGA	Gao et al. 2006, Mol Pharmacol 70:1621-9
ETS1/NFkB (14662712)	ACCGGAAGTATGAGGGATTTCCCTCC	GGAGGGAAATCCCTCATACTTCCGGT	Nakashima et al. 2004, Circulation 109:132-8
SMAD/SP1 (23791891)	CAATGTCTGACTTGGTTACAGGGGCGGGGTTCAAG	CTTGAACCCCGCCCCTGTAACCAAGTCAGACATTG	Sung et al. 2013, Exp Mol Pathol 95:136-43
STAT1/STAT3 (23146666)	GATCGAGTTTACGAGAACTC	GAGTTCTCGTAAACTCGATC	Busch-Dienstfertig et al. 2012, Mol Pain 8:83
